# Design and development of dual targeting CAR protein for the development of CAR T-cell therapy against KRAS mutated pancreatic ductal adenocarcinoma using computational approaches

**DOI:** 10.1007/s12672-024-01455-6

**Published:** 2024-10-25

**Authors:** Prasanna Srinivasan Ramalingam, T. Premkumar, Vino Sundararajan, Md Sadique Hussain, Sivakumar Arumugam

**Affiliations:** 1 Protein Engineering Lab, School of Biosciences and Technology, Vellore Institute of Technology, Vellore, Tamil Nadu India; 2 Integrative Multiomics Lab, School of Bio-Sciences & Technology, Vellore Institute of Technology, Vellore, Tamil Nadu India; 3 Uttaranchal Institute of Pharmaceutical Sciences, Uttaranchal University, Dehradun, Uttarakhand 248007 India

**Keywords:** KRAS, Therapeutics, CAR, CAR T-cell therapy, Immunotherapy, Precision medicine

## Abstract

Mutant KRAS promotes the proliferation, metastasis, and aggressiveness of various cancers including pancreatic ductal adenocarcinoma (PDAC), non-small cell lung cancer (NSCLC), and colorectal adenocarcinoma (CRC) respectively. Mutant KRAS therapeutics are limited, while Sotorasib and Adagrasib were the only FDA-approved drugs for the treatment of KRAS^G12C^ mutated NSCLC. Chimeric antigen receptor (CAR) T-cell therapy has been emerged as an effective strategy against hematological malignancies and being extended towards solid cancers including PDAC. mesothelin (MSLN) and Carcinoembryonic Antigen (CEA) were reported to be highly overexpressed in KRAS-mutated PDAC. Meanwhile, in clinical trials, several CAR T-cell therapy studies are mainly focused towards these two cancer antigens in PDAC, however, the dual targeting of these two neoantigens is not reported. In the present study, we have designed and developed a novel dual-targeting CAR protein by employing various bioinformatics approaches such as functional analysis (antigenicity, allergenicity, antigen binding sites & signalling cascades), qualitative analysis (physicochemical, prediction, refinement & validation of 2D and 3D structures), molecular docking, and in silico cloning. Our results revealed that the designed CAR protein specifically binds with both MSLN & CEA with significant binding affinities, and was predicted to be stable & non-allergenic. Additionally, the protein–protein interaction network reveals the T-cell mediated antitumor responses of each domain in the designed CAR. Conclusively, we have designed and developed a dual targeting (MSLN & CEA) CAR protein towards KRAS-mutated PDAC using computational approaches. Alongside, we further recommend to engineer this designed CAR in T-cells and evaluating their therapeutic efficiency in in vitro and in vivo studies in the near future.

**Supplementary Information:**

The online version contains supplementary material available at 10.1007/s12672-024-01455-6.

## Introduction

KRAS is a small GTPase that acts as a molecular switch by toggling between GTP-bound active state (ON) and GDP-bound inactive state (OFF) and further facilitates the downstream signal transduction of MAPK and PI3K-Akt signalling pathways [1–3]. Mutations at codons 12, 13, and 61 of KRAS protein are common, and notably, the G12D and G12C are more frequent mutations [4, 5]. The mutated KRAS holds the GTP-bound active state & overcomes the GTPase activity (GTP to GDP hydrolysis), which further continuously promotes cellular proliferation and metastasis in various cancers such as pancreatic ductal adenocarcinoma (PDAC), colorectal adenocarcinoma (CRC), and non-small cell lung cancer (NSCLC) [6, 7]. KRAS mutations are highly observed in PDAC, followed by NSCLC and CRC [6, 8]. In the landscape of KRAS-mutated cancer therapeutics, several strategies such as small molecule inhibitors (targets upstream & downstream effectors, direct KRAS and its regulators, cell cycle regulators); siRNA candidates; cancer vaccines; PROteolysis TArgeting Chimers (PROTACs); Antibody–Drug conjugates (ADCs); and Immunotherapies are being developed and studied in preclinical and clinical models [9–12]. However, Sotorasib (AMG 510), and Adagrasib (MRTX849) were the only FDA-approved drugs available for the treatment of KRAS^G12C^ mutated NSCLC [13, 14].

Cancer immunotherapy is a promising strategy to mobilize the patient’s immune system towards tumor cells to elucidate antitumor immune response activation [15, 16]. Monoclonal antibodies (mAbs), immune checkpoint inhibitors (ICI), cytokine therapy, cancer vaccines, and CAR-T cell therapy are the commonly available immunotherapies that could be harnessed to combat KRAS mutated cancers [15, 17, 18]. Amongst, the Chimeric antigen receptor (CAR) T-cell therapy has emerged as an effective immunotherapy strategy that involves in the engineering of T-cells to express the specific Chimeric antigen receptor (CAR) protein that specifically targets the tumor antigens (Neotantigens) and mediates T-cell responses [19, 20]. ABECMA® (idecabtagene vicleucel), BREYANZI® (lisocabtagene maraleucel), CARVYKTI™ (ciltacabtagene autoleucel), KYMRIAH™ (tisagenlecleucel), TECARTUS™ (brexucabtagene autoleucel), and YESCARTA™ (axicabtagene ciloleucel) are some of the FDA-approved CAR T-cell therapies to treat various types of hematological malignancies [21, 22]. Although currently it is limited by its therapeutic landscape for solid cancers, several studies are being carried out at preclinical and clinical levels [23, 24]. In the context of KRAS-mutated PDAC, 75 studies (as of June 13, 2024) are currently studied for the development of CAR T-cell therapy at various clinical phase levels as shown in Supplementary Table S1. Cluster of differentiation 24 (CD24), Prostate stem cell antigen (PSCA), Carcinoembryonic Antigen (CEA), Mucin-1 (MUC-1), Mesothelin (MSLN), Fibroblast activation protein-α (FAP), and human epidermal growth factor receptor 2 (Her-2) are some of the commonly identified target antigens for the development of CAR T-cell therapy against PDAC [25]. Notably, the MSLN (80–85%) and CEA (< 70%) were reported to be highly overexpressed in PDAC [26–29]. Also, when compared to other antigens, several studies are being carried out for targeting MSLN and CEA in PDAC in clinical trials as shown in Supplementary Table S1. Dual targeting CAR-T is an efficient strategy that simultaneously recognizes two different target neoantigens and it is reported to prevent relapse due to antigen downregulation or loss, and has the potency to overcome cancer resistance [30, 31]. However, to the best of our knowledge, there were no studies reported in clinical trials for the development of dual targeting (MSLN and CEA) CAR protein against KRAS-mutated PDAC.

Meanwhile, unlike other therapeutics like small molecule inhibitors, siRNA candidates & vaccine construct, CAR-T development is limited by their bioinformatics approaches to design potent and effective therapeutics. Thus considering this all, in the present study we have employed various bioinformatics approaches such as functional analysis (antigenicity, allergenicity, antigen binding sites & signalling cascades), qualitative analysis (physicochemical, prediction, refinement & validation of 2D and 3D structures), molecular docking, and In silico cloning to design and develop the novel CAR protein to be used in engineering the T-cell to elucidate the antitumor response against KRAS-mutated pancreatic ductal adenocarcinoma.

## Methods

### Data retrieval and CAR design

The FASTA sequences of each subunit of the chimeric antigen receptor (CAR) protein were retrieved from NCBI (National Center for Biotechnology Information), UniProt, and Protein Data Bank (PDB) database respectively [32, 33]. The single-chain variable fragment (scFv) of the mesothelin (MSLN) (PDB ID: 8CZ8_B), scFv of Carcinoma embryonic antigen (CEA) (GenBank ID: AHI45157.1) were used as extracellular domain, which was connected by a perfect linker (GGGGS)_3_. The hinge and transmembrane region was derived from CD8α (UniProt ID: P01732), the proliferation domain was derived from CD28 (UniProt ID: P10747), the survival & cytotoxic domain was derived from CD137 (UniProt ID: Q07011), and the T-cell activation domain was derived from CD3ζ (UniProt ID: P20963), and used in the of the CAR construct sequentially respectively as shown in Fig. 2. Also, the FASTA sequences of cancer cell associated antigens, Mesothelin (UniProt ID: Q13421) and Carcinoembryonic antigen (UniProt ID: P06731) were retrieved respectively.

### Evaluation of physicochemical, antigenicity, and allergenicity properties

The physicochemical properties of the CAR construct were predicted using the ProtParam web server (https://web.expasy.org/protparam/) [34]. Parameters such as the total number of amino acids, molecular formula & weight, theoretical PI, total number of negatively & positively charged residues, and the grand average of hydropathicity (GRAVY) were predicted. The antigenicity of the CAR construct was predicted using VaxiJen v2.0 (https://www.ddg-pharmfac.net/vaxijen/VaxiJen/VaxiJen.html) [35] and SVMTrip (http://sysbio.unl.edu/SVMTriP/) [36] web servers respectively. Also, the allergenicity of the CAR construct was predicted using Algpred (http://crdd.osdd.net/raghava/algpred/) [37] and AllerTOP v.2 (https://www.ddg-pharmfac.net/AllerTOP/) [38] web servers respectively.

### Prediction of 2D and 3D structures

The 2D structure of the designed CAR was predicted by the PDBsum database (http://www.ebi.ac.uk/thornton-srv/databases/pdbsum/Generate.html) [39]. All the secondary structures such as alpha-helix, beta-strand, and the motifs present in the CAR were predicted. Then the 3D structure of the designed CAR was predicted by the I-TASSER web server (https://zhanggroup.org/I-TASSER/) [40] and further refined using the GalaxyRefine web server (https://galaxy.seoklab.org/cgi-bin/submit.cgi?type=REFINE) [41]. Also, it was further validated by Ramachandran plot using PROCHECK (https://www.ebi.ac.uk/thornton-srv/software/PROCHECK/index.html) [42], and by Z-score using ProSA-web web servers respectively (https://prosa.services.came.sbg.ac.at/prosa.php) [43].

### Prediction of antigen-binding sites

The antigen binding sites of the designed CAR were predicted using the Ellipro web server (http://tools.iedb.org/ellipro/) [44]. It scores in the range of 0–1 and the default cut-off was set as 0.5 with a distance of 6 Å, and both linear & discontinuous epitopes were predicted. The epitopes scored above are considered as the paratope sequences and those below 5 are considered non-paratope sequences.

### Molecular docking

The binding affinities between the designed CAR construct and the antigens (MSLN & CEA) were predicted by molecular docking using the HDOCK web server (http://hdock.phys.hust.edu.cn/) [45]. HDOCK server performs the docking of the protein–protein via a Fast Fourier Transform (FFT)-based hybrid algorithm of template-based modelling and ab initio free docking. Then the docked protein–protein complexes were visualized using PyMol [46]. Then the intermolecular interactions of the CAR-CEA & CAR-MSLN docked complexes were predicted using the PDBsum database [39].

### Prediction of signalling cascades

CARs are synthetically engineered protein constructs that are generally involved in the various signalling cascades involved in the T cell’s function, stimulation, proliferation, and activity. It is very necessary to include a proper domain in the CAR in order to attain maximum therapeutic effect. Thus to ensure whether the designed CAR has the ability to elicit the above mentioned T-cell related functions, it was evaluated by the STRING network by using Cytoscape (https://string-db.org/cgi/input?sessionId=bdNete4Aawu7&input_page_show_search=on) [47].

### Codon optimization and in silico cloning

**Fig. 1:**
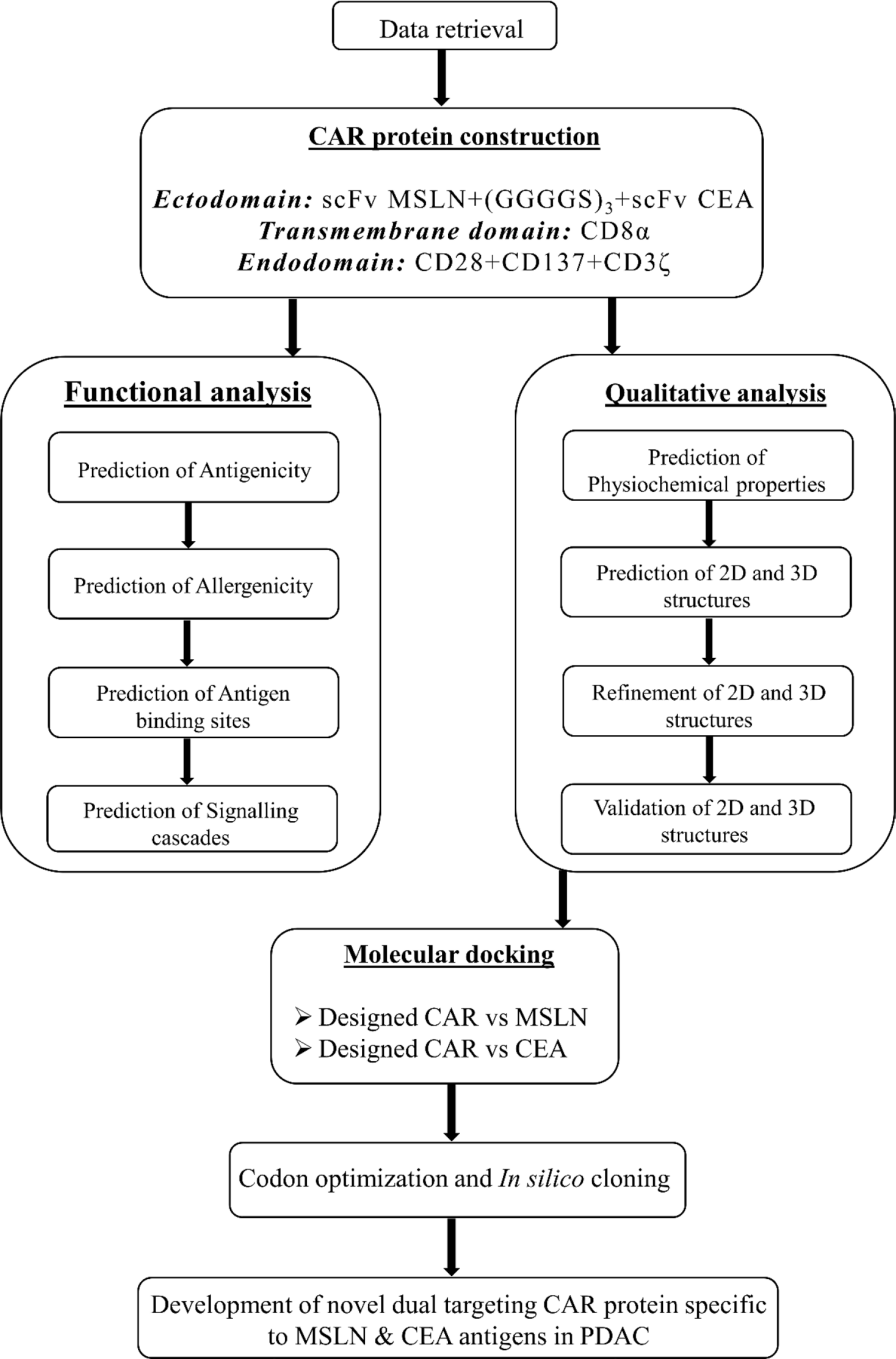
Workflow of the present study

Initially, the protein sequence of the CAR construct was codon optimized (reverse translated and optimized) using Java Codon Adaptation Tool (JCat) by employing Homo sapiens as the expression system (https://www.jcat.de/) [48]. Then the codon-optimized CAR sequence was cloned into the pcDNA3.1/C-his (5432 bp) by flanking kpnl and the EcoRI restriction sites using the SnapGene tool (https://www.snapgene.com/). The complete workflow of the present study was depicted in Fig. 1.

## Results

### Construction of chimeric antigen receptor (CAR)

**Fig. 2:**
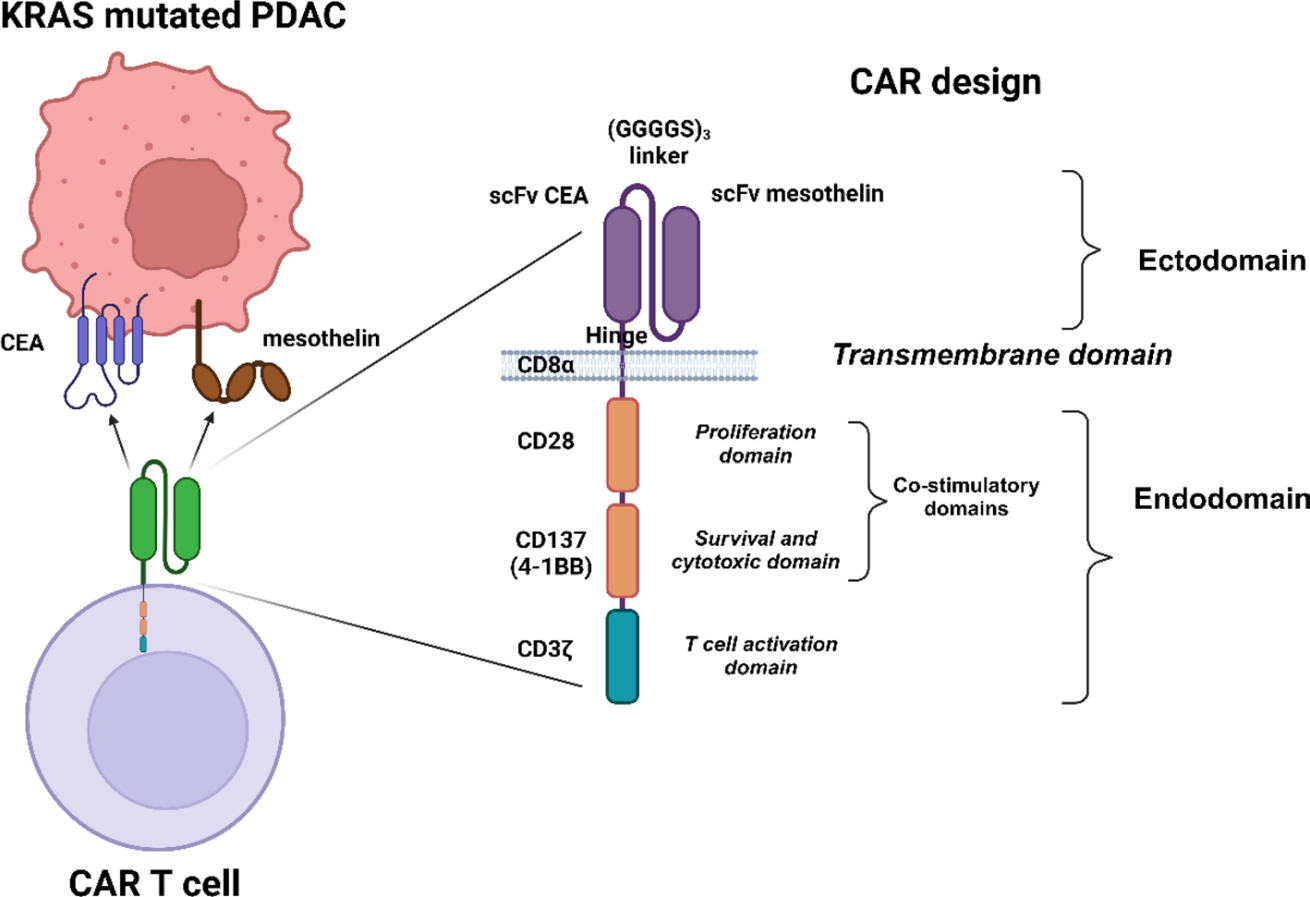
Designing of dual targeting CAR construct against the KRAS-mutated PDAC

All the respective sequences of the proteins used in the CAR design were retrieved from the NCBI, PDB, and UniProt databases respectively. The single-chain variable fragments (VH and VL domains) of the mesothelin (MSLN) and Carcinoma embryonic antigen (CEA) monoclonal antibodies connected by (GGGGS)_3_ linker form the extracellular epitopes of the CAR construct. While CD8α forms the hinge and transmembrane region, CD28 forms the proliferation domain, CD137 forms the survival & cytotoxic domain, and CD3ζ forms the T-cell activation domain of the CAR construct respectively as shown in Fig. 2.

### Physicochemical, antigenicity, and allergenicity of the CAR construct

Initially, the physicochemical properties of the CAR construct such as the total number of amino acids, molecular weight & formula, theoretical pI, total number of positive charge residues (Arg + Lys) & negative charge residues (Asp + Glu), GRAVY, estimated half-life were predicted using the ProtParam web server and tabulated in Table 1. The designed CAR construct shows a half-life period of more than 10 h in the mammalian system. In the CAR construct, it is observed that the Gly (G) is more frequent with 11.3% and His (H) is less frequent with 1.0% respectively as shown in Supplementary Figure S1. Then, the antigenicity of the CAR construct was predicted using VaxiJen v2.0 & SVMTrip webservers which showed an antigenic score to be 0.5551 (Probable ANTIGEN), and probability scores from 1.00 to 0.3 at various regions respectively. Also, the allergenicity was predicted using Algpred and AllerTOP v.2 web servers that showed the CAR construct was non-allergenic.

**Table 1:** Physiochemical properties of the designed CAR

Parameter	Value/range
Number of amino acids	1387
Molecular formula	C_6658_H_10372_N_1854_O_1990_S_72_
Molecular weight	150,539.14 Da
Theoretical pI	9.10
Total number of positive charge residues (Arg + Lys)	144
Total number of negative charge residues (Asp + Glu)	102
GRAVY	− 0.258
Estimated half life	30 h (mammalian reticulocytes, in vitro) > 20 h (yeast, in vivo) > 10 h (Escherichia coli, in vivo)

### Structural refinement and validation of the CAR construct

**Fig. 3:**
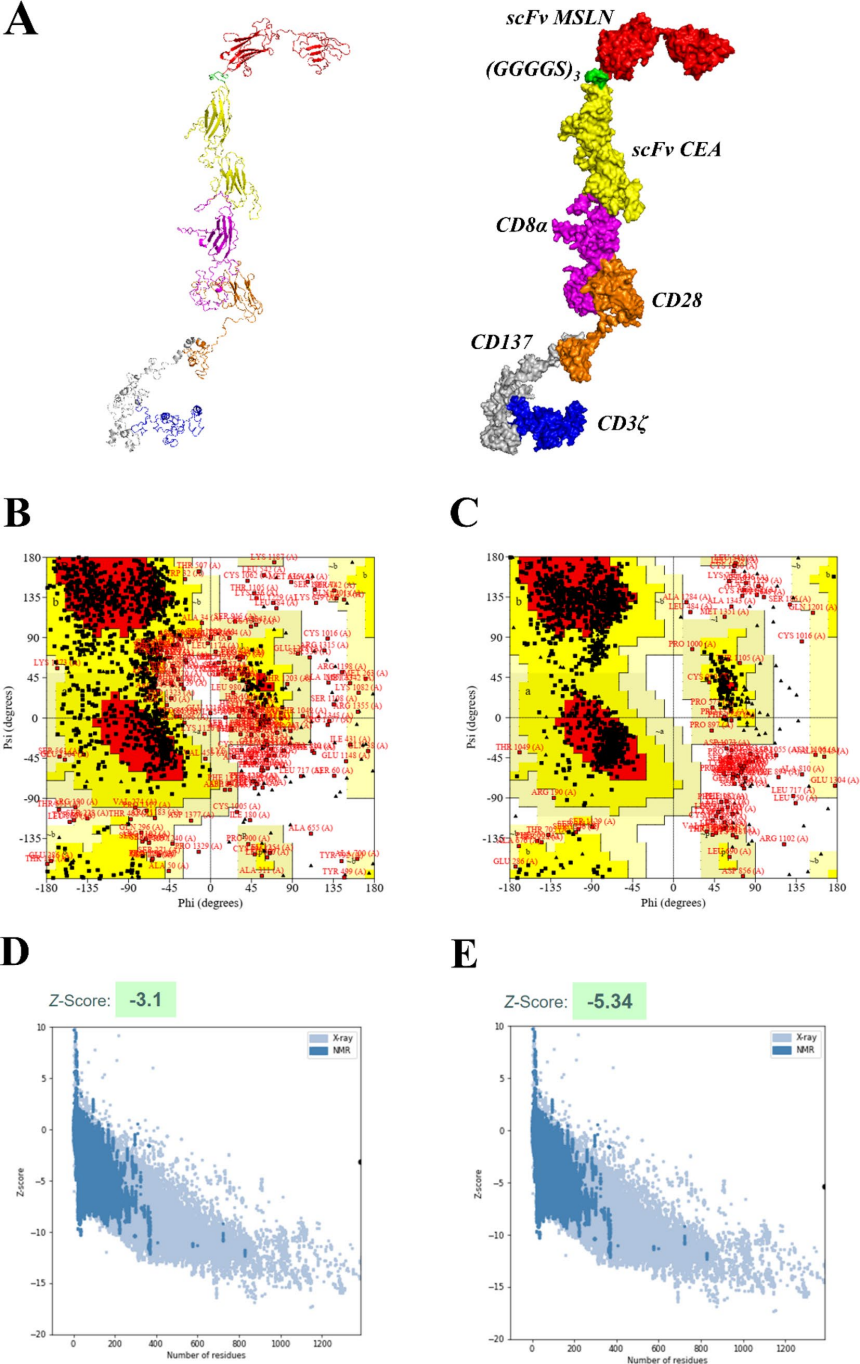
Refined 3D structure of the designed CAR with its domains (**A**). Ramachandran plots of unrefined (**B**) and refined model (**C**), Z-score plots of unrefined (**D**) and refined model (**E**) of CAR were also shown respectively

Initially, the 2D structure of the designed CAR was predicted using the PDBsum database. The CAR construct comprising of 1387 AA was predicted to have 13 sheets, 13 beta hairpins, 2 beta bulges, 43 strands, 25 helices, 3 helix-helix interaction, 350 beta turns, 147 gamma turns, and 1 disulfide as shown in Supplementary Figure S2. Then the 3D structures of the CAR construct, CEA, and MSLN were predicted by the I-TASSER web server, which generated 5 best models (models 1–5) with respective C-values. Usually, the high C-score value represents the high confidence model, and thus the high C-value models were selected. The C-value of the predicted models of CAR are − 3.44, − 0.93, − 3.69, − 3.71, − 3.69; CEA are − 1.11, − 2.57, − 2.35, − 1.30, − 1.81; and MSLN are − 1.76, − 1.90, − 3.68, − 3.55, − 2.04 respectively. From the above, model 2 of CAR, model 1 of CEA, and model 1 of MSLN were taken for further refinement using the GalaxyRefine web server. Similarly, GalaxyRefine predicted the 5 best refined models (model 1–5), and the model 1 of CAR having GDT-HA (0.8344), RMSD (0.760), MolProbity (2.477), Clash score (19.6), Poor rotamers (0.8), Rama favored (83.3); model 1 of CEA having GDT-HA (0.9370), RMSD (0.458), MolProbity (2.540), Clash score (26.5), Poor rotamers (0.5), Rama favored (86.6); and model 1 of MSLN having GDT-HA (0.8980), RMSD (0.552), MolProbity (2.617), Clash score (25.9), Poor rotamers (1.5), Rama favored (88.9) were selected respectively. Also, the unrefined and refined models of CAR, CEA, and MSLN were shown in Supplementary Figure S3. Following this, the refined models were validated by Ramachandran plot using the PDBsum database and Z-score using the ProSAweb web server respectively. From the Ramachandran plot, the most favored regions for unrefined and refined models of designed CAR were calculated to be 46.8% and 72.7% respectively. Also, the Z-score was enhanced from − 3.1 (unrefined) to − 5.34 (refined), indicating that the refined model was perfect as shown in Fig. 3.

### Antigen binding sites in the CAR construct

**Fig. 4:**
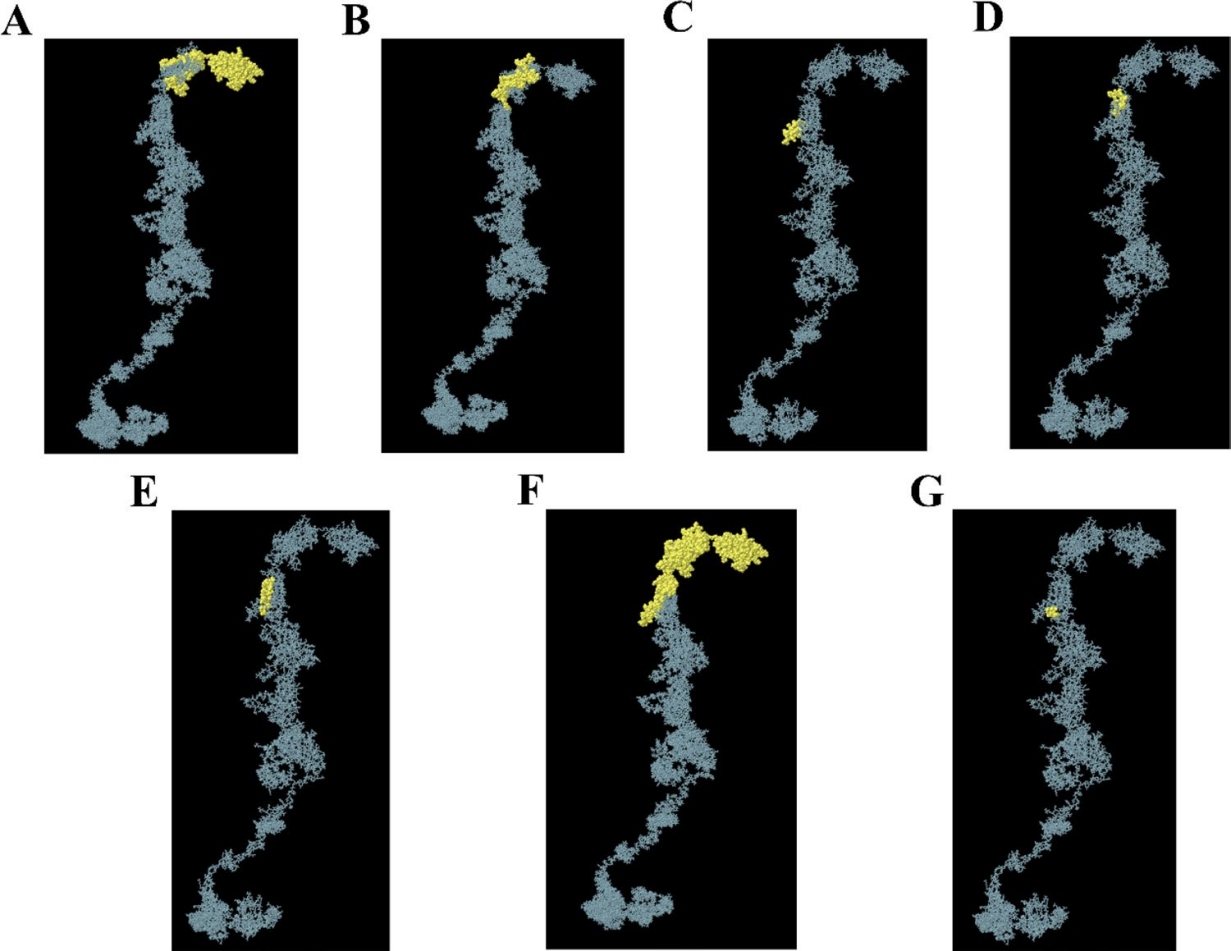
Antigen binding sites of the designed CAR. Continues (linear) epitope binding regions of MSLN (**A**, **B**), Continues (linear) epitope binding regions of CEA (**C**, **E**), and discontinuous epitope binding regions of MSLN (**F**) and CEA (**F**, **G**) were depicted respectively

Though the CAR was generally engineered to bind to specific cancer antigens, it is necessary to analyze the antigen-binding sites of the designed CAR. The antigen (epitopes) binding regions on the constructed CAR (paratopes) were predicted using the Ellipro server, which shows 5 continuous (linear) and 2 discontinuous epitope binding regions as shown in Fig. 4 and Table 2. The cancer antigen binding regions on the CAR were observed to be at the MSLN & CEA domains (extracellular domains) indicating that it could significantly bind to the target antigen of the cancer cell.

**Table 2:** Peptide sequences of Linear and discontinuous epitopes against the designed CAR

Type	Peptide	Number of residues	Score
Linear (continuos)	DIQMTQSPSSLSASVGDRVTITCRSSQGIGSWLAWYQQKPEKAPQSLIYAASSLQSGVPSRFSGSGSGTDFTLTISNLQPEDFATYYCQQYNSYPLTFGGGTKVEIKGGGSGGGSGGGSGGGSEVQLLESGGGLVQPGGSLRLSCAASGLTFRSYAMTWVRQAPGKGLEWVSGISVSGGITY	182	0.797
	DSVKGRFTISRDNSKNTLYLQMNSLRAEDTAVYYCAKRGAAVGSFDYWGQGTLVTVSSGGGGSGGGGSGGGGSGSD	76	0.688
	GKSPQLLVYSAANLADSVPSR	21	0.646
	RTSENIYSNLAW	12	0.578
	SGSGSGTQFSLKINS	15	0.578
Discontinuous	D1, I2, Q3, M4, T5, Q6, S7, P8, S9, S10, L11, S12, A13, S14, V15, G16, D17, R18, V19, T20, I21, T22, C23, R24, S25, S26, Q27, G28, I29, G30, S31, W32, L33, A34, W35, Y36, Q37, Q38, K39, P40, E41, K42, A43, P44, Q45, S46, L47, I48, Y49, A50, A51, S52, S53, L54, Q55, S56, G57, V58, P59, S60, R61, F62, S63, G64, S65, G66, S67, G68, T69, D70, F71, T72, L73, T74, I75, S76, N77, L78, Q79, P80, E81, D82, F83, A84, T85, Y86, Y87, C88, Q89, Q90, Y91, N92, S93, Y94, P95, L96, T97, F98, G99, G100, G101, T102, K103, V104, E105, I106, K107, G108, G109, G110, S111, G112, G113, G114, S115, G116, G117, G118, S119, G120, G121, G122, S123, E124, V125, Q126, L127, L128, E129, S130, G131, G132, G133, L134, V135, Q136, P137, G138, G139, S140, L141, R142, L143, S144, C145, A146, A147, S148, G149, L150, T151, F152, R153, S154, Y155, A156, M157, T158, W159, V160, Q162, A163, P164, G165, K166, G167, L168, E169, W170, V171, S172, G173, I174, S175, V176, S177, G178, G179, I180, T181, Y182, Y183, A184, D185, S186, V187, K188, G189, R190, F191, T192, I193, S194, R195, D196, N197, S198, K199, N200, T201, L202, Y203, L204, Q205, M206, N207, S208, L209, R210, A211, E212, D213, T214, A215, V216, Y217, Y218, C219, A220, K221, R222, G223, A224, A225, V226, G227, S228, F229, D230, Y231, W232, G233, Q234, G235, T236, L237, V238, T239, V240, S241, S242, G243, G244, G245, G246, S247, G248, G249, G250, G251, S252, G253, G254, G255, G256, S257, G258, S259, D260, I261, Q262, T264, Q265, R283, T284, S285, E286, N287, I288, Y289, S290, N291, L292, A293, W294, G300, K301, S302, P303, Q304, L305, L306, V307, Y308, S309, A310, A311, N312, L313, A314, D315, S316, V317, P318, S319, R320, F321, S322, G323, S324, G325, S326, G327, T328, Q329, F330, S331, K333, F350, Y351, G352, T353, P354, P355	314	0.733
	E276, T277, N335, S336	4	0.502

### Molecular interactions of the CAR construct with target antigens

**Fig. 5:**
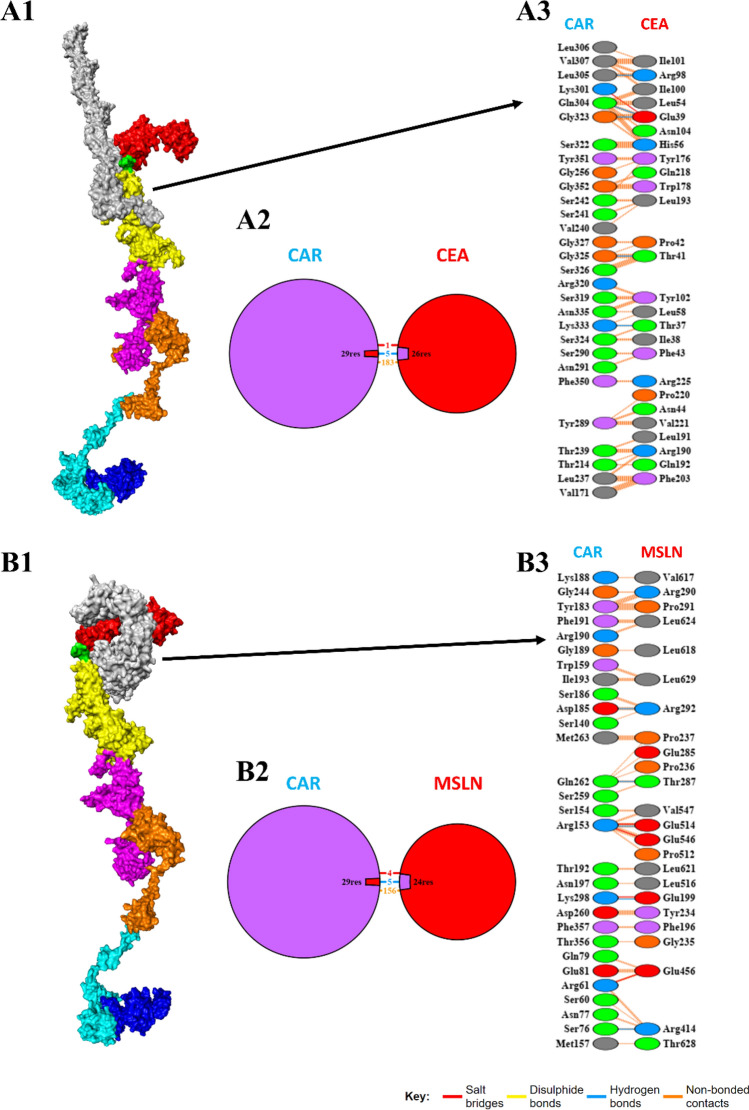
Molecular interactions of designed CAR with CEA and MSLN. Surface view interaction (**A1**), total number of interactions (**A2**), & interacting residues (**A3**) of CAR-CEA docked complexes, and surface view interaction (**B1**), total number of interactions (**B2**), & interacting residues (**B3**) of CAR-MSLN docked complexes. In the surface view, MSLN scFv was shown in red, (GGGGS)_3_ was shown in green, CEA scFv was shown in yellow, CD8α was shown in magenta, CD28 was shown in orange, CD137 was shown in cyan, CD3ζ was shown in blue, and the CEA (**A1**) and MSLN (**B1**) were shown in gray respectively

The molecular docking of the CAR construct with the MSLN & CEA antigens was performed using the HDOCK web server and their binding affinities were predicted. The binding energies of the CAR-MSLN and CAR-CEA docked complexes were predicted to be − 308.11 kcal/mol and − 278.3 kcal/mol respectively. The interactions of the protein–protein complexes were predicted using PDBsum and visualized. The surface view interaction, total number of interactions, and interacting residues of the CAR-MSLN and CAR-CEA docked complexes were shown in Fig. 5. MSLN specifically binds with the scFv MSLN domain in the designed CAR, and shows 1593 Å^2^ inter surface area and 24 interacting residues while the CAR showed 1422 Å^2^ inter surface area and 29 interacting residues respectively. CAR-MSLN docked complex showed 4 salt bridges, 5 H-bonds, and 156 non-bonded contacts. Likewise, CEA specifically binds with the scFv CEA domain in the designed CAR, and shows 1369 Å^2^ inter surface area and 26 interacting residues while the CAR showed 1337 Å^2^ inter surface area and 29 interacting residues respectively. CAR-CEA docked complex showed 1 salt bridge, 5 H-bonds, and 183 non-bonded contacts as shown in Fig. 5. Molecular docking studies revealed that the designed CAR specifically binds with the target antigens MSLN & CEA with high affinity, and could be able to form several intermolecular interactions.

### Signalling cascades of the CAR construct

**Fig. 6:**
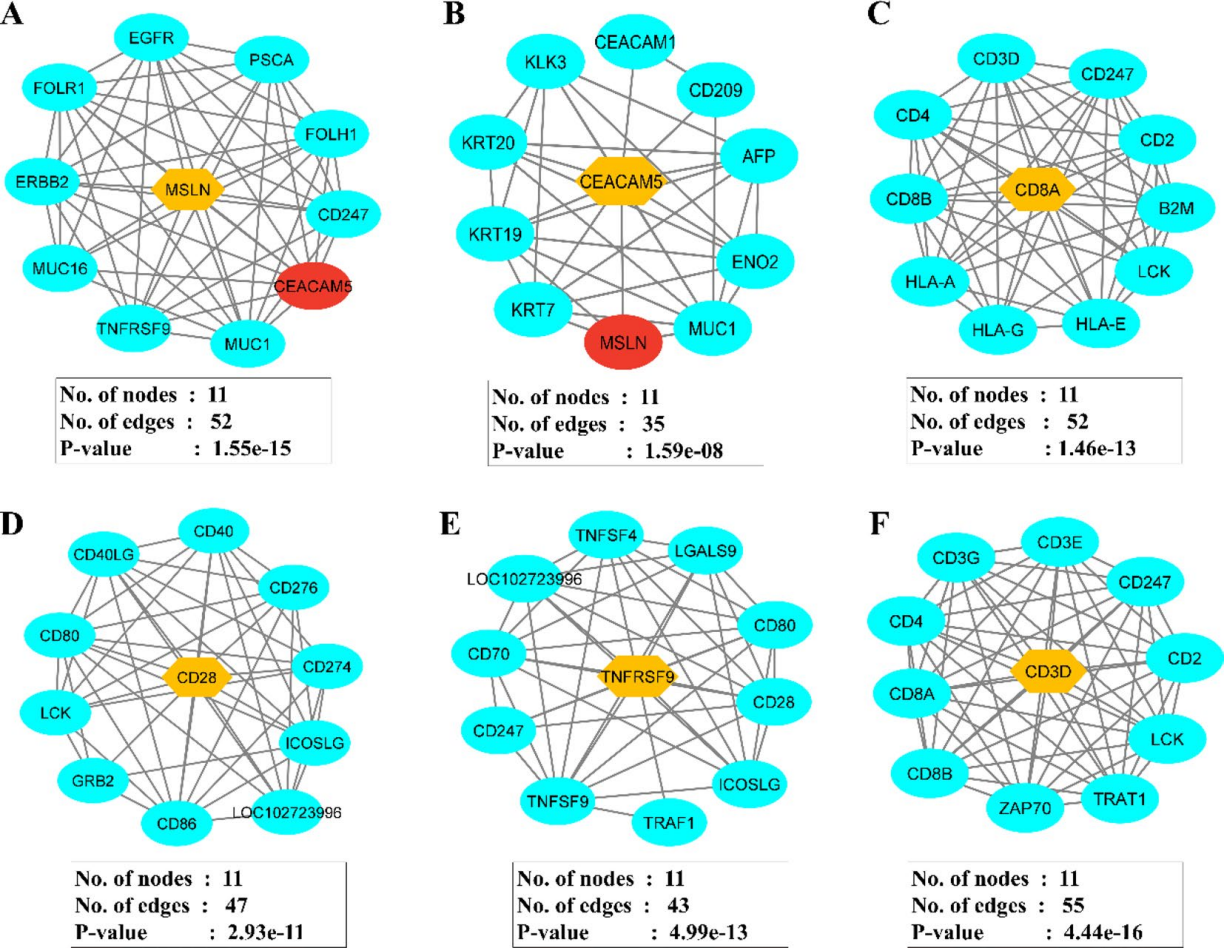
Protein–protein interaction (PPI) network of MSLN (**A**), CEA (**B**), CD8A (**C**), CD28 (**D**), CD137 (**E**), CD3D (**F**) respectively. Also, the number of nodes, edges and gene ontology enrichment p-values were of each PPI network were provided

The T-cell signalling related responses of all the domains of the designed CAR protein and cancer antigens (MSLN & CEA) were predicted by the protein–protein interaction (PPI) network using the STRING and Cytoscape. The PPI network of MSLN, CEA, CD8A (CD8α), CD28, CD137 (TNFRSF9), & CD3D (CD3ζ) along with their respective number of nodes, number of edges, and p-values were predicted as shown in Fig. 6. Usually, the network nodes represent proteins and the edges represent protein–protein associations. The gene ontology (GO) functional enrichment such as Cellular component (CC), Subcellular localization, Tissue expression, and Disease-gene associations of MSLN & CEA were analyzed and tabulated in Supplementary Tables S2, S3 respectively. And, the GO enrichment such as biological process (BP), molecular function (MF), cellular compartment (CC), subcellular localization was analyzed for CD8A (CD8α), CD28, CD137 (TNFRSF9), and CD3D (CD3ζ) domains of the CAR protein. And tabulated in Supplementary Tables S4–S7 respectively.

From the data, we observed that both MSLN & CEA antigens have enrichment terms with respect to the extracellular-related functions and are implicated in several cancers including PDAC and CRC in which the KRAS is highly mutated. Notably, the combined confidence of the function interaction of MSLN & CEA was observed to be 0.791 (high), and the has significant co-expression behavior (p = 0.042). This observation strongly indicates the significance of these antigen’s selection to develop dual antigen-specific CAR. While all the domains of the CAR have enrichment terms with respect to the T-cell signalling responses. CD8α domain was observed to have enrichment such as positive regulation of T cell activation, cell surface receptor signaling pathway, positive regulation of CD8-positive T cell proliferation, peptide antigen binding, cell surface, external side of plasma membrane, and plasma membrane protein complex respectively. CD28 domain was observed to have enrichment such as positive regulation of T cell activation & proliferation, regulation of T cell differentiation, positive regulation of cytokine production, co-receptor activity, integral component of membrane, immunological synapse, and plasma membrane signaling receptor complex respectively. CD137/TNFRSF9 domain was observed to have enrichment such as positive regulation of immune system process, positive regulation of immune response, T cell co-stimulation, positive regulation of activated T cell proliferation, regulation of cytokine production, positive regulation of interleukin-10 production, tumor necrosis factor receptor binding, a protein complex involved in cell adhesion, and T cell receptor complex respectively. CD3D domain was observed to have enrichment such as positive regulation of T cell activation, positive regulation of T cell receptor signaling pathway, positive regulation of intracellular signal transduction, positive regulation of interleukin-2 production, T cell receptor binding, co-receptor activity, protein tyrosine kinase binding, immunological synapse, cytoplasmic side of plasma membrane, and T cell receptor complex respectively. Collectively, the domains of the designed CAR protein have gene ontology enrichment terms related to the T-cell signalling as shown in Fig. 2.

### Codon-optimized CAR construct is stable in the mammalian expression system

**Fig. 7:**
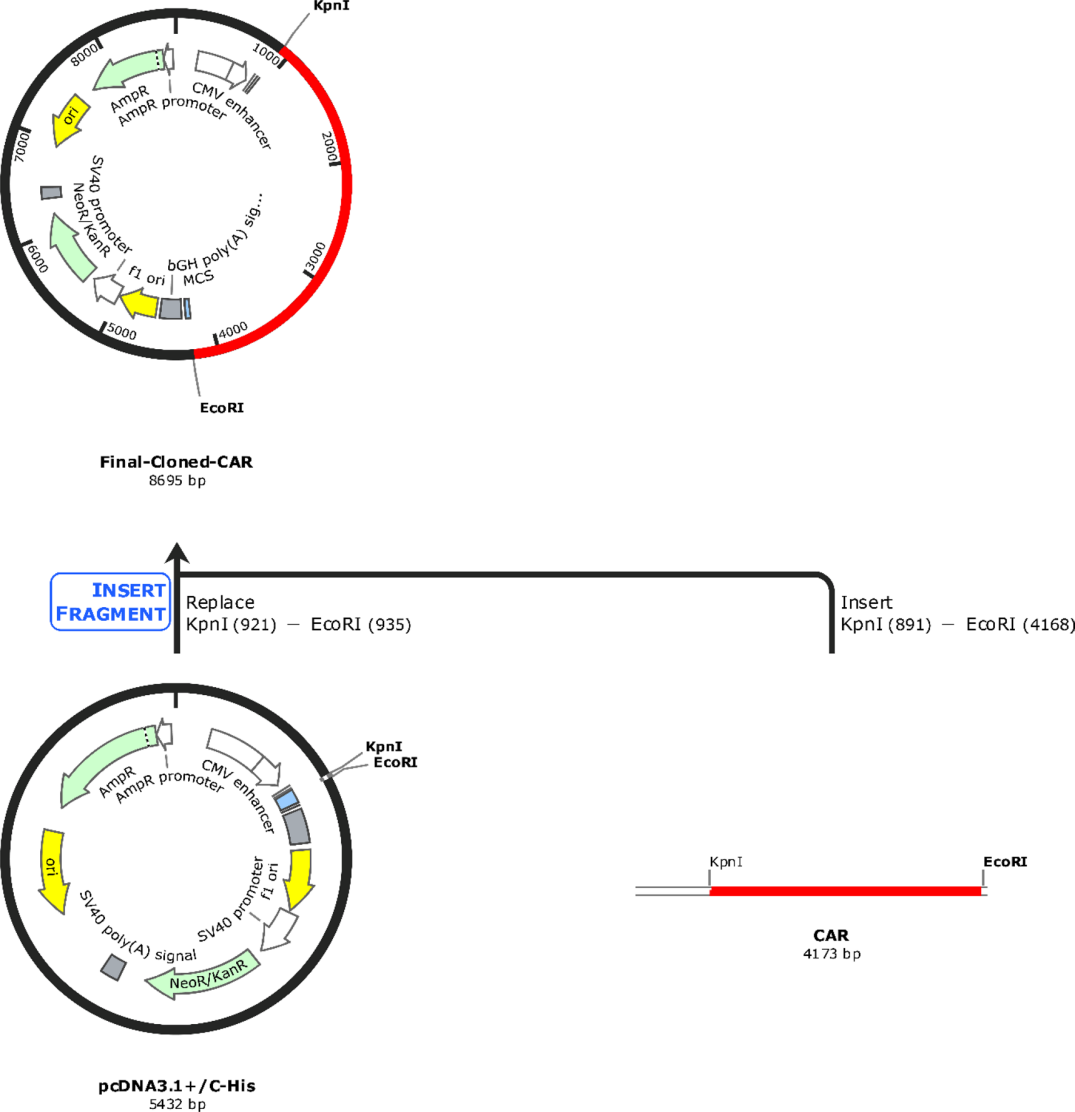
In silico cloning of the codon-optimized CAR protein sequence (4173 bp) shown in red was cloned in the pcDNA3.1+/C-His expression vector (5432 bp) shown in black between restriction sites Kpnl (GGTACC) and EcoR1 (GAATTC), and final cloned vaccine construct was shown (8695 bp)

The CAR construct was reverse-translated and codon-optimized in order to clone it in the human (homo sapiens) expression system using the Jcat tool. The DNA sequence of 4161 nucleotides was obtained with a CAI-value of 0.95 and GC% of 69.28% respectively, and the GC% of the human expression system was predicted to be 40.89%. In addition, the restriction sites of Kpnl (GGTACC) and EcoR1 (GAATTC) were added at the N-terminal and C-terminal of the CAR protein sequence. Then the codon-optimized CAR (4173 nucleotides) was successfully cloned in the pcDNA3.1+/C-His vector (5432 bp) at the restriction sites of Kpnl (GGTACC) and EcoR1 (GAATTC) respectively using the SnapGene tool. The final CAR cloned product (8695 bp) was shown in Fig. 7.

## Discussion

The hotspot mutations (G12, G13, and Q61) of KRAS, overcomes the GTPase activity and promote the proliferation & progression of various cancers including pancreatic ductal adenocarcinoma (PDAC), colorectal adenocarcinoma (CRC), and non-small cell lung cancer (NSCLC) respectively [49, 50]. Although therapeutic strategies such as small molecule inhibitors (targets upstream & downstream effectors, direct KRAS and its regulators, cell cycle regulators); siRNA candidates; cancer vaccines; PROteolysis TArgeting Chimers (PROTACs); Antibody–Drug conjugates (ADCs); and Immunotherapies are being developed [9, 12]. Although, several therapeutics are being studied, there are some concerns and limitations that hinder the mutant KRAS therapeutics development. The highly mutated KRAS codons (KRAS G12C and KRAS G12D) seemed to be undruggable for the past 40 years due to their smooth surface and lack of suitable binding pockets [51]. Also, it has the highly competitive GTP-binding pocket (where the P-loop containing the 12th residue is present), to which the GTP’s are able to bind with picomolar (pM) affinity and also available in nanomolar (nM) levels in cells [50]. Finally, it became druggable with the discovery and FDA-approval (accelerated approval) of Sotorasib (AMG 510), and Adagrasib (MRTX849) for the treatment of KRAS G12C (off-state) mutated NSCLC [13, 14]. Sotorasib and Adagrasib forms the irreversible covalent bond with the reactive cysteine, and this clearly indicates that the KRAS G12C inhibitors can’t be extended to the KRAS G12D mutant due to the lack of reactive cysteine [52]. However, after this FDA-approval several pharma industries are developing both On-state and Off-state KRAS G12C and KRAS G12D mutant inhibitors. In addition, other therapeutics like immunotherapies and targeted protein degraders receive more attraction due to their significant therapeutic potential against the mutant KRAS.

CAR T-cell therapy has emerged as a promising immunotherapy strategy that involves in the engineering of the patient’s own T-cell with CAR protein that specifically targets the antigens present in the cancer cells [53, 54]. Also, to date there are 6 CAR T-cell therapies have been approved by the FDA for the treatment of various hematological malignancies, and several studies are being carried out to develop potent CAR T-cell therapies for solid cancers [21, 22, 53]. In the context of PDAC, currently 75 clinical trials are being studied for the development of CAR T-cell therapy at various clinical phase levels as shown in Supplementary. Amongst the identified cancer antigens of KRAS-mutated PDAC, CEA and MSLN were reported to be overexpressed and promoted tumor progression PDAC [26–28]. Dual targeting CAR-T is an efficient strategy to promote CAR-T efficiency [31], and we observed no reports on the development of dual targeting CAR protein targeting towards MSLN and CEA which are overexpressed in KRAS-mutated PDAC. Recently, the early trial results from 6 patients with recurrent glioblastoma showcased the significant possibility of developing dual-targeting CAR-T for solid cancers [55]. They have the studied potency of the bivalent chimeric antigen receptor (CAR) T-cells which targets both epidermal growth factor receptor (EGFR) and interleukin-13 receptor alpha 2 (IL13Rα2) in recurrent glioblastoma and found a significant tumor size reduction and cytokine release indicating it’s promising activity in early efficacy. In addition, the dual targeting CAR-T therapy has also shown significant therapeutic effects in various cancers such as B-cell acute Lymphoblastic Leukemia (B-ALL) [56]; diffuse large B-cell lymphoma (DLBCL) [31]; acute myeloid leukemia (AML) [57]; and multiple myeloma (MM) [58] respectively. Meanwhile, there is a lack of bioinformatics approaches for the design and development of CAR protein, and thus from all the above understandings, we have planned to develop dual targeting (MSLN & CEA) CAR protein to develop CAR T-cell against the KRAS-mutated PDAC by employing various bioinformatics approaches such as functional analysis (antigenicity, allergenicity, antigen binding sites & signalling cascades), qualitative analysis (physicochemical, prediction, refinement & validation of 2D and 3D structures), molecular docking, and in silico cloning.

Mesothelin (MSLN) overexpression in PDAC promotes cancer progression and aggressiveness by various regulating various molecular events including, the elevation of Cyclin E levels via STAT3 activation, inhibition of pro-apoptotic proteins (Bim & Bax), induction of anti-apoptotic proteins (Bcl-2 & Bcl-xl) respectively [59, 60]. Another study highlighted that MSLN regulates apoptosis in PDAC via both p53-dependent and p53-independent pathways [61]. While, the carcinoembryonic antigen (CEA) present in the patient’s serum is a commonly used biomarker for PDAC, and its overexpression is associated with tumor progression and metastasis [62]. A retrospective study employing 128 PDAC patients reported the significance of CEA as a prognostic marker of PDAC [63]. Of note, CEA is also used as a chemo-resistant marker [64] and as a radio-resistant marker [65] in colorectal cancer. Given the importance of MSLN and CEA, we have designed our dual-targeting CAR protein specific to these antigens. The designed CAR domains include the ectodomain [scFv MSLN connected to scFv CAE via (GGGGS)_3_]; hinge and transmembrane region [CD8α]; and endodomain [CD28, CD137, CD3ζ] respectively as shown in Fig. 2. CD8α acts as a transmembrane region protein that holds the CAR domain in both cytoplasmic and exoplasmic regions; CD28 is the proliferation domain of the CAR that promotes the phosphorylation of CD3ζ domain; CD137 is the survival & cytotoxic domain that regulates cytokine production; and CD3ζ is the T-cell activation domain that regulated the T-cell mediated responses [66, 67]. The designed CAR protein was expected to specifically bind with both MSLN & CEA antigens of KRAS-mutated PDAC cells and further promotes the T-cell mediated responses (once engineered on the surface of CAR T-cell). While, Zhang & colleagues have successfully developed a dual targeting CAR-T specific towards MSLN & CEA towards solid cancers and observed tumor reduction properties [68].

The structural and functional properties of the CAR protein are essential to bind to target cancer cells and to promote T-cell mediated responses [69]. In our study, we evaluated the physiochemical properties and observed that the 150,539.14 Da molecular weight CAR protein construct contains a total of 1387 amino acids, in which 144 and 102 are positive (Arg + Lys) and negative (Asp + Glu) charged residues. Additionally, the designed CAR construct showed a half-life period of 1.1 h in the mammalian system as shown in Table 1. Notably, the designed CAR showed antigenicity properties and was predicted to be non-allergen. The CAR structure plays a vital role in exhibiting the therapeutic response upon antigen binding, and thus it is important that each CAR domain is structurally functional to elucidate the antitumor response [70]. The 2D structure of the designed CAR was predicted and observed to have 13 sheets, 13 beta hairpins, 2 beta bulges, 43 strands, 25 helices, 3 helix-helix interaction, 350 beta turns, 147 gamma turns, and 1 disulfide as shown in Supplementary Figure S2. Then the 3D structure of the CAR was predicted, refined, and validated by Ramachandran plot and Z-score respectively. Following this, the perfectly refined model was taken for docking studies, in which the CAR was docked against MSLN and CEA antigens. We observed that the MSLN & CEA antigens were specifically bound with the scFv MSLN & scFv CEA domains of CAR with the binding energies of − 308.11 kcal/mol and − 278.3 kcal/mol respectively. The CAR-MSLN docked complex showed 4 salt bridges, 5 H-bonds, and 156 non-bonded contacts, and the CAR-CEA docked complex showed 1 salt bridge, 5 H-bonds, and 183 non-bonded contacts as shown in Fig. 5. Molecular docking studies revealed that the designed CAR specifically binds with the target antigens MSLN & CEA with high affinity, and could be able to form several intermolecular interactions. In support of this, the common antigen binding sites in CAR were also observed to be at scFv MSLN & scFv CEA domains as shown in Fig. 5.

**Fig. 8:**
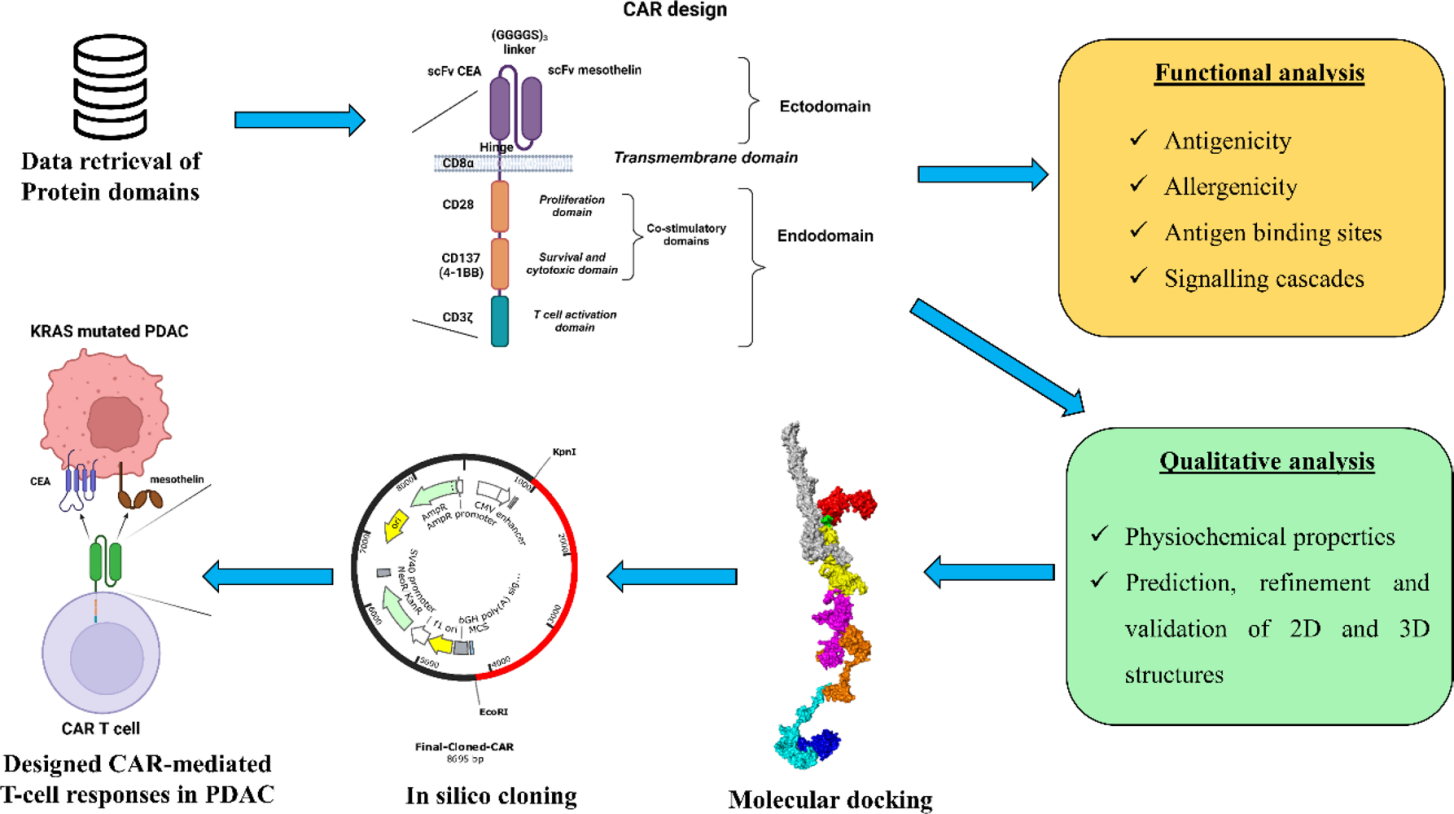
Overall summary of the present study. The designed dual targeting CAR has the potential to bind to the CEA and MSLN antigens of the KRAS mutated PDAC, and could exerts T cell mediated cancer cell death

CD8α transmembrane domain of the CAR was reported to elevate the TNFα and (IFN)-γ levels and to reduce the activation-induced cell death [71]; while CD28 & CD137 are established co-stimulatory domains that promote the CAR T-Cell effector function [72]; CD3ζ interacts with endogenous T-cell receptor to mediate T-cell activation and responses [66]. A study reported that CD33-specific CAR-T that employed the ectodomain comprising of CD28-CD137- CD3ζ induced a strong potent antitumor response and significant levels of cytokines [73]. Similarly, we have also employed the same ectodomains in the designed CAR as shown in Fig. 2. We have also predicted the protein–protein interaction (PPI) networks of each domain of the CAR and found that MSLN & CEA are cell adhesion molecules and are involved in the extracellular related functions in PDAC in which KRAS mutations are highly observed. Notably, the combined confidence of the function interaction of MSLN & CEA was observed to be 0.791 (high), and the has significant co-expression behavior (p = 0.042), indicating the high chances of co-expression in PDAC cells. Moreover, all the domains have enrichment terms with respect to the T-cell signalling responses, PDAC, cytokine production, and co-receptor activities as shown in Fig. 2. Furthermore, the CAR construct was reverse-translated and codon-optimized in order to clone it in the human (homo sapiens) expression system [74]. The restriction sites of Kpnl (GGTACC) and EcoR1 (GAATTC) were added at the N-terminal and C-terminal of the CAR protein sequence, and successfully cloned in the pcDNA3.1+/C-His vector (5432 bp) at the restriction sites of Kpnl (GGTACC) and EcoR1 (GAATTC) and the final CAR cloned product (8695 bp) was shown in Fig. 7. We have successfully designed and developed the CAR protein construct specific towards MSLN & CEA overexpressed KRAS mutated PDAC using versatile bioinformatics approaches as shown in the Fig. 8. However, when CAR T is developed for solid cancers, certain limitations should be considered such as inappropriate targets, T cell exhaustion, resistance in case of single antigen targeting, and expandability to other cancer types respectively [70].

## Conclusion and future perspectives

Mutant KRAS-induced tumorigenesis is prevalent in PDAC, NSCLC, & CRC and there is a huge need for potent therapeutics. CAR T-cell therapy has emerged as a promising immunotherapy strategy that specifically targets antigens (Neoantigens) in cancer cells. In this study, we have employed various bioinformatics approaches and designed a dual targeting CAR protein construct against MSLN & CEA that are overexpressed in KRAS mutated PDAC. Our study showcased that the designed CAR was structurally stable, non-allergenic, and specifically binds with the target antigens with significant binding affinities. Also, the protein–protein interaction network indicated the possible T-cell mediated antitumor responses of each domain of the CAR respectively. Conclusively, we have designed and developed a dual targeting (MSLN & CEA) CAR protein using computational approaches, and we also suggest extending this study by engineering this designed CAR in T-cell and to evaluate their therapeutic efficiency in in vitro and in vivo studies in the near future. While, other therapeutics such as inhibitors (docking & simulation), siRNA (U,R,A design rules) & vaccines (reverse vaccinology & immunoinformatics) have sufficient bioinformatics pipelines, CAR T-cell therapy development is limited by their bioinformatics approaches. Thus, we also recommend the researchers to employ these kinds of bioinformatics pipelines to develop potent CAR that could be engineered on T-cells to further elucidate the antitumor response.

## Supplementary Information

Supplementary Material 1.

## Data Availability

All data generated or analysed during this study are included in this published article or supplementary information.
