# Supplementary material for: Design and development of dual targeting CAR protein for the development of CAR T-cell therapy against KRAS mutated pancreatic ductal adenocarcinoma using computational approaches
Source: Discov Oncol. 2024 Oct 25;15:592. doi: 10.1007/s12672-024-01455-6 (PMC11511808; doi:10.1007/s12672-024-01455-6)
Supplement: Supplementary file 1 — Supplementary Material 1. [file 12672_2024_1455_MOESM1_ESM.docx]

**Design and Development of Dual Targeting CAR protein for the Development of CAR T- Cell Therapy Against KRAS Mutated Pancreatic Ductal Adenocarcinoma Using Computational Approaches**

Prasanna Srinivasan Ramalingam^1^, Premkumar T^2^, Vino Sundararajan^2^, Md Sadique Hussain^3^, Sivakumar Arumugam^1, *^

^1^Protein Engineering lab, School of Biosciences and Technology, Vellore Institute of Technology, Vellore, India.

^2^Integrative Multiomics Lab, School of Bio-Sciences & Technology, Vellore Institute of Technology, Vellore, Tamil Nadu, India.

^3^Uttaranchal Institute of Pharmaceutical Sciences, Uttaranchal University, Dehradun, Uttarakhand 248007, India.

*Corresponding author: [siva_kumar.a@vit.ac.in](mailto:siva_kumar.a@vit.ac.in)

**ORCID ID’s**

Prasanna Srinivasan Ramalingam: <https://orcid.org/0000-0002-8281-2779>

Premkumar T: <https://orcid.org/0009-0006-0651-8259>

Vino Sundararajan: <https://orcid.org/0000-0002-0015-8460>

Md Sadique Hussain: <https://orcid.org/0000-0002-3554-1750>

Sivakumar Arumugam: <https://orcid.org/0000-0001-8834-8834>


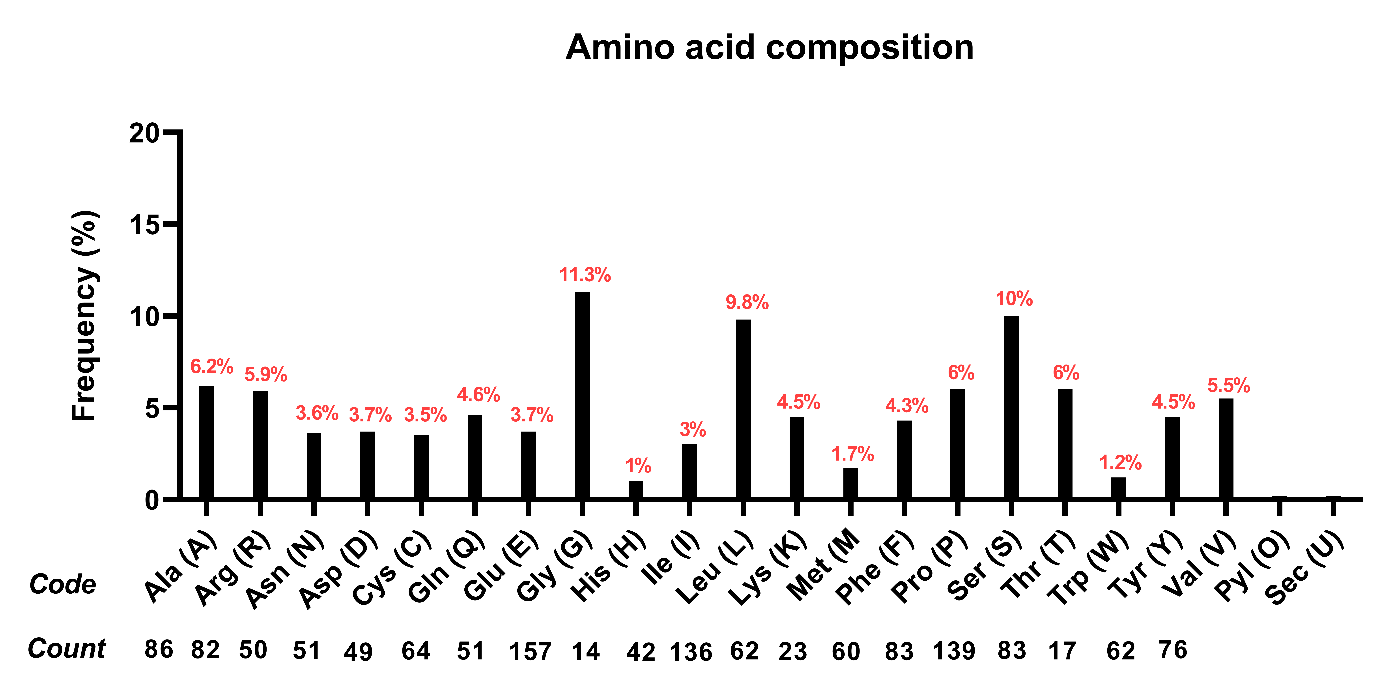


**Supplementary Fig S1:** Amino acid composition and frequency of the designed CAR


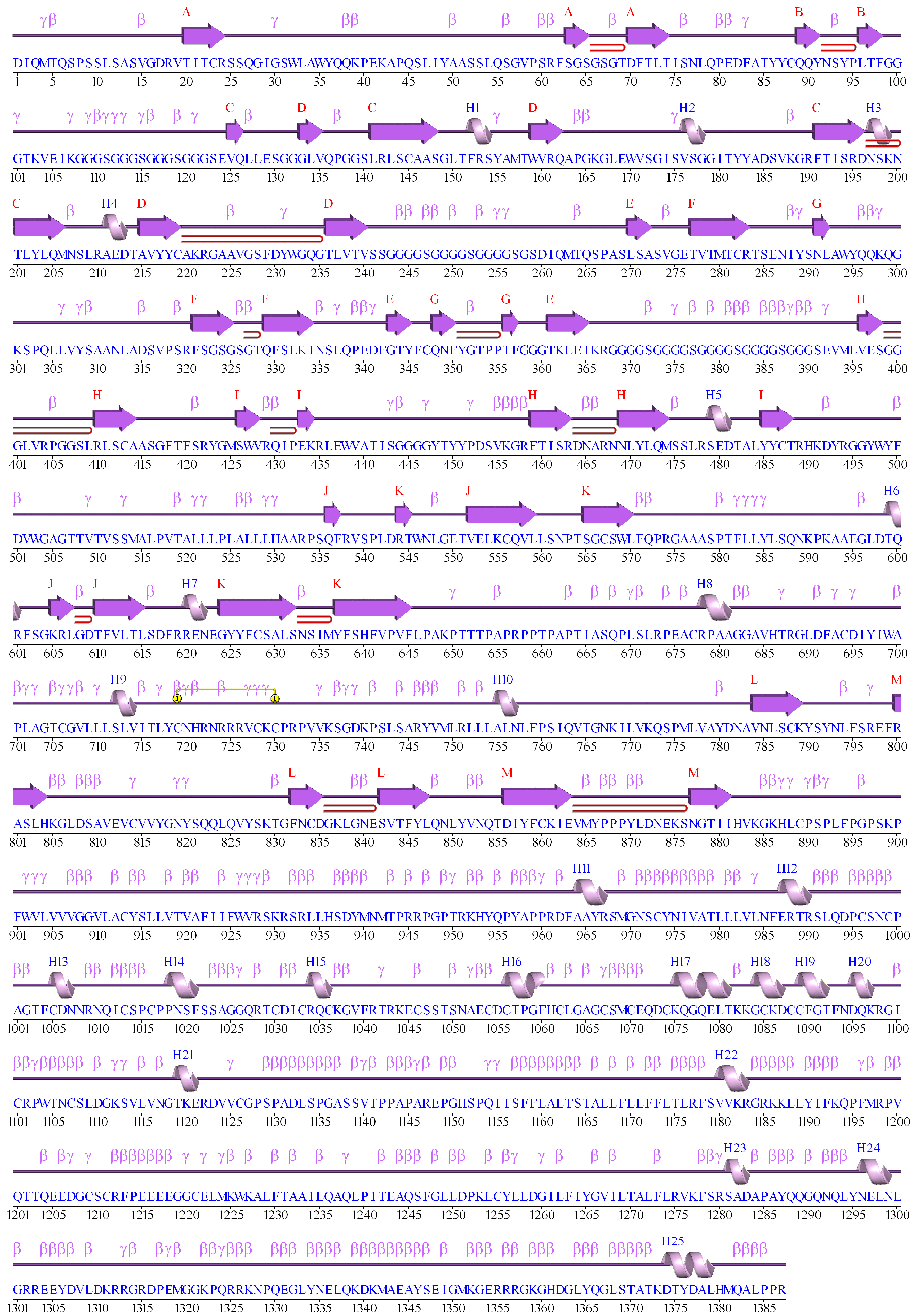


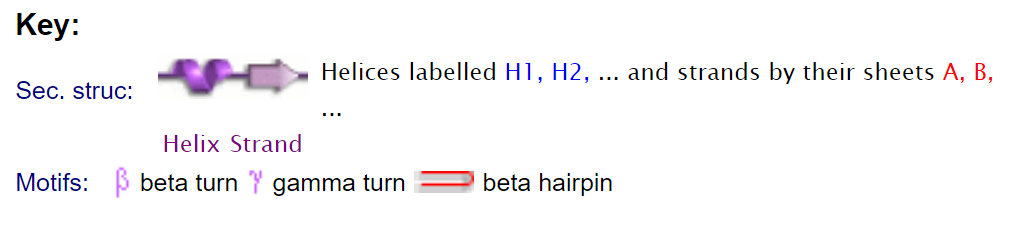


**Supplementary Fig S2:** 2D structure of the designed CAR

**
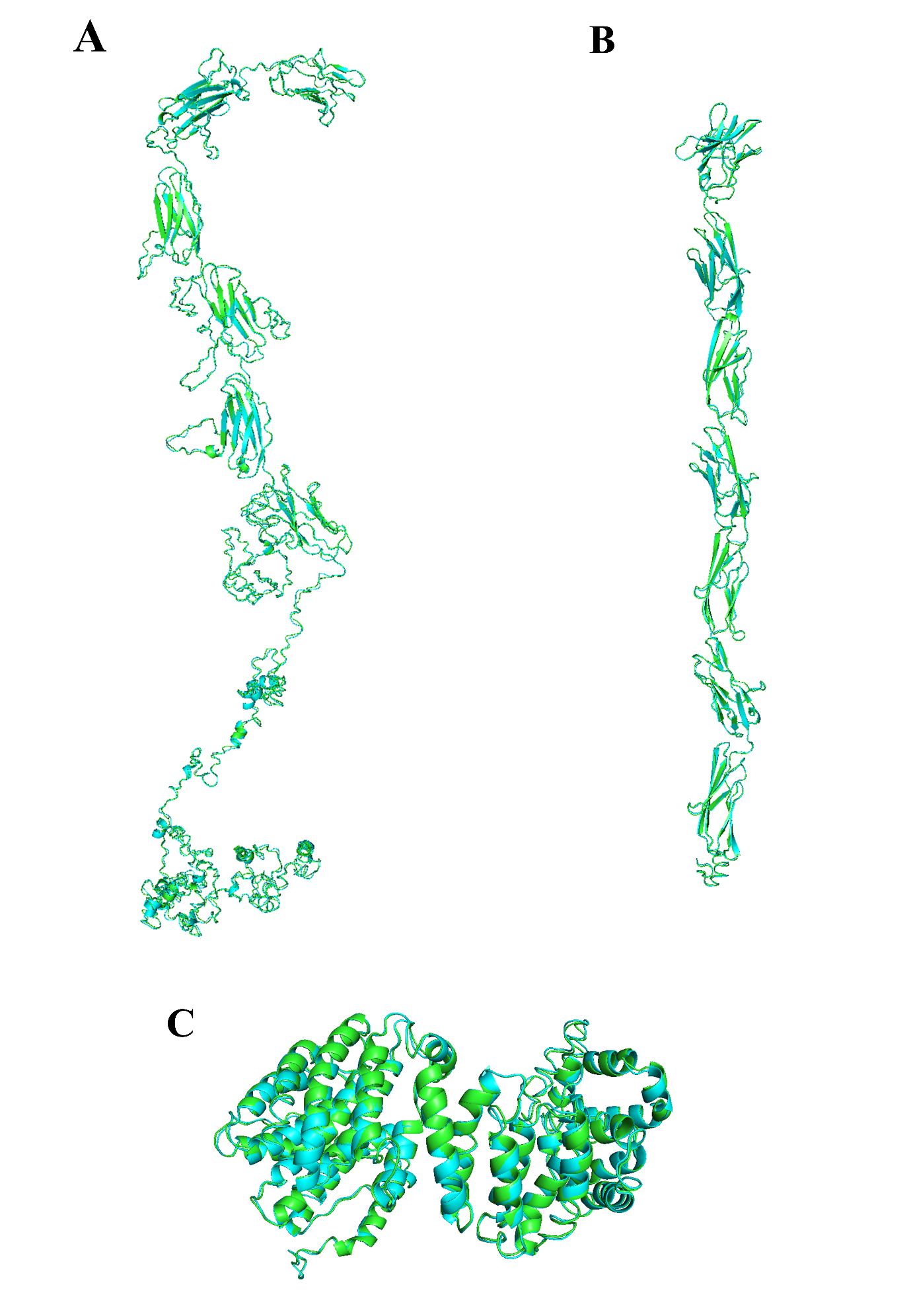
**

**Supplementary Fig S3:** 3D structure of the designed CAR **(A)**, CEA **(B)**, and MSLN **(C)**, where the unrefined model was shown in green color and the refined model was shown in cyan color respectively

**Supplementary Table S1: Clinical trials of CAR T cell therapies agaisnt Pancreactic ductal Adenocarcinoma (as of June 13, 2024)**

| **S.No** | **NCT Number** | **Study Title** | **Conditions** | **Interventions** | **Primary Outcome Measures** | **Phases** | **Enrollment** |
| --- | --- | --- | --- | --- | --- | --- | --- |
|  | NCT06072989 | Repeat Intravenous Infusions of B4T2-001 CAR-T Without Lymphodepleting Chemotherapy for Solid Tumors | Advanced Solid Tumor | BIOLOGICAL: B4T2-001 autologous CAR-T | Safety and tolerability of B4T2-001 CAR-T will be assessed by the incidence of serious adverse events (SAEs), incidence and severity of adverse events (AEs)., Multiple infusions of B4T2-001 CAR-T., 2 years after B4T2-001 CAR-T\|To determine the MTD and RP2D of B4T2-001 CAR-T, The MTD will be determined based on the occurrence of the DLTs according to dose escalation design. RP2D will be defined based on MTD, safety, PK, and preliminary efficacy data., 2 years after B4T2-001 CAR-T | PHASE1 | 24 |
|  | NCT02587689 | Phase I/II Study of Anti-Mucin1 (MUC1) CAR T Cells for Patients With MUC1+ Advanced Refractory Solid Tumor | Hepatocellular Carcinoma\|Non-small Cell Lung Cancer\|Pancreatic Carcinoma\|Triple-Negative Invasive Breast Carcinoma | BIOLOGICAL: anti-MUC1 CAR T Cells | Phase I: Adverse events attributed to the administration of the anti-MUC1 CAR T cells, 2 years | PHASE1\|PHASE2 | 20 |
|  | NCT04780529 | CART Therapy in Digestive System Tumors | Malignant Neoplasms of Digestive Organs | BIOLOGICAL: gucy2c cart cells | Number of participants with CAR-T treatment-related adverse events as assessed by CTCAE v4.03 [ Time Frame: 24 months ], The investigator is responsible for ensuring that all adverse events observed by the investigator or reported by the subject during the 3-month period from enrollment (i.e. initiation of leukocyte separation) to 3 months after targeted car-t infusion are monitored and reported. After three months, researchers will be required to monitor and report targeted adverse events, including neurological, blood, infection, autoimmune diseases, and secondary malignant tumors, for 24 months or until disease progression, whichever occurs first., 24 months | NA | 20 |
|  | NCT06010862 | Clinical Study of CEA-targeted CAR-T Therapy for CEA-positive Advanced/Metastatic Malignant Solid Tumors | Gastric Cancer\|Colon Cancer\|Pancreas Cancer\|Esophagus Cancer\|Cholangiocarcinoma\|Lung Cancer\|Breast Cancer | BIOLOGICAL: CEA CAR-T cells\|BIOLOGICAL: CEA CAR-T cells | Incidence of Adverse events after CEA CAR-T cells infusion [Safety and Tolerability], Therapy-related adverse events were recorded and assessed according to the National Cancer Institute's Common Terminology Criteria for Adverse Events (CTCAE, Version 5.0), 28 days\|Obtain the maximum tolerated dose of CEA CAR-T cells[Safety and Tolerability], Dose-limiting toxicity after cell infusion, 28 days | PHASE1 | 36 |
|  | NCT06134960 | NKG2D/CLDN18.21 CAR-T(KD-496) in the Treatment of Advanced NKG2DL+/CLDN18.2+ Solid Tumor | Gastric Cancer\|Pancreatic Cancer\|Solid Tumor | BIOLOGICAL: KD-496 | Incidence of Treatment Related adverse events (AEs), Incidence of Treatment Related AEs, AEs of special interest and serious adverse events (SAEs), 3 months after infusion\|Dose-limiting toxicity (DLT) rate, A drug-related toxicity during treatment with the drug, the severity of which is clinically unacceptable, limiting the further escalation of drug dose., 1 month after single infusion | PHASE1 | 12 |
|  | NCT05275062 | Clinical Trial to Evaluate the Safety and Efficacy of IM92 CAR-T Cells Therapy in Patients With Advanced Gastric or Pancreatic Adenocarcinoma | Advanced Solid Tumors\|Gastric Cancer\|Esophagogastric Junction Cancer\|Pancreatic Cancer | DRUG: IM92 CAR-T cells | Incidence of adverse events (AEs), Incidence of treatment related AEs, Up to 28 days after CAR-T cell infusion | EARLY_PHASE1 | 6 |
|  | NCT05472857 | Clinical Study of CLDN18.2-targeting CAR T Cells in Advanced Solid Tumors With Positive CLDN18.2 Expression | Gastric Cancer\|Pancreatic Cancer\|Advanced Ovarian Carcinoma\|Gastroesophageal Junction Adenocarcinoma | BIOLOGICAL: Claudin 18.2 CAR-T | Incidence of Treatment Related adverse events (AEs), Incidence of Treatment Related AEs, AEs of special interest and serious adverse events (SAEs), day1 - month12\|Identification of Maximum Tolerated Dose (MTD), Incidence of dose-limiting toxicities (DLTs), day1 - day28 | PHASE1 | 30 |
|  | NCT05736731 | A Study to Evaluate the Safety and Efficacy of A2B530, a Logic-gated CAR T, in Subjects With Solid Tumors That Express CEA and Have Lost HLA-A*02 Expression | Solid Tumor, Adult\|Solid Tumor\|Pancreatic Cancer\|Pancreatic Neoplasms\|Pancreas Cancer\|Non Small Cell Lung Cancer\|Non Small Cell Lung Cancer Recurrent\|Non-Small Cell Squamous Lung Cancer\|NSCLC\|NSCLC, Recurrent\|Colorectal Cancer\|Colorectal Neoplasms\|Colorectal Adenocarcinoma\|CRC\|Colorectal Cancer Metastatic\|Cancer | BIOLOGICAL: A2B530\|DIAGNOSTIC_TEST: xT-Onco with HLA-LOH Assay | Phase 1: Rate of adverse events and dose limiting toxicities (DLTs) by dose level, Adverse Events and toxicity will be evaluated according to the Cancer Therapy Evaluation Program Common Terminology Criteria for Adverse Events version 5.0 (or current version). Cytokine release syndrome (CRS) and immune effector cell-associated neurotoxicity syndrome (ICANS) events will be graded according to the criteria described in the current protocol., From the time of Informed consent until 24 months (2 years) post A2B530 infusion.\|Phase 1: Recommended Phase 2 Dose (RP2D), The RP2D will be identified utilizing a BOIN study design in addition to considering safety and biomarker analysis., 21 days post A2B530 infusion\|Phase 2: The Overall Response Rate (ORR) for patients, The ORR will be evaluated per RECIST v1.1 and assessed by independent central review., 24 months post A2B530 infusion | PHASE1\|PHASE2 | 160 |
|  | NCT03818165 | Phase 1b Study of CAR2Anti-CEA CAR-T Cell Hepatic Infusions for Pancreatic Carcinoma Patients With CEA+ Liver Metastases | Metastatic Pancreatic Carcinoma | BIOLOGICAL: CAR2 Anti-CEA CAR-T cells | Assess preliminary efficacy by overall survival, As a measure of activity, Overall Survival (OS) will be assessed. The events for the assessment of OS are death events. Time to event endpoints will be estimated using Kaplan-Meier methods. Point estimates and 95% confidence intervals will be provided where applicable., 6 months | PHASE1 | 2 |
|  | NCT05287165 | Clinical Trial to Evaluate the Safety and Efficacy of IM96 CAR-T Cells Therapy in Patients With Advanced Digestive System Neoplasms | Advanced Solid Tumors\|Digestive System Neoplasms\|Pancreatic Cancer Resectable\|Colorectal (Colon or Rectal) Cancer | DRUG: IM96 CAR-T cells | Incidence of Treatment Related adverse events (AEs), Incidence of treatment related AE., Up to 28 days after CAR-T cell infusion | EARLY_PHASE1 | 19 |
|  | NCT03932565 | Interventional Therapy Sequential With the Fourth-generation CAR-T Targeting Nectin4/FAP for Malignant Solid Tumors | Nectin4-positive Advanced Malignant Solid Tumor | BIOLOGICAL: CAR-T therapy for nectin4-positive malignant solid tumor | Adverse events that are related to treatment, Safety and tolerability measured by occurrence of study related adverse effects defined by NCI-CTCAE v4.03, 2 years | PHASE1 | 30 |
|  | NCT05946226 | A Phase I Trial to Evaluate the Safety of IMC002 in Advanced Digestive System Tumors | Advanced Digestive System Tumor | BIOLOGICAL: IMC002 injection | Incidence and severity of dose-limiting toxicity (DLTs) within 28 days after IMC002 infusion, safety profile, within 28 days | PHASE1 | 18 |
|  | NCT02416466 | CAR-T Hepatic Artery Infusions and Sir-Spheres for Liver Metastases | Liver Metastases | BIOLOGICAL: anti-CEA CAR-T cells\|DEVICE: Sir-Spheres | Safety of CAR-T cell hepatic artery infusions in combination with Sir-Spheres as Measured by Number of Participants with Adverse Events, To determine the safety and regimen limiting toxicity (RLT) of a standard of care treatment with Yttrium-90 Sir-Spheres Microspheres when following anti-CEA CAR-T hepatic artery infusions (HAI) for CEA-expressing liver metastases., 14 weeks | PHASE1 | 8 |
|  | NCT06196658 | Early Phase I Study of Autologous T Cells (EX02 CAR-T) for Unresectable Pancreatic/Bile Duct Cancer | Pancreatic Cancer\|Advanced Biliary Cancer | DRUG: anti-EX02 CAR T cells | Frequency and severity of treatment-related adverse events (TEAEs), Grade and type of toxicity per dose level; fraction of patients who experience toxicity (including allergic reactions to T cell infusions) of â‰¥ Grade 3 according to CTCAEv5.0, cytokine release syndrome (CRS) of â‰¥ Grade 3 according to ASTCT consensus and Immune Effector Cell-Associated Neurotoxicity Syndrome (ICANS)., 4 weeks after the first CAR-T cell infusion\|Objective Response Rate, Objective Response Rate (ORR) is the proportion of participants with an objective response (either a complete response \[CR\] or partial response \[PR\]) in participants who received at least 1 dose of EX02CART and at least the 6-week tumor evaluation as determined by the investigator according to RECIST v1.1., 24 weeks | EARLY_PHASE1 | 6 |
|  | NCT06084286 | Dual-targeting CLDN18.2 and PD-L1 CAR-T for Patients With CLDN18.2-positive Advanced Solid Tumors | Advanced Solid Tumor | BIOLOGICAL: Dual-targeting CLDN18.2 and PD-L1 CAR-T cells | Adverse Events (AEs), Safety evaluation. AEs will be recorded and evaluated by CTCAE 5.0., 28 days\|Dose-limiting toxicity (DLT), Tolerability evaluation. DLT will be assessed by CTCAE 5.0., 28 days\|Recommended phase II dose (RP2D), Efficacy dose., Approximately 18 months | PHASE1 | 29 |
|  | NCT01583686 | CAR T Cell Receptor Immunotherapy Targeting Mesothelin for Patients With Metastatic Cancer | Cervical Cancer\|Pancreatic Cancer\|Ovarian Cancer\|Mesothelioma\|Lung Cancer | DRUG: Fludarabine\|BIOLOGICAL: Anti-mesothelin chimeric T cell receptor (CAR) transduced peripheral blood lymphocytes (PBL)\|DRUG: Cyclophosphamide\|DRUG: Aldesleukin | Number of Patients With Objective Tumor Regression, Objective tumor regression response was assessed by the Response Evaluation Criteria in Solid Tumors (RECIST)v1.0. Complete Response (CR) is disappearance of all target lesions. Partial Response (PR) is at least a 30% decrease in the sum of the longest diameter (LD) of target lesions taking as reference the baseline sum LD. Stable Disease (SD) is neither sufficient shrinkage to qualify for PR nor sufficient increase to qualify for progressive disease taking as reference the smallest sum LD. Progressive Disease (PD) is at least a 20% increase in the sum of the longest diameter (LD) of target lesions taking as reference the smallest sum LD recorded since the treatment started or the appearance of one or more new lesions., 3.5 mos.\|Number of Participants With Serious and Non-serious Adverse Events Assessed by the Common Terminology Criteria in Adverse Events (CTCAE v4.0), Here is the count of participants with serious and non-serious adverse events assessed by the Common Terminology Criteria in Adverse Events (CTCAE v4.0). A non-serious adverse event is any untoward medical occurrence. A serious adverse event is an adverse event or suspected adverse reaction that results in death, a life-threatening adverse drug experience, hospitalization, disruption of the ability to conduct normal life functions, congenital anomaly/birth defect or important medical events that jeopardize the patient or subject and may require medical or surgical intervention to prevent one of the previous outcomes mentioned., Date treatment consent signed to date off study, approximately 6 months and 17 days for Group A01, 16 months and 13 days for Group A02, 13 months and 3 days for Group A03, 10 months and 16 days for Group A04, and 11 months and 26 days for Group A05. | PHASE1\|PHASE2 | 15 |
|  | NCT03013712 | A Clinical Research of CAR T Cells Targeting EpCAM Positive Cancer | Colon Cancer\|Esophageal Carcinoma\|Pancreatic Cancer\|Prostate Cancer\|Gastric Cancer\|Hepatic Carcinoma | BIOLOGICAL: CAR-T cell immunotherapy | Toxicity profile of the EpCAM targeted CAR T cells with Common Toxicity Criteria for Adverse Effects (CTCAE) version 4.0, Observe and handle the toxicity profile of the EpCAM targeted CAR T cells with Common Toxicity Criteria for Adverse Effects (CTCAE) version 4.0, up to 24 months | PHASE1\|PHASE2 | 60 |
|  | NCT05284968 | RD07 Cell Injection in the Treatment of Patients With Advanced Claudin18.2 Positive Solid Tumors | Solid Tumor | DRUG: Cell injection | Dose-limiting toxicity (DLT) and maximum tolerated dose (MTD), 3 years\|Adverse events (TEAEs) and incidence after initial infusion, treatment-related adverse events and incidence, AESI and incidence of special concern, 3 years | EARLY_PHASE1 | 36 |
|  | NCT05583201 | NKG2D/CLDN18.2 CAR-T(KD-496) in the Treatment of Advanced NKG2DL+/CLDN18.2+ Solid Tumors | Gastric Cancer\|Pancreatic Cancer\|Solid Tumor | BIOLOGICAL: KD-496 | treatment-emergent adverse events(TEAEs), An adverse event is any undesirable experience associated with the use of a medical product in a patient, 3 months after single infusion\|Dose-limiting toxicity (DLT) rate, A drug-related toxicity during treatment with the drug, the severity of which is clinically unacceptable, limiting the further escalation of drug dose., 3 months after single infusion\|CAR positive T cells in patients, The time of CAR-T cell reach the peak and turn back to baseline, 6 months after single infusion | EARLY_PHASE1 | 18 |
|  | NCT03323944 | CAR T Cell Immunotherapy for Pancreatic Cancer | Pancreatic Cancer\|Cancer of the Pancreas | BIOLOGICAL: huCART-meso cells | Number of study subjects with treatment-related adverse events using NCI Common Terminology Criteria for Adverse Events (CTCAE) v4.03, 2 years | PHASE1 | 18 |
|  | NCT02706782 | A Study of Mesothelin Redirected Autologous T Cells for Advanced Pancreatic Carcinoma | Pancreatic Cancer | DRUG: TAI-meso-CART | Number of patients with adverse event, asverse event is evaluated with CTCAE, version 4.0, 6 weeks | PHASE1 | 30 |
|  | NCT03960060 | A Study of CCT301-59 CAR T Therapy in Adult Subjects With Recurrent or Refractory Solid Tumors | Solid Tumor\|Soft Tissue Sarcoma\|Gastric Cancer\|Pancreatic Cancer\|Bladder Cancer | BIOLOGICAL: CCT301-59 | Safety of CCT301-59 CAR T cell therapy, To observe the safety of CCT301-59 CAR positive T cells in subjects using Common Toxicity Criteria for Adverse effects (CTCAE) version 5.0., Up to 52 weeks\|Efficacy of CCT301-59 CAR T cell therapy, Objective response, such as complete response (CR), partial response (PR), stable disease (SD), or progressive disease (PD) will be assessed by the Response Evaluation Criteria in Solid Tumors (RECIST) v1.1 criteria., Up to 52 weeks\|Kinetics of CAR T cells, The level of CAR T cells in the peripheral blood and persistence of CAR T cells in patients will be tested., Up to 52 weeks | PHASE1 | 18 |
|  | NCT03682744 | CAR-T Intraperitoneal Infusions for CEA-Expressing Adenocarcinoma Peritoneal Metastases or Malignant Ascites (IPC) | Peritoneal Carcinomatosis\|Peritoneal Metastases\|Colorectal Cancer\|Gastric Cancer\|Breast Cancer\|Pancreas Cancer\|Carcinoembryonic Antigen | BIOLOGICAL: anti-CEA CAR-T cells | Safety of Intraperitoneal CAR-T Cell Infusions as Measured by Number of Participants with Adverse Events, To determine the safety and maximum tolerated dose (MTD) following intraperitoneal infusion(s) of anti-CEA CAR-T cells for inoperable CEA+ peritoneal metastases or malignant ascites., 16 weeks | PHASE1 | 0 |
|  | NCT06043466 | A Clinical Trial Targeting CEA Chimeric Antigen Receptor T (CAR-T) for CEA Positive Advanced Malignant Solid Tumors | Colorectal Cancer\|Esophagus Cancer\|Gastric Cancer\|Pancreas Cancer\|Non-small Cell Lung Cancer\|Breast Cancer\|Bile Duct Cancer | BIOLOGICAL: CEA-targeted CAR-T cells | To determine the dose range and DLT of C-13-60 cells for CEA positive advanced malignant solid tumors [Safety and Tolerability], The incidence of adverse events (TEAE) after treatment; Incidence of treatment-related adverse events; Adverse evens of Special Interest (AESI);, 1 month\|To obtain the maximum tolerable dose of C-13-60 cells [Safety and Tolerability], Incidence and number of dose-limiting toxicity (DLT) casesï¼ŒDose-limiting toxicity after CAR-T cell infusion, 1month | PHASE1 | 30 |
|  | NCT03267173 | Evaluate the Safety and Efficacy of CAR-T in the Treatment of Pancreatic Cancer. | Pancreatic Cancer\|CAR | DRUG: Chimeric antigen receptor T cell | Number of patients with tumor response, Tumor response is assessmented with Response Evaluation Criteria in Solid Tumors (RECIST) Version 1.1, 8 weeks | EARLY_PHASE1 | 10 |
|  | NCT04581473 | Study to Evaluate the Efficacy, Safety and Pharmacokinetics of CT041 Autologous CAR T-cell Injection | Gastric Adenocarcinoma\|Pancreatic Cancer\|Gastroesophageal Junction Adenocarcinoma | DRUG: CT041 autologous CAR T-cell injection\|DRUG: Physician's Choiceï¼ˆPaclitaxel or Irinotecan or Apatinib or Anti-PD-1 antibodyï¼‰ | Phase Ib: Incidence of Treatment Related adverse events (AEs), Incidence of treatment related AEs, AEs of special interest and serious adverse events(SAEs)., Up to 18 months\|Phase Ib: Identification of Maximum Tolerated Dose (MTD), Incidence of dose-limiting toxicities (DLTs), day1-day28\|Phase II: Progression-free survival (PFS), as assessed by IRC, of CT041 autologous CAR T-cell injection versus Physician's Choice, Progression-free survival (PFS) was defined as the time from the date of randomization to the earliest date of the first objective documentation of progressive disease (PD) or death due to any cause., Up to 24 months | PHASE1\|PHASE2 | 192 |
|  | NCT05673434 | A Clinical Study on the Safety and Efficacy of CAR-T Therapy for the TM4SF1-positive Tumors of Digestive System | Digestive Tumor | BIOLOGICAL: TM4SF1-positive chimeric antigen receptor T-cell therapy | Safety assessed by Incidence of Treatment-Emergent Adverse Events (AEs) and Serious Adverse Events (SAEs), After CAR-T cell infusion,the investigators will observe the potential adverse events related to the CAR-T cells infusion such as high fever, kidney failure and so on.  Adverse events are coded according to MedDRA 22.0. List total number of AEs and SAEs; Number of subjects with different types of AEs and SAEs, case-times and incidence.AEs and SAEs are graded by National Cancer Institute Common Terminology Criteria for Adverse Events (NCI CTC AE version 5.0)., 2 years | NA | 24 |
|  | NCT05779917 | Mesothelin/GPC3/GUCY2C-CAR-T Cells Against Cancers | Pancreas Cancer\|CAR-T Cell Therapy\|Mesothelin\|Solid Tumor, Adult | BIOLOGICAL: CAR-T cells | Number of Patients with Dose Limiting Toxicity, A dose limiting toxicity is defined as any toxicity that is considered to be primarily related to the Mesothelin-CAR T cells, which is irreversible, or life threatening or hematologic or non-hematologic Grade 3-5., six months | PHASE1 | 30 |
|  | NCT02349724 | A Clinical Research of CAR T Cells Targeting CEA Positive Cancer | Lung Cancer\|Colorectal Cancer\|Gastric Cancer\|Breast Cancer\|Pancreatic Cancer | BIOLOGICAL: Anti-CEA-CAR T | Adverse events of each patient., Determine the toxicity profile of the CEA targeted CAR T cells with Common Toxicity Criteria for Adverse Effects (CTCAE) version 4.0., 3 years | PHASE1 | 75 |
|  | NCT05911217 | A Study to Evaluate the Efficacy and Safety of CT041 After Adjuvant Chemotherapy for Pancreatic Cancer | Pancreatic Cancer | DRUG: CT041 autologous CAR T-cell injection | Disease free survival (DFS), The time from the first infusion to the occurrence of local recurrence/distant metastasis or death from any cause, whichever occurred first., Up to 18 months | PHASE1 | 20 |
|  | NCT04981119 | Solid Tumor Analysis for HLA Loss of Heterozygosity (LOH) and Apheresis for CAR T- Cell Manufacturing | Solid Tumor, Adult\|Colorectal Cancer\|Non Small Cell Lung Cancer\|Pancreatic Cancer\|CRC\|NSCLC\|Pancreas Cancer\|Mesothelioma\|Ovarian Cancer\|Ovarian Neoplasms\|Ovarian Carcinoma\|Mesothelioma, Malignant\|Mesothelioma; Lung\|Cancer | OTHER: Apheresis\|DIAGNOSTIC_TEST: Next Generation Sequencing (NGS)\|DIAGNOSTIC_TEST: Long Range NGS HLA typing | Percentage of participants who can enroll in an A2 Biotherapeutics, Inc. CAR T-cell therapy study after undergoing apheresis, Participants will be followed for their status of enrollment on an A2 Biotherapeutics, Inc. interventional study, up to 2 years\|Percentage of screened participants experiencing loss of heterozygosity (LOH) of HLA-A*02 identified by next generation sequencing, Percentage of participants experiencing LOH will be calculated based on NGS results, Screening |  | 200 |
|  | NCT05393986 | Claudin18.2-redirected Chimeric Antigen Receptor T Cells With Co-expression of Cytokines in Solid Tumors | Gastric Adenocarcinoma\|Pancreatic Cancer\|Gastroesophageal Junction Adenocarcinoma | DRUG: CT048 Autologous Injection (CT048) | Dose-limiting toxicity (DLT), Safety, 28 days of single infusion\|Maximum tolerated dose, Tolerability, 28 days of single infusion | PHASE1 | 63 |
|  | NCT04151186 | A Clinical Study on the Safety and Efficacy of CAR-T Therapy for the TM4SF1- and EpCAM-positive Solid Tumors | Advanced Solid Tumor\|Neoplasms | BIOLOGICAL: TM4SF1- and EpCAM-positive chimeric antigen receptor T-cell therapy | Safety assessed by Incidence of Treatment-Emergent Adverse Events (AEs) and Serious Adverse Events (SAEs), 1. After CAR-T cell infusion,the investigators will observe the potential adverse events related to the CAR-T cells infusion such as high fever, kidney failure and so on. 2. Adverse events are coded according to MedDRA 22.0. List total number of AEs and SAEs; Number of subjects with different types of AEs and SAEs, case-times and incidence.AEs and SAEs are graded by National Cancer Institute Common Terminology Criteria for Adverse Events (NCI CTC AE version 5.0)., 2 years | NA | 72 |
|  | NCT05621486 | A Clinical Study to Evaluate B4T2-001 CAR T Cells in the Treatment of Advanced Solid Tumors | Advanced Solid Tumor | BIOLOGICAL: B4T2-001 Autologous CAR T cells | Incidence of serious adverse events (SAEs), incidence and severity of adverse events (AEs), Safety and tolerability of B4T2-001 CAR T cells, Minimum 2 years after B4T2-001 CAR T infusion\|To determine the maximum tolerated dose (MTD) and recommended Phase 2 dose (RP2D) of B4T2-001 CAR T cells, The MTD will be determined based on the occurrence of the Dose-Limiting Toxicities (DLTs) according to the accelerated titration design and 3+3 dose escalation design. RP2D will be defined based on MTD, safety, PK, and preliminary efficacy data., 2 years after B4T2-001 CAR T infusion | PHASE1 | 36 |
|  | NCT04037241 | Study of Anti-CEA CAR-T + Chemotherapy VS Chemotherapy Alone in Patients With CEA+Pancreatic Cancer & Liver Metastases | Malignant Tumor of Pancreas Metastatic to Liver | BIOLOGICAL: Anti-CEA CAR-T cells\|DRUG: gemcitabine/nab paclitaxel\|DRUG: NLIR+FU/FA\|DRUG: Capecitabine | Assess efficacy by overall survival, As a measure of activity, Overall Survival (OS) will be assessed. The events for the assessment of OS are death events. Time to event endpoints will be estimated using Kaplan-Meier methods. Point estimates and 95% confidence intervals will be provided where applicable., 6 - 12 months | PHASE2\|PHASE3 | 0 |
|  | NCT03159819 | Clinical Study of CAR-CLD18 T Cells in Patients With Advanced Gastric Adenocarcinoma and Pancreatic Adenocarcinoma | Advanced Gastric Adenocarcinoma\|Pancreatic Adenocarcinoma | GENETIC: CAR-CLD18 T Cells | Safety and tolerance, During the trial conduction, especially within the 24 weeks of treatment phase when CAR-CLD18 T cell administered, all adverse events (including laboratory abnormality and clinical events) will be closely monitored, and all â‰¥ grade 3 adverse events per CTCAE (v 3.0) will be recorded, including but not limited to the toxicities potentially suspected to relate to infusion procedures and/or CAR-CLD18 T cell therapy as listed below:  * Fever * Chills * Nausea, vomiting and other gastrointestinal symptoms * Fatigue * Hypotension * Respiratory distress * Tumor lysis syndrome * Cytokine release syndrome * Neutropenia, thrombocytopenia * Liver and kidney dysfunction, 24 weeks | NA | 24 |
|  | NCT06158139 | Autologous CAR-T Cells Targeting B7-H3 in PDAC | Pancreas Cancer\|Relapse\|Resistant Cancer | BIOLOGICAL: iC9-CAR.B7-H3 T cell infusion | Number of participants with adverse event, Number of participants with adverse event (AE)s as a measure of safety and tolerability of intraventricular administration iC9-CAR.B7-H3 T cells in subjects with progressive recurrent or refractory pancreatic cancer.  AEs will be classified and graded according to the National Cancer Institute's Common Terminology Criteria for Adverse Events (NCI-CTCAE) version 5.0. Dose Limiting Toxicities (DLTs) are defined as at least possibly related to iC9-CAR.B7-H3 T cell product administration., Up to 4 weeks\|Cytokine Release Syndrome, Cytokine Release Syndrome (CRS) will be graded according to the American Society for Transplantation and Cellular Therapy (ASTCT) CRS Consensus Grading.  Grade 1 - Mild (Symptomatic Management): Fever â‰¥38\^ o C, No hypotension, No hypoxia, Grade 2 - Moderate (Moderate Intervention): Fever â‰¥38\^ o C, Hypotension not requiring vasopressors, Hypoxia requiring low-flow nasal cannula (â‰¤6 L/minute) or blow-by, Grade 3 - Severe (Aggressive Intervention): Fever â‰¥ 38\^ o C, Hypotension requiring a vasopressor with or without vasopressin, Hypoxia requiring high-flow nasal cannula (\>6 L/minute), facemask, nonrebreather mask, or Venturi mask, Grade 4 - Life-threatening (Life-sustaining intervention): Fever â‰¥38\^oC, Hypotension requiring multiple vasopressors (excluding vasopressin), Hypoxia requiring positive pressure (e.g. Continuous positive airway pressure, BiPAP, intubation, mechanical ventilation), Grade 5 - Death: Death., Up to 4 weeks\|Neurotoxicity, Neurotoxicity will be graded according to the Central Nervous System (CNS) Toxicity criteria.  Grade 0: Normal or no change from baseline exam at start of therapy, Grade 1: Mild lethargy and/or irritability or visual, motor, or sensory symptoms without change in neurological exam, Grade 2: Moderate lethargy, disorientation, or psychosis lasting \< 48 hours or mild increase in pre-existing neurological deficit, Grade 3: \>48hours of severe lethargy, but responsive to verbal stimuli or disorientation or psychosis lasting \>48 hours, Grade 4: Coma, unresponsive to verbal stimuli, increasing neurological deficit above grade 3, evidence of herniation, development of uncontrolled seizures, intracerebral hemorrhage., Up to 4 weeks | PHASE1 | 27 |
|  | NCT04025216 | A Study of CART-TnMUC1 in Patients With TnMUC1-Positive Advanced Cancers | Non-Small Cell Lung Cancer\|Ovarian Cancer\|Fallopian Tube Cancer\|Triple Negative Breast Cancer\|Multiple Myeloma\|Pancreatic Ductal Adenocarcinoma | BIOLOGICAL: CART-TnMUC1\|DRUG: Cyclophosphamide\|DRUG: Fludarabine | Dose Escalation: Dose Identification of CART-TnMUC1, Incidence of Dose Limiting Toxicity (DLT) in solid tumors and multiple myeloma, Up to 2 years\|Cohort Expansion: Objective Response in solid tumors, Proportion of patients having a confirmed Complete Response (CR) or Partial Response (PR) per Response Evaluation Criteria in Solid Tumors (RECIST) v1.1, Up to 2 years | PHASE1 | 16 |
|  | NCT02159716 | CART-meso in Mesothelin Expressing Cancers | Metastatic Pancreatic (Ductal) Adenocarcinoma\|Epithelial Ovarian Cancer\|Malignant Epithelial Pleural Mesothelioma | BIOLOGICAL: CART-meso | Number of Adverse Events, 2 years | PHASE1 | 19 |
|  | NCT02830724 | Administering Peripheral Blood Lymphocytes Transduced With a CD70-Binding Chimeric Antigen Receptor to People With CD70 Expressing Cancers | Pancreatic Cancer\|Renal Cell Cancer\|Breast Cancer\|Melanoma\|Ovarian Cancer | DRUG: Cyclophosphamide\|DRUG: Fludarabine\|DRUG: Aldesleukin\|BIOLOGICAL: Anti-hCD70 CAR transduced PBL | Frequency and severity of treatment-related adverse events, Grade and type of toxicity per dose level; fraction of patients who experience a DLT at a given dose level, and number and grade of each type of DLT, From time of cell infusion to two weeks after cell infusion\|Response rate, Percentage of patients who have a clinical response (PR+CR) to treatment (objective tumor regression), 6 weeks and 12 weeks following administration of the cell product, then every 3 months x3, then every 6 months x 2 years, then per PI discretion | PHASE1\|PHASE2 | 124 |
|  | NCT04119024 | Gene Modified Immune Cells (IL13Ralpha2 CAR T Cells) After Conditioning Regimen for the Treatment of Stage IIIC or IV Melanoma or Metastatic Solid Tumors | Metastatic Malignant Solid Neoplasm\|Metastatic Melanoma\|Pathologic Stage IIIC Cutaneous Melanoma AJCC v8\|Pathologic Stage IV Cutaneous Melanoma AJCC v8\|Recurrent Malignant Solid Neoplasm\|Refractory Malignant Solid Neoplasm\|Uveal Melanoma\|Acral Melanoma\|Neuroendocrine Tumors\|Paraganglioma\|Pheochromocytoma\|Adrenocortical Carcinoma\|Pancreatic Neuroendocrine Tumor\|Thyroid Cancer\|Breast Cancer\|Lung Adenocarcinoma\|Head and Neck Squamous Cell Carcinoma | PROCEDURE: Biopsy\|PROCEDURE: Biospecimen Collection\|PROCEDURE: Computed Tomography\|DRUG: Cyclophosphamide\|DRUG: Fludarabine Phosphate\|OTHER: Fludeoxyglucose F-18\|BIOLOGICAL: IL13Ralpha2-specific Hinge-optimized 4-1BB-co-stimulatory CAR/Truncated CD19-expressing Autologous TN/MEM Cells\|PROCEDURE: Magnetic Resonance Imaging\|PROCEDURE: Positron Emission Tomography | Incidence of adverse events, Safety will be reported as incidence rates for adverse events, serious adverse events, and fatal adverse events for Common Terminology Criteria for Adverse Events (CTCAE) version 5.0 grade 3 or higher. Adverse events will be tabulated by treatment group and will include the number of patients for whom the event occurred, the rate of occurrence, and the severity and relationship to study drug., Up to 90 days from the day of chimeric antigen receptor (CAR)-transgenic cell infusion\|Dose-limiting toxicity, Up to 28 days from the day of CAR-transgenic cell infusion | PHASE1 | 18 |
|  | NCT04203459 | The Mechanism of Enhancing the Anti-tumor Effects of CAR-T on PC by Gut Microbiota Regulation | Pancreatic Cancer\|Gut Microbiota\|CAR-T |  | In vivo experiment: comparison of intestinal microflora and T cell cholesterol metabolism and subtypes between pancreatic cancer patients and healthy people The difference of proportion and the correlation., Fecal and peripheral blood samples were collected from patients with pancreatic cancer and healthy people respectively: (1) through Illumina miseq  High throughput sequencing technology was used to detect 16S rRNA to analyze the diversity of intestinal flora (Î± and Î² diversity), and bioinformatics methods such as random forest and lefse were used to analyze the diversity of flora composition; (2) the difference of cholesterol content in T cells was detected by ELISA; (3) the difference of ACAT-1 expression in T cells was detected by PCR and WB; (4) the difference of ACAT-1 expression in T cells was detected by flow cytometry. The proportion of CD8 +, TNF Î± +, Th1, Th2, Th17, Treg and other subtypes in T cells was detected by cell analyzer. Furthermore, the correlation between intestinal microflora composition and peripheral blood T cell cholesterol metabolism and subtype difference was determined by Spearman correlation analysis (statistical method)., from January 2020 to December 2020.\|In vitro experiment: to verify the regulation of intestinal flora on the biological behavior of anti msln car-t in vitro and the role of ACAT-1 in it., In vitro, intestinal flora supernatant of pancreatic cancer and healthy human were co-cultured with anti-msln car-t, respectively, to verify the effect of intestinal flora on biological behavior of anti-msln car-t.To analyze how the metabolism of cholesterol in anti-msln car-t cells, with acat-1 as the key enzyme, participates in the process of regulating the biological behavior of anti-msln car-t by intestinal flora, and preliminarily uncover the relevant mechanism., from January 2021 to December 2021.\|Animal model experiment: to verify the ability of intestinal bacteria to control anti msln car-t targeted killing pancreatic cancer cells., Furthermore, the influence of differences in intestinal flora composition between pancreatic cancer and healthy human on anti-msln car-t function was verified in animal models of pancreatic cancer., from January 2022 to December 2022. |  | 80 |
|  | NCT05415475 | Clinical Study of CEA-targeted CAR-T Therapy for CEA-positive Advanced Malignant Solid Tumors | Colorectal Cancer\|Esophageal Cancer\|Stomach Cancer\|Pancreatic Cancer\|Metastatic Tumor\|Recurrent Cancer | BIOLOGICAL: CEA CAR-T cells | Incidence of Adverse events after CEA-CAR-T cells infusion [Safety and Tolerability], Therapy-related adverse events were recorded and assessed according to the National Cancer Institute's Common Terminology Criteria for Adverse Events (CTCAE, Version 5.0), 28 days\|Obtain the maximum tolerated dose of CEA-CAR-T cells[Safety and Tolerability], Dose-limiting toxicity after cell infusion, 28 days | PHASE1 | 36 |
|  | NCT03638206 | Autologous CAR-T/TCR-T Cell Immunotherapy for Malignancies | B-cell Acute Lymphoblastic Leukemia\|Lymphoma\|Myeloid Leukemia\|Multiple Myeloma\|Hepatoma\|Gastric Cancer\|Pancreatic Cancer\|Mesothelioma\|Colorectal Cancer\|Esophagus Cancer\|Lung Cancer\|Glioma\|Melanoma\|Synovial Sarcoma\|Ovarian Cancer\|Renal Carcinoma | BIOLOGICAL: CAR-T cell immunotherapy | Number of Participants With Adverse Events evaluated with NCI CTC AE, version 4.0, Safety evaluation, 60 months | PHASE1\|PHASE2 | 73 |
|  | NCT03497819 | Autologous CARTmeso/19 Against Pancreatic Cancer | Pancreatic Cancer | BIOLOGICAL: CARTmeso CART19 | The Percentage of Adverse Events (AEs) â‰¥ grade 3 assessed through MedDra and CTCAE v4.03 [Time frame: from infusion to 3 month afterward], Primary outcome is the percentage of adverse events (AEs) â‰¥ grade 3. AEs are assessed through MedDra and CTCAE v4.03. Any patients who receive any dose of CART cells will be evaluated, From first infusion to 3 months afterward | EARLY_PHASE1 | 10 |
|  | NCT02541370 | Treatment of Relapsed and/or Chemotherapy Refractory Advanced Malignancies by CART133 | Liver Cancer\|Pancreatic Cancer\|Brain Tumor\|Breast Cancer\|Ovarian Tumor\|Colorectal Cancer\|Acute Myeloid and Lymphoid Leukemias | BIOLOGICAL: anti-CD133-CAR vector-transduced T cells | Occurrence of study related adverse events, defined as \>= Grade 3 signs/symptoms, laboratory toxicities, and clinical, Until week 24 | PHASE1\|PHASE2 | 20 |
|  | NCT05539430 | Claudin 18.2-Targeted Chimeric Antigen Receptor T-cells in Subjects With Unresectable, Locally Advanced, or Metastatic Gastric, Gastroesophageal Junction (GEJ), Esophageal, or Pancreatic Adenocarcinoma | Gastric Cancer\|Gastroesophageal-junction Cancer\|Esophageal Cancer\|Pancreatic Cancer | BIOLOGICAL: LB1908 | To characterize the safety and tolerability of LB1908 and determine the optimal dose or recommended dose for expansion (RDE), Multiple doses will be tested to establish a recommended dose., 28 days\|To further characterize the safety and tolerability of LB1908 with the RDE identified in the dose-escalation and determine the recommended Phase 2 dose (RP2D), Treatment of additional patients at the recommended dose as identified in the initial dose escalation part of the study., 90 days | PHASE1 | 56 |
|  | NCT02850536 | CAR-T Hepatic Artery Infusions or Pancreatic Venous Infusions for CEA-Expressing Liver Metastases or Pancreas Cancer | Liver Metastases | BIOLOGICAL: anti-CEA CAR-T cells | Safety of CAR-T cell hepatic artery infusions delivered using the Surefire Infusion System (SIS) as Measured by Number of Participants with Adverse Events, To determine the safety and regimen limiting toxicity (RLT) of anti-CEA CAR-T hepatic artery infusions (HAI) via the Surefire Infusion System (SIS) for CEA-expressing liver metastases, 10 weeks | PHASE1 | 5 |
|  | NCT06126406 | Clinical Study of CEA Targeting Chimeric Antigen Receptor T Lymphocytesï¼ˆCAR-Tï¼‰ for CEA Positive Advanced Malignant Solid Tumors | Gastric Cancer\|Colon Cancer\|Rectal Cancer\|Breast Cancer\|Lung Cancer\|Esophagus Cancer\|Cholangiocarcinoma\|Pancreas Cancer | BIOLOGICAL: CEA-targeted CAR-T cells\|BIOLOGICAL: CEA-targeted CAR-T cells | Incidence of Treatment-associated Adverse Events [Safety and Tolerability], The incidence of adverse events after CEA CAR-T cell infusion was assessed by the National Cancer Institute's Common Terminology Criteria for Adverse Events (CTCAE, version 5.0), 1 month\|Obtained the recommended dose and infusion regimen of CAR-T cells for the treatment of patients with CEA-positive advanced malignancies[Safety and Tolerability], Dose-limiting toxicity after CEA CAR-T cell infusion, 28 days | PHASE1 | 60 |
|  | NCT06256055 | Phase 1 Study of UCMYM802 Injection in Mesothelin-positive Advanced Malignant Solid Tumors | Malignant Mesothelioma\|Colorectal Cancer\|Bile Duct Cancer\|Rectal Cancer\|Ovary Cancer\|Pancreatic Cancer\|Breast Cancer Female | BIOLOGICAL: UCMYM802 Injection | Treatment Emergent Adverse Event (TEAE), Incidence and severity of treatment emergent adverse events., 2 years\|Treatment Related Adverse Event (TRAE), Incidence and severity of treatment related adverse events, 2 years\|Adverse Events of Special Interest (AESI), Incidence and severity of adverse event of special interest, 2 years\|Incidence of Dose-limiting Toxicities (DLTs), Incidence and severity of dose-limiting toxicities (DLTs) following infusion of UCMYM802 injection at each dose level., 4 weeks | PHASE1 | 24 |
|  | NCT06054308 | Mesothelin-targeted CAR-T Cells as a Neo-adjuvant Treatment in Patients With Resectable Pancreatic Cancers: a Feasibility Study | Pancreatic Cancer | OTHER: MSLN CART | pathologic response, Proportion of major pathologic response on resected specimen of pancreatic tumour, 3 months | NA | 10 |
|  | NCT06051695 | A Study to Evaluate the Safety and Efficacy of A2B694, a Logic-gated CAR T, in Subjects With Solid Tumors That Express MSLN and Have Lost HLA-A*02 Expression | Solid Tumor, Adult\|Colorectal Cancer\|NSCLC\|Non Small Cell Lung Cancer\|NSCLC, Recurrent\|Non-Small Cell Squamous Lung Cancer\|Pancreas Cancer\|Pancreatic Neoplasm\|Colorectal Adenocarcinoma\|CRC\|Colon Cancer\|Rectal Cancer\|Cancer\|Ovarian Cancer\|Ovarian Neoplasms\|Mesothelioma\|Mesothelioma, Malignant\|Ovary Cancer\|Lung Cancer\|MESOM | BIOLOGICAL: A2B694\|DIAGNOSTIC_TEST: xT CDx with HLA-LOH Assay | Phase 1: Rate of adverse events and dose limiting toxicities (DLTs) by dose level, Adverse Events and toxicity will be evaluated according to the Cancer Therapy Evaluation Program Common Terminology Criteria for Adverse Events version (CTCAE) 5.0 (or current version). Cytokine release syndrome (CRS) and immune effector cell-associated neurotoxicity syndrome (ICANS) events will be graded according to the criteria described in the current protocol., From the time of Informed consent until 24 months (2 years) post A2B694 infusion\|Phase 1: Recommended Phase 2 Dose (RP2D), The RP2D will be identified utilizing a BOIN study design in addition to considering safety and biomarker analysis., 21 days post A2B694 infusion\|Phase 2: The Overall Response Rate (ORR) for patients, The ORR will be evaluated per RECIST v1.1 and assessed by independent central review., 24 months post A2B694 infusion | PHASE1\|PHASE2 | 230 |
|  | NCT04404595 | Claudin18.2 CAR-T (CT041) in Patients With Gastric, Pancreatic Cancer, or Other Specified Digestive Cancers | Gastric Cancer\|Pancreatic Cancer | BIOLOGICAL: CT041 | Phase 1b: Incidence of Treatment Related adverse events (AEs), Incidence of Treatment Related AEs, AEs of special interest and serious adverse events (SAEs), up to 18 mos\|Phase 1b: Identification of Maximum Tolerated Dose (MTD) & incidence of Dose-limiting Toxicities (DLTs), Incidence of dose-limiting toxicities (DLTs), day 1 - day 28\|Phase 2: Objective Response Rate (ORR) per independent central read, Rate of subjects experiencing \>/= to PR per RECIST 1.1 as determined by IRC assessment, up to 18 mos | PHASE1\|PHASE2 | 110 |
|  | NCT05538195 | Safety and Efficacy of CEA-targeted CAR-T for CEA-positive Advanced Malignant Solid Tumors | Gastric Cancer\|Colon Cancer\|Rectal Cancer\|Esophageal Cancer\|Pancreatic Cancer | BIOLOGICAL: CEA-targeted CAR-T cells\|BIOLOGICAL: CEA-targeted CAR-T cells | To evaluate the safety of CAR-T cell preparations in the treatment of CEA-positive advanced malignancies [Safety and Tolerability], The incidence of adverse events after CEA CAR-T cell infusion was assessed by the National Cancer Institute's Common Terminology Criteria for Adverse Events (CTCAE, version 5.0), 1 month\|Obtained the recommended dose and infusion regimen of CAR-T cells for the treatment of patients with CEA-positive advanced malignancies[Safety and Tolerability], Dose-limiting toxicity after CEA CAR-T cell infusion, 28 days | PHASE1\|PHASE2 | 60 |
|  | NCT05795595 | A Safety and Efficacy Study Evaluating CTX131 in Adult Subjects With Relapsed or Refractory Solid Tumors | Clear Cell Renal Cell Carcinoma\|Cervical Carcinoma\|Esophageal Carcinoma\|Pancreatic Adenocarcinoma\|Malignant Pleural Mesothelioma | BIOLOGICAL: CTX131 | Phase 1 (Dose Escalation): Incidence of adverse events, Defined as dose-limiting toxicities, From CTX131 infusion up to 28 days post-infusion\|Phase 2 (Cohort Expansion): Objective response rate (ORR), ORR based on Independent Review Committee (IRC) assessment, defined as the proportion of subjects who have achieved a best overall response of CR or PR according to appropriate response evaluation criteria for each disease histology, From CTX131 infusion up to 60 months post-infusion | PHASE1\|PHASE2 | 250 |
|  | NCT05239143 | P-MUC1C-ALLO1 Allogeneic CAR-T Cells in the Treatment of Subjects With Advanced or Metastatic Solid Tumors | Breast Cancer\|Ovarian Cancer\|Non Small Cell Lung Cancer\|Colorectal Cancer\|Pancreatic Cancer\|Renal Cell Carcinoma\|Nasopharyngeal Cancer\|Head and Neck Squamous Cell Carcinoma\|Gastric Cancer | BIOLOGICAL: P-MUC1C-ALLO1 CAR-T cells\|DRUG: Rimiducid | Determine the maximum tolerated dose (MTD) and/or recommended phase 2 dose (RP2D) of P-MUC1C-ALLO1, Number of subjects with a dose limiting toxicity (DLT), Baseline through Day 28\|Evaluate the overall safety and tolerability profile of P-MUC1C-ALLO1, Frequency and severity of adverse events, Baseline through 15 years\|Evaluate the preliminary efficacy of P-MUC1C-ALLO1, According to the Response Evaluation Criteria in Solid Tumors (RECIST) version 1.1, secondarily Immune Response Evaluation Criteria in Solid Tumors (iRECIST): Overall Response Rate (ORR), Baseline through 15 years | PHASE1 | 100 |
|  | NCT04348643 | Safety and Efficacy of CEA-Targeted CAR-T Therapy for Relapsed/Refractory CEA+ Cancer | Solid Tumor\|Lung Cancer\|Colorectal Cancer\|Liver Cancer\|Pancreatic Cancer\|Gastric Cancer\|Breast Cancer | BIOLOGICAL: CEA CAR-T cells | Adverse events that related to treatment, Therapy-related adverse events will be recorded and assessed according to the National Cancer Institute's Common Terminology Criteria for Adverse Events (CTCAE, Version 5.0), 2 years | PHASE1\|PHASE2 | 40 |
|  | NCT04966143 | Clinical Study of LY011 in the Treatment of Advanced Pancreatic Cancer | Pancreatic Cancer | BIOLOGICAL: LY011 | Overall Response rateï¼ˆORRï¼‰, the proportion of patients with best overall response of complete response (CR) or partial response (PR), as per local investigatorÂ´s assessment and according to Response Evaluation Criteria in Solid Tumors (RECIST) version 1.1 criteria, 1month | EARLY_PHASE1 | 30 |
|  | NCT03302403 | Clinical Study of Redirected Autologous T Cells With a Chimeric Antigen Receptor in Patients With Malignant Tumors | B Cell Lymphoma\|B Cell Leukemia\|Myeloma\|Hepatocellular Carcinoma\|Pancreatic Carcinoma\|Adenocarcinoma of Esophagogastric Junction | GENETIC: CAR-CD19 T cell\|GENETIC: CAR-BCMA T cell\|GENETIC: CAR-GPC3 T cell\|GENETIC: CAR-CLD18 T cell\|DRUG: Fludarabine\|DRUG: Cyclophosphamide | Number of participants with CRA T-related adverse events as assessed by CTCAE v4.03, Number of participants with study related adverse events which are defined as laboratory toxicities and clinical events that are possible, likely or definitely related to study treatment at any time from the infusion until week 24, including infusion related toxicity and any toxicity possibly related to CAR T cells., 24 weeks | NA | 18 |
|  | NCT02713984 | A Clinical Research of CAR T Cells Targeting HER2 Positive Cancer | Breast Cancer\|Ovarian Cancer\|Lung Cancer\|Gastric Cancer\|Colorectal Cancer\|Glioma\|Pancreatic Cancer | BIOLOGICAL: Anti-HER2 CAR-T | Determine the toxicity profile of the HER2 targeted CAR T cells with Common Toxicity Criteria for Adverse Effects (CTCAE) version 4.0., Observe and handle the toxicity profile of the HER2 targeted CAR T cells with Common Toxicity Criteria for Adverse Effects (CTCAE) version 4.0., 3 years | PHASE1\|PHASE2 | 0 |
|  | NCT06006390 | CEA Targeting Chimeric Antigen Receptor T Lymphocytes (CAR-T) in the Treatment of CEA Positive Advanced Solid Tumors | Gastric Cancer\|Colon Cancer\|Rectal Cancer\|Esophageal Cancer\|Pancreas Cancer\|Lung Cancer\|Breast Cancer | BIOLOGICAL: CEA-targeted CAR-T cells\|BIOLOGICAL: CEA-targeted CAR-T cells | To evaluate the safety of CAR-T cell preparations in the treatment of CEA-positive advanced malignancies [Safety and Tolerability], The incidence of adverse events after CEA CAR-T cell infusion was assessed by the National Cancer Institute's Common Terminology Criteria for Adverse Events (CTCAE, version 5.0), 1 month\|Obtained the recommended dose and infusion regimen of CAR-T cells for the treatment of patients with CEA-positive advanced malignancies[Safety and Tolerability], Dose-limiting toxicity after CEA CAR-T cell infusion, 28 days | PHASE1\|PHASE2 | 60 |
|  | NCT05028933 | IMC001 for Clinical Research on Advanced Digestive System Malignancies | Advanced Hepatocellular Carcinoma\|Advanced Colorectal Cancer\|Advanced Gastric Cancer\|Advanced Pancreatic Cancer | DRUG: EPCAM CAR-T | Dose limited toxicity ï¼ˆDLTï¼‰, Safety, 28 days\|Maximum Tolerated Doseï¼ˆMTDï¼‰, Tolerability evaluation, 28 days\|Adverse Event(AE), Incidence rate, 28 days | PHASE1 | 48 |
|  | NCT05057715 | huCART-meso + VCN-01 in Pancreatic and Ovarian Cancer | Pancreatic Cancer\|Serous Ovarian Cancer | BIOLOGICAL: VCN-01\|BIOLOGICAL: huCART-meso Cells | Type, frequency, severity, and attribution of AEs/SAEs as assessed by CTCAE v 5.0, 2 years\|Occurrence of dose-limiting toxicities., 2 years | PHASE1 | 12 |
|  | NCT05396300 | A Clinical Study of CEA-targeted CAR-T in the Treatment of CEA-positive Advanced Malignant Solid Tumors | Colorectal Cancer\|Esophageal Cancer\|Stomach Cancer\|Pancreatic Cancer\|Metastatic Tumor\|Recurrent Cancer | BIOLOGICAL: CEA CAR-T cells | Incidence of Adverse events after CEA-CAR-T cells infusion [Safety and Tolerability], Therapy-related adverse events were recorded and assessed according to the National Cancer Institute's Common Terminology Criteria for Adverse Events (CTCAE, Version 5.0), 28 days\|Obtain the maximum tolerated dose of CEA-CAR-T cells[Safety and Tolerability], Dose-limiting toxicity after cell infusion, 28 days | PHASE1 | 60 |
|  | NCT01897415 | Autologous Redirected RNA Meso CAR T Cells for Pancreatic Cancer | Subjects With Metastatic Pancreatic Ductal Adenocarcinoma (PDA) | BIOLOGICAL: Autologous T cells transfected with chimeric anti-mesothelin immunoreceptor SS1 | Number of Adverse Events, Day 28 | PHASE1 | 16 |
|  | NCT05620732 | Treatment of Advanced Malignant Solid Tumors With Claudin18.2CAR-T | Advanced Pancreatic Carcinoma\|Advanced Gastric Carcinoma | BIOLOGICAL: Claudin18.2 CAR-T cells | AE/SAE, adverse events/ severe adverse events, From date of infusion to 30 days after infusion | NA | 20 |
|  | NCT05277987 | An Evaluation Trial About Anti-claudin18.2 the Specificity of Chimeric Antigen Receptor T Cells in the Advanced Gastric / Esophagogastric Junction Adenocarcinoma and Pancreatic Cancer Subjects | CAR T-Cell Therapy | DRUG: HEC-016(0.5Ã—10^6 CAR-T Cells)\|DRUG: HEC-016(0.5Ã—10^6.5 CAR-T Cells)\|DRUG: HEC-016(0.5Ã—10^7 CAR-T Cells) | DLT, Dose limiting toxicity, Within 28 days after the first infusion | EARLY_PHASE1 | 18 |
|  | NCT03740256 | Binary Oncolytic Adenovirus in Combination With HER2-Specific Autologous CAR VST, Advanced HER2 Positive Solid Tumors | Bladder Cancer\|Head and Neck Squamous Cell Carcinoma\|Cancer of the Salivary Gland\|Lung Cancer\|Breast Cancer\|Gastric Cancer\|Esophageal Cancer\|Colorectal Cancer\|Pancreatic Adenocarcinoma\|Solid Tumor | BIOLOGICAL: CAdVEC | Number of patients with dose limiting toxicity (DLT) by CTCAE 5.0, Incidence of dose limiting toxicities (DLT) of CAdVEC intratumoral injection in combination with HER2.CAR AdVST cells in patients with advanced refractory HER2 positive solid tumors., 6 weeks after the HER2.CAR AdVST infusion or 6 weeks + 3 days after the CAdVEC injection. | PHASE1 | 45 |
|  | NCT05605197 | U87 CART in Treatment of Advanced Solid Tumor | Pancreatic Cancer\|Solid Tumor, Adult | DRUG: U87 CAR-T | Incidence of Adverse events after U87 CAR-T cells infusion [Safety and Tolerability], Therapy-related adverse events were recorded and assessed according to the National Cancer Institute's Common Terminology Criteria for Adverse Events (CTCAE, Version 5.0) Dose-limiting toxicity after U87 CAR-T cells infusion., 28 days post administration of CAR-T-cells | PHASE1 | 12 |
|  | NCT03890198 | A Phase 1 Study of LCAR-C182A Cells in the Treatment of Advanced Gastric Cancer and Pancreatic Ductal Adenocarcinoma | Gastric Cancer\|Pancreatic Ductal Adenocarcinoma | BIOLOGICAL: LCAR-C182A cells | Number of Participants With Adverse Events, An adverse event is any untoward medical event that occurs in a participant administered an investigational product,and it does not necessarily indicate only events with clear causal relationship with the relevant investigational product., 90 days post infusion\|MTDï¼‰/ RP2D regimen finding, Maximum tolerated dose (MTD) and recommended Phase 2 dose (RP2D), 90 days post infusion\|Transgene Levels of LCAR-C182A CAR-T Cells, Transgene Levels of LCAR-C182A CAR-T Cells using sensitive assay methods will be assessed, 2 years post infusion\|Chimeric Antigen Receptor T (CAR-T) Positive Cell Concentration, Venous blood samples will be collected for measurement of CAR-T positive cellular concentration, 2 years post infusion\|Systemic Cytokine Concentrations, Serum cytokine concentrations such as IL-2, IL-6, IL-8, 1L-10, TNF-Î±, IFN-Î³ will be measured for biomarker assessment, 2 years post infusion | EARLY_PHASE1 | 2 |
|  | NCT02465983 | Pilot Study of Autologous T-cells in Patients With Metastatic Pancreatic Cancer | Pancreatic Cancer | BIOLOGICAL: CART-meso-19 T cells\|DRUG: Cyclophosphamide | Safety of IV administration of CART-meso-19 with cyclophosphamide as lymphodepleting chemotherapy in patients with pancreatic cancer using the NCI CTCAE v4.03 criteria, 24 months | PHASE1 | 4 |
|  | NCT03638193 | Study of Autologous T-cells in Patients With Metastatic Pancreatic Cancer | Pancreatic Cancer | BIOLOGICAL: CART-meso cells | Safety of CART-meso infusion: number of adverse events, Number of Adverse Events evaluated with NCI CTC AE, version 4.0\[Safety evaluation\], 60 months | NA | 10 |
|  | NCT02580747 | Treatment of Relapsed and/or Chemotherapy Refractory Advanced Malignancies by CART-meso | Malignant Mesothelioma\|Pancreatic Cancer\|Ovarian Tumor\|Triple Negative Breast Cancer\|Endometrial Cancer\|Other Mesothelin Positive Tumors | BIOLOGICAL: anti-meso-CAR vector transduced T cells | Occurrence of Study related adverse events, defined as \>= Grade 3 signs/symptoms, laboratory toxicities, and clinical, Until week 24 | PHASE1 | 20 |
|  | NCT05143151 | CD276-targeted Chimeric Antigen Receptor T Cells in Treatment With Advanced Pancreatic Cancer | Advanced Pancreatic Carcinoma | BIOLOGICAL: CD276 CAR-T cells | Objective response rate (ORR), up to 1 year | PHASE1\|PHASE2 | 10 |
|  | NCT02959151 | A Study of Chimeric Antigen Receptor T Cells Combined With Interventional Therapy in Advanced Liver Malignancy | Carcinoma, Hepatocellular\|Pancreatic Cancer Metastatic\|Colorectal Cancer Metastatic | DRUG: CAR-T cell | Number of patients with adverse event, adverse event is evaluated with CTCAE, version 4.0, 6 weeks | PHASE1\|PHASE2 | 20 |

**Supplementary Table S2: Gene Ontology enriched terms of Mesothelin (MSLN)**

| **Enriched Term** | **Enriched term ID** | **Term description** | **Strength** | **FDR** | **Matching proteins in the network** |
| --- | --- | --- | --- | --- | --- |
| Cellular component | GO:0005886 | Plasma membrane | 0.55 | 0.0018 | CEACAM5,FOLH1,ERBB2,EGFR,PSCA,CD247,MSLN,FOLR1,MUC16,TNFRSF9,MUC1 |
|  | GO:0009986 | Cell surface | 1.08 | 0.0018 | CEACAM5,FOLH1,EGFR,MSLN,FOLR1,TNFRSF9 |
|  | GO:0031224 | Intrinsic component of membrane | 0.53 | 0.0018 | CEACAM5,FOLH1,ERBB2,EGFR,PSCA,CD247,MSLN,FOLR1,MUC16,TNFRSF9,MUC1 |
|  | GO:0031225 | Anchored component of membrane | 1.62 | 0.0018 | CEACAM5,PSCA,MSLN,FOLR1 |
|  | GO:0016323 | Basolateral plasma membrane | 1.5 | 0.0019 | CEACAM5,ERBB2,EGFR,FOLR1 |
|  | GO:0031226 | Intrinsic component of plasma membrane | 0.85 | 0.0028 | CEACAM5,FOLH1,ERBB2,EGFR,FOLR1,TNFRSF9,MUC1 |
|  | GO:0044214 | Spanning component of plasma membrane | 2.44 | 0.0061 | ERBB2,EGFR |
|  | GO:0016324 | Apical plasma membrane | 1.29 | 0.0068 | CEACAM5,ERBB2,FOLR1,MUC1 |
|  | GO:0005615 | Extracellular space | 0.64 | 0.0096 | CEACAM5,FOLH1,EGFR,PSCA,MSLN,FOLR1,MUC16,MUC1 |
|  | GO:0005794 | Golgi apparatus | 0.81 | 0.0152 | EGFR,CD247,MSLN,FOLR1,MUC16,MUC1 |
|  | GO:0005887 | Integral component of plasma membrane | 0.8 | 0.0171 | CEACAM5,FOLH1,ERBB2,EGFR,TNFRSF9,MUC1 |
|  | GO:0031982 | Vesicle | 0.56 | 0.0294 | CEACAM5,FOLH1,ERBB2,EGFR,PSCA,FOLR1,MUC16,MUC1 |
|  | GO:0098590 | Plasma membrane region | 0.86 | 0.0376 | CEACAM5,ERBB2,EGFR,FOLR1,MUC1 |
|  | GO:0016021 | Integral component of membrane | 0.45 | 0.0402 | CEACAM5,FOLH1,ERBB2,EGFR,CD247,FOLR1,MUC16,TNFRSF9,MUC1 |
|  | GO:0070062 | Extracellular exosome | 0.71 | 0.0402 | CEACAM5,FOLH1,PSCA,FOLR1,MUC16,MUC1 |
| Subcellular localization | GOCC:0031226 | Intrinsic component of plasma membrane | 1.17 | 0.00019 | CEACAM5,FOLH1,ERBB2,EGFR,FOLR1,TNFRSF9,MUC1 |
|  | GOCC:0005886 | Plasma membrane | 0.7 | 0.00037 | CEACAM5,FOLH1,ERBB2,EGFR,CD247,MSLN,FOLR1,MUC16,TNFRSF9,MUC1 |
|  | GOCC:0009925 | Basal plasma membrane | 1.76 | 0.00041 | CEACAM5,ERBB2,EGFR,FOLR1 |
|  | GOCC:0005576 | Extracellular region | 0.84 | 0.00055 | CEACAM5,FOLH1,EGFR,PSCA,MSLN,FOLR1,MUC16,MUC1 |
|  | GOCC:0005887 | Integral component of plasma membrane | 1.13 | 0.00055 | CEACAM5,FOLH1,ERBB2,EGFR,TNFRSF9,MUC1 |
|  | GOCC:0070062 | Extracellular exosome | 1.32 | 0.00055 | CEACAM5,FOLH1,FOLR1,MUC16,MUC1 |
|  | GOCC:0065010 | Extracellular membrane-bounded organelle | 1.28 | 0.00077 | CEACAM5,FOLH1,FOLR1,MUC16,MUC1 |
|  | GOCC:0005615 | Extracellular space | 1.02 | 0.0012 | CEACAM5,FOLH1,EGFR,FOLR1,MUC16,MUC1 |
|  | GOCC:0031982 | Vesicle | 0.77 | 0.0052 | CEACAM5,FOLH1,ERBB2,EGFR,FOLR1,MUC16,MUC1 |
|  | GOCC:0044214 | Spanning component of plasma membrane | 2.41 | 0.0052 | ERBB2,EGFR |
|  | GOCC:0043235 | Receptor complex | 1.23 | 0.0078 | ERBB2,EGFR,CD247,TNFRSF9 |
|  | GOCC:0009986 | Cell surface | 1.21 | 0.008 | CEACAM5,FOLH1,EGFR,FOLR1 |
|  | GOCC:0045177 | Apical part of cell | 1.36 | 0.0286 | CEACAM5,FOLR1,MUC1 |
|  | GOCC:0031225 | Anchored component of membrane | 1.86 | 0.0356 | CEACAM5,FOLR1 |
| Tissue expression | BTO:0000093 | MCF-7 cell | 2.51 | 0.0103 | ERBB2,EGFR |
|  | BTO:0000167 | Adenocarcinoma cell line | 1.7 | 0.0103 | CEACAM5,ERBB2,EGFR |
|  | BTO:0000815 | MDA-MB-231 cell | 2.6 | 0.0103 | ERBB2,EGFR |
|  | BTO:0001491 | Viscus | 0.52 | 0.0103 | CEACAM5,FOLH1,ERBB2,EGFR,PSCA,CD247,MSLN,FOLR1,TNFRSF9,MUC1 |
|  | BTO:0001570 | MDA-MB-468 cell | 2.95 | 0.0103 | ERBB2,EGFR |
|  | BTO:0001791 | Pleura | 2.86 | 0.0103 | CEACAM5,MSLN |
|  | BTO:0001932 | BT-474 cell | 2.95 | 0.0103 | ERBB2,EGFR |
|  | BTO:0002419 | SK-BR-3 cell | 2.86 | 0.0103 | ERBB2,EGFR |
|  | BTO:0000811 | Ovary cancer cell line | 2.05 | 0.0273 | ERBB2,FOLR1 |
|  | BTO:0001616 | Colorectal cancer cell line | 2.02 | 0.0286 | CEACAM5,EGFR |
| Disease-gene associations | DOID:305 | Carcinoma | 1.54 | 3.04E-05 | CEACAM5,FOLH1,ERBB2,EGFR,PSCA,MSLN |
|  | DOID:850 | Lung disease | 1.7 | 6.63E-05 | FOLH1,ERBB2,EGFR,MSLN,MUC1 |
|  | DOID:299 | Adenocarcinoma | 1.78 | 0.00035 | CEACAM5,FOLH1,ERBB2,EGFR |
|  | DOID:1793 | Pancreatic cancer | 2.27 | 0.00042 | CEACAM5,ERBB2,EGFR |
|  | DOID:10534 | Stomach cancer | 2.24 | 0.00044 | ERBB2,EGFR,PSCA |
|  | DOID:9256 | Colorectal cancer | 2.23 | 0.00044 | CEACAM5,ERBB2,EGFR |
|  | DOID:0050686 | Organ system cancer | 1.15 | 0.00059 | CEACAM5,FOLH1,ERBB2,EGFR,PSCA,MSLN |
|  | DOID:3119 | Gastrointestinal system cancer | 1.65 | 0.00062 | CEACAM5,ERBB2,EGFR,PSCA |
|  | DOID:3082 | Interstitial lung disease | 2.12 | 0.00065 | FOLH1,MSLN,MUC1 |
|  | DOID:0080374 | Gastroesophageal cancer | 2.95 | 0.0013 | ERBB2,EGFR |
|  | DOID:0050615 | Respiratory system cancer | 1.82 | 0.0028 | ERBB2,EGFR,MSLN |
|  | DOID:0080199 | Colorectal carcinoma | 2.6 | 0.0034 | CEACAM5,EGFR |
|  | DOID:4074 | Pancreatic adenocarcinoma | 2.6 | 0.0034 | CEACAM5,EGFR |
|  | DOID:10316 | Pneumoconiosis | 2.47 | 0.0049 | FOLH1,MSLN |
|  | DOID:8719 | In situ carcinoma | 2.44 | 0.0054 | ERBB2,EGFR |
|  | DOID:1795 | Tumor of exocrine pancreas | 2.32 | 0.0079 | CEACAM5,EGFR |
|  | DOID:219 | Colon cancer | 2.32 | 0.0079 | CEACAM5,EGFR |
|  | DOID:5517 | Stomach carcinoma | 2.28 | 0.0091 | ERBB2,PSCA |
|  | DOID:193 | Reproductive organ cancer | 1.51 | 0.0147 | CEACAM5,FOLH1,ERBB2 |
|  | DOID:3118 | Hepatobiliary disease | 1.5 | 0.0147 | CEACAM5,ERBB2,EGFR |
|  | DOID:3908 | Lung non-small cell carcinoma | 2.14 | 0.0147 | ERBB2,EGFR |
|  | DOID:7 | Disease of anatomical entity | 0.53 | 0.0147 | CEACAM5,FOLH1,ERBB2,EGFR,PSCA,CD247,MSLN,FOLR1,MUC1 |
|  | DOID:3459 | Breast carcinoma | 2.09 | 0.0171 | ERBB2,EGFR |
|  | DOID:1383 | Sweat gland disease | 2.05 | 0.0201 | CEACAM5,ERBB2 |
|  | DOID:4947 | Cholangiocarcinoma | 1.95 | 0.0277 | CEACAM5,EGFR |
|  | DOID:1749 | Squamous cell carcinoma | 1.85 | 0.0384 | EGFR,MSLN |

**Supplementary Table S3: Gene Ontology enriched terms of Carcinoma embryonic antigen (CEA/CEACAM5)**

| **Enriched Term** | **Enriched Term ID** | **Term description** | **Strength** | **FDR** | **Matching proteins in the network** |
| --- | --- | --- | --- | --- | --- |
| Cellular component | GO:0005576 | Extracellular region | 0.63 | 0.0034 | CEACAM1,CEACAM5,KLK3,CD209,KRT7,KRT19,MSLN,AFP,ENO2,MUC1 |
|  | GO:0005615 | Extracellular space | 0.7 | 0.0037 | CEACAM1,CEACAM5,KLK3,KRT7,KRT19,MSLN,AFP,ENO2,MUC1 |
|  | GO:0009986 | Cell surface | 1 | 0.0238 | CEACAM1,CEACAM5,CD209,MSLN,ENO2 |
|  | GO:0070062 | Extracellular exosome | 0.78 | 0.0238 | CEACAM1,CEACAM5,KLK3,KRT7,KRT19,ENO2,MUC1 |
|  | GO:0005882 | Intermediate filament | 1.41 | 0.0473 | KRT20,KRT7,KRT19 |
| Subcellular localization | GOCC:0043230 | Extracellular organelle | 1.38 | 6.85E-06 | CEACAM1,CEACAM5,KLK3,KRT7,KRT19,ENO2,MUC1 |
|  | GOCC:0005576 | Extracellular region | 0.89 | 8.58E-05 | CEACAM1,CEACAM5,KLK3,KRT7,KRT19,MSLN,AFP,ENO2,MUC1 |
|  | GOCC:0005882 | Intermediate filament | 1.95 | 8.58E-05 | KRT20,KRT7,KRT19,ENO2 |
|  | GOCC:0065010 | Extracellular membrane-bounded organelle | 1.36 | 8.58E-05 | CEACAM5,KLK3,KRT7,KRT19,ENO2,MUC1 |
|  | GOCC:1903561 | Extracellular vesicle | 1.33 | 8.58E-05 | CEACAM5,KLK3,KRT7,KRT19,ENO2,MUC1 |
|  | GOCC:0005615 | Extracellular space | 1.09 | 0.00011 | CEACAM5,KLK3,KRT7,KRT19,AFP,ENO2,MUC1 |
|  | GOCC:0070062 | Extracellular exosome | 1.32 | 0.0006 | CEACAM5,KLK3,KRT19,ENO2,MUC1 |
|  | GOCC:0097570 | Cyst wall | 3.08 | 0.00072 | CEACAM5,KRT7 |
|  | GOCC:0031982 | Vesicle | 0.77 | 0.0088 | CEACAM1,CEACAM5,KLK3,KRT7,KRT19,ENO2,MUC1 |
|  | GOCC:0071944 | Cell periphery | 0.57 | 0.0319 | CEACAM1,CEACAM5,CD209,KRT7,KRT19,MSLN,AFP,MUC1 |
|  | GOCC:0045177 | Apical part of cell | 1.36 | 0.0394 | CEACAM1,CEACAM5,MUC1 |
|  | GOCC:0030312 | External encapsulating structure | 1.32 | 0.0479 | CEACAM5,KRT7,AFP |
| Tissue expression | BTO:0001006 | Pelvis | 3.16 | 1.60E-08 | KRT20,CEACAM5,KLK3,KRT7 |
|  | BTO:0001190 | Root nodule | 2.89 | 1.88E-05 | CEACAM5,KRT7,AFP |
|  | BTO:0000122 | Bile duct | 2.69 | 3.79E-05 | KRT7,KRT19,AFP |
|  | BTO:0002840 | Bile ductule | 3.25 | 0.001 | KRT7,KRT19 |
|  | BTO:0004270 | Adult liver stem cell | 3.08 | 0.0011 | KRT19,AFP |
|  | BTO:0000284 | Organism form | 0.75 | 0.0021 | KRT20,CEACAM5,CD209,KRT7,KRT19,MSLN,AFP,ENO2 |
|  | BTO:0000417 | Bile duct epithelium | 2.86 | 0.0021 | KRT7,KRT19 |
|  | BTO:0000511 | Gastrointestinal tract | 0.84 | 0.0021 | KRT20,CEACAM5,KLK3,KRT19,AFP,ENO2,MUC1 |
|  | BTO:0001493 | Trunk | 1.03 | 0.0021 | KRT20,CEACAM5,KLK3,KRT7,KRT19,MUC1 |
|  | BTO:0001791 | Pleura | 2.86 | 0.0021 | CEACAM5,MSLN |
|  | BTO:0001893 | Ring stage | 2.86 | 0.0021 | KRT20,KRT7 |
|  | BTO:0002422 | Mesothelium | 2.86 | 0.0021 | KRT7,MSLN |
|  | BTO:0001491 | Viscus | 0.52 | 0.0025 | CEACAM1,KRT20,CEACAM5,KLK3,KRT7,KRT19,MSLN,AFP,ENO2,MUC1 |
|  | BTO:0000416 | Epithelium | 1.11 | 0.0026 | KRT20,KLK3,KRT7,KRT19,MSLN |
|  | BTO:0000763 | Lung | 0.89 | 0.0048 | CEACAM5,KRT7,KRT19,MSLN,AFP,MUC1 |
|  | BTO:0000234 | Vein | 2.19 | 0.0085 | CEACAM5,AFP |
|  | BTO:0000570 | Hematopoietic system | 0.66 | 0.0186 | CEACAM1,KLK3,KRT7,KRT19,MSLN,AFP,ENO2 |
|  | BTO:0000176 | Carcinoma cell | 1.07 | 0.0232 | CEACAM1,KRT7,MSLN,MUC1 |
|  | BTO:0000345 | Digestive gland | 0.64 | 0.0232 | CEACAM1,CEACAM5,KRT7,KRT19,MSLN,AFP,ENO2 |
|  | BTO:0000414 | Epithelial cell | 1.91 | 0.0232 | KRT7,KRT19 |
|  | BTO:0000988 | Pancreas | 1.06 | 0.0232 | CEACAM5,KRT7,KRT19,MSLN |
|  | BTO:0000449 | Fetus | 0.83 | 0.0303 | CD209,KRT7,KRT19,AFP,ENO2 |
|  | BTO:0000586 | Colonic cancer cell | 1.28 | 0.0303 | CEACAM1,KRT7,KRT19 |
|  | BTO:0000634 | Integument | 0.71 | 0.0303 | CEACAM1,KRT20,KLK3,KRT7,KRT19,MSLN |
|  | BTO:0000759 | Liver | 0.7 | 0.0303 | CEACAM1,CEACAM5,KRT7,KRT19,AFP,ENO2 |
|  | BTO:0000794 | Pancreatic cancer cell line | 1.76 | 0.034 | MSLN,MUC1 |
|  | BTO:0001158 | Rectum | 1.74 | 0.0355 | KRT20,KLK3 |
|  | BTO:0000174 | Embryonic structure | 0.66 | 0.0462 | CD209,KRT7,KRT19,MSLN,AFP,ENO2 |
| Disease-gene associations | DOID:305 | Carcinoma | 1.67 | 2.83E-09 | KRT20,CEACAM5,KLK3,KRT7,KRT19,MSLN,AFP,ENO2 |
|  | DOID:299 | Adenocarcinoma | 1.96 | 3.97E-08 | KRT20,CEACAM5,KLK3,KRT7,KRT19,AFP |
|  | DOID:2394 | Ovarian cancer | 2.21 | 1.15E-07 | KRT20,CEACAM5,KRT7,AFP,ENO2 |
|  | DOID:3119 | Gastrointestinal system cancer | 1.82 | 1.39E-07 | KRT20,CEACAM5,KRT7,KRT19,AFP,ENO2 |
|  | DOID:193 | Reproductive organ cancer | 1.81 | 1.43E-07 | KRT20,CEACAM5,KLK3,KRT7,AFP,ENO2 |
|  | DOID:0050686 | Organ system cancer | 1.28 | 4.85E-07 | KRT20,CEACAM5,KLK3,KRT7,KRT19,MSLN,AFP,ENO2 |
|  | DOID:0060085 | Organ system benign neoplasm | 1.66 | 8.32E-07 | KRT20,CEACAM5,KLK3,KRT7,AFP,ENO2 |
|  | DOID:10534 | Stomach cancer | 2.36 | 1.08E-06 | KRT20,KRT7,AFP,ENO2 |
|  | DOID:2634 | Cystadenoma | 3.25 | 1.08E-06 | KRT20,CEACAM5,KRT7 |
|  | DOID:3493 | Signet ring cell adenocarcinoma | 3.13 | 1.48E-06 | KRT20,CEACAM5,KRT7 |
|  | DOID:77 | Gastrointestinal system disease | 1.34 | 1.69E-06 | KRT20,CEACAM5,KLK3,KRT7,KRT19,AFP,ENO2 |
|  | DOID:15 | Reproductive system disease | 1.53 | 2.16E-06 | KRT20,CEACAM5,KLK3,KRT7,AFP,ENO2 |
|  | DOID:4607 | Biliary tract cancer | 2.16 | 3.67E-06 | CEACAM5,KRT7,KRT19,AFP |
|  | DOID:4928 | Intrahepatic cholangiocarcinoma | 2.89 | 3.67E-06 | CEACAM5,KRT19,AFP |
|  | DOID:0060084 | Cell type benign neoplasm | 1.71 | 5.24E-06 | KRT20,CEACAM5,KLK3,KRT7,AFP |
|  | DOID:4138 | Bile duct disease | 1.99 | 1.23E-05 | CEACAM5,KRT7,KRT19,AFP |
|  | DOID:5517 | Stomach carcinoma | 2.45 | 3.25E-05 | KRT20,KRT7,ENO2 |
|  | DOID:2152 | Ovary epithelial cancer | 2.39 | 4.68E-05 | KRT20,CEACAM5,KRT7 |
|  | DOID:3165 | Skin benign neoplasm | 2.2 | 0.00015 | CEACAM5,KRT7,ENO2 |
|  | DOID:0050624 | Gastrointestinal system benign neoplasm | 2.12 | 0.00023 | CEACAM5,KRT7,AFP |
|  | DOID:12192 | Sigmoid colon cancer | 3.25 | 0.00023 | KRT20,CEACAM5 |
|  | DOID:4023 | Linitis plastica | 3.25 | 0.00023 | KRT20,KRT7 |
|  | DOID:4468 | Clear cell adenocarcinoma | 3.25 | 0.00023 | KRT20,KRT7 |
|  | DOID:9597 | Krukenberg carcinoma | 3.25 | 0.00023 | KRT20,KRT7 |
|  | DOID:2098 | Vulva adenocarcinoma | 3.08 | 0.0003 | KRT20,KRT7 |
|  | DOID:2658 | Dermoid cyst | 3.08 | 0.0003 | KRT7,AFP |
|  | DOID:3450 | Cutaneous Pagets disease | 3.08 | 0.0003 | KRT20,KRT7 |
|  | DOID:3559 | Pseudomyxoma peritonei | 3.08 | 0.0003 | KRT20,KRT7 |
|  | DOID:3711 | Bladder adenocarcinoma | 3.08 | 0.0003 | KRT20,KRT7 |
|  | DOID:6067 | Ovarian mucinous neoplasm | 3.08 | 0.0003 | KRT20,KRT7 |
|  | DOID:10155 | Intestinal cancer | 1.96 | 0.00041 | KRT20,CEACAM5,KRT7 |
|  | DOID:365 | Bladder disease | 1.96 | 0.00041 | KRT20,KLK3,KRT7 |
|  | DOID:5295 | Intestinal disease | 1.52 | 0.00041 | KRT20,CEACAM5,KLK3,KRT7 |
|  | DOID:4159 | Skin cancer | 1.93 | 0.00048 | KRT20,KRT7,ENO2 |
|  | DOID:1800 | Neuroendocrine carcinoma | 2.78 | 0.00061 | KRT20,ENO2 |
|  | DOID:4948 | Gallbladder carcinoma | 2.78 | 0.00061 | CEACAM5,KRT7 |
|  | DOID:3856 | Male reproductive organ cancer | 1.86 | 0.00069 | KRT20,KLK3,AFP |
|  | DOID:2156 | Ovarian germ cell cancer | 2.71 | 0.00072 | AFP,ENO2 |
|  | DOID:3111 | Cystadenocarcinoma | 2.71 | 0.00072 | CEACAM5,KRT7 |
|  | DOID:0080199 | Colorectal carcinoma | 2.6 | 0.001 | KRT20,CEACAM5 |
|  | DOID:170 | Endocrine gland cancer | 1.76 | 0.0011 | CEACAM5,AFP,ENO2 |
|  | DOID:3117 | Hepatobiliary benign neoplasm | 2.51 | 0.0014 | KRT7,AFP |
|  | DOID:2664 | Sweat gland benign neoplasm | 2.47 | 0.0016 | CEACAM5,KRT7 |
|  | DOID:3030 | Mucinous adenocarcinoma | 2.47 | 0.0016 | KRT20,KRT7 |
|  | DOID:4905 | Pancreatic carcinoma | 2.38 | 0.0022 | CEACAM5,AFP |
|  | DOID:1795 | Tumor of exocrine pancreas | 2.32 | 0.0028 | CEACAM5,AFP |
|  | DOID:4 | Disease | 0.45 | 0.0047 | KRT20,CEACAM5,KLK3,CD209,KRT7,KRT19,MSLN,AFP,ENO2,MUC1 |
|  | DOID:7 | Disease of anatomical entity | 0.53 | 0.0055 | KRT20,CEACAM5,KLK3,KRT7,KRT19,MSLN,AFP,ENO2,MUC1 |
|  | DOID:850 | Lung disease | 1.48 | 0.0064 | MSLN,ENO2,MUC1 |
|  | DOID:37 | Skin disease | 1.14 | 0.0072 | KRT20,CEACAM5,KRT7,ENO2 |
|  | DOID:0050622 | Reproductive organ benign neoplasm | 2.05 | 0.0081 | KRT20,KLK3 |
|  | DOID:3082 | Interstitial lung disease | 1.94 | 0.0121 | MSLN,MUC1 |
|  | DOID:363 | Uterine cancer | 1.89 | 0.0148 | AFP,ENO2 |
|  | DOID:1319 | Brain cancer | 1.83 | 0.019 | AFP,ENO2 |
|  | DOID:0060089 | Endocrine organ benign neoplasm | 1.78 | 0.0229 | KRT7,AFP |
|  | DOID:1115 | Sarcoma | 1.69 | 0.0336 | KLK3,AFP |
|  | DOID:0050615 | Respiratory system cancer | 1.65 | 0.0404 | MSLN,ENO2 |

**Supplementary Table S4: Gene Ontology enriched terms of CD8A (CD8α)**

| **Enriched Term** | **Enriched Term ID** | **Term description** | **Strength** | **FDR** | **Matching proteins in the network** |
| --- | --- | --- | --- | --- | --- |
| Biological Process | GO:0042110 | T cell activation | 1.78 | 1.28E-13 | CD4,CD3D,CD8B,CD247,CD2,HLA-E,HLA-A,CD8A,LCK,B2M |
|  | GO:0002684 | Positive regulation of immune system process | 1.35 | 7.21E-12 | CD4,CD3D,CD8B,CD247,CD2,HLA-E,HLA-G,HLA-A,CD8A,LCK,B2M |
|  | GO:0050778 | Positive regulation of immune response | 1.55 | 7.21E-12 | CD4,CD3D,CD8B,CD247,HLA-E,HLA-G,HLA-A,CD8A,LCK,B2M |
|  | GO:0002250 | Adaptive immune response | 1.65 | 3.48E-11 | CD4,CD3D,CD8B,CD247,HLA-E,HLA-G,HLA-A,CD8A,B2M |
|  | GO:0006955 | Immune response | 1.17 | 2.53E-10 | CD4,CD3D,CD8B,CD247,CD2,HLA-E,HLA-G,HLA-A,CD8A,LCK,B2M |
|  | GO:0002768 | Immune response-regulating cell surface receptor signaling pathway | 1.74 | 1.42E-08 | CD3D,CD8B,CD247,HLA-G,HLA-A,CD8A,LCK |
|  | GO:0050852 | T cell receptor signaling pathway | 2.02 | 1.47E-08 | CD3D,CD8B,CD247,HLA-A,CD8A,LCK |
|  | GO:0050863 | Regulation of T cell activation | 1.52 | 3.00E-07 | CD4,CD2,HLA-E,HLA-G,HLA-A,LCK,B2M |
|  | GO:0002696 | Positive regulation of leukocyte activation | 1.51 | 3.26E-07 | CD4,CD2,HLA-E,HLA-G,HLA-A,LCK,B2M |
|  | GO:0002483 | Antigen processing and presentation of endogenous peptide antigen | 2.6 | 3.33E-07 | HLA-E,HLA-G,HLA-A,B2M |
|  | GO:0019882 | Antigen processing and presentation | 1.98 | 8.82E-07 | HLA-E,HLA-G,HLA-A,CD8A,B2M |
|  | GO:0002474 | Antigen processing and presentation of peptide antigen via MHC class I | 2.41 | 1.22E-06 | HLA-E,HLA-G,HLA-A,B2M |
|  | GO:0050870 | Positive regulation of T cell activation | 1.63 | 1.27E-06 | CD4,HLA-E,HLA-G,HLA-A,LCK,B2M |
|  | GO:0001914 | Regulation of T cell mediated cytotoxicity | 2.26 | 3.35E-06 | HLA-E,HLA-G,HLA-A,B2M |
|  | GO:0002428 | Antigen processing and presentation of peptide antigen via MHC class Ib | 2.95 | 5.15E-06 | HLA-E,HLA-G,B2M |
|  | GO:0042270 | Protection from natural killer cell mediated cytotoxicity | 2.95 | 5.15E-06 | HLA-E,HLA-G,HLA-A |
|  | GO:0002449 | Lymphocyte mediated immunity | 1.75 | 7.06E-06 | CD2,HLA-G,HLA-A,CD8A,B2M |
|  | GO:0030217 | T cell differentiation | 1.72 | 9.54E-06 | CD4,CD3D,CD8A,LCK,B2M |
|  | GO:0002822 | Regulation of adaptive immune response based on somatic recombination of immune receptors built from immunoglobulin superfamily domains | 1.7 | 1.16E-05 | CD4,HLA-E,HLA-G,HLA-A,B2M |
|  | GO:0002520 | Immune system development | 1.22 | 1.28E-05 | CD4,CD3D,HLA-E,HLA-G,CD8A,LCK,B2M |
|  | GO:0007166 | Cell surface receptor signaling pathway | 0.9 | 2.25E-05 | CD4,CD3D,CD8B,CD247,CD2,HLA-G,HLA-A,CD8A,LCK |
|  | GO:0001819 | Positive regulation of cytokine production | 1.35 | 3.04E-05 | CD4,CD2,HLA-E,HLA-G,HLA-A,B2M |
|  | GO:0002720 | Positive regulation of cytokine production involved in immune response | 1.95 | 3.26E-05 | HLA-E,HLA-G,HLA-A,B2M |
|  | GO:0046631 | Alpha-beta T cell activation | 1.87 | 6.06E-05 | CD3D,CD247,HLA-E,HLA-A |
|  | GO:0002824 | Positive regulation of adaptive immune response based on somatic recombination of immune receptors built from immunoglobulin superfamily domains | 1.81 | 9.18E-05 | CD4,HLA-E,HLA-A,B2M |
|  | GO:0002708 | Positive regulation of lymphocyte mediated immunity | 1.79 | 0.00011 | HLA-E,HLA-G,HLA-A,B2M |
|  | GO:0001916 | Positive regulation of T cell mediated cytotoxicity | 2.28 | 0.00014 | HLA-E,HLA-A,B2M |
|  | GO:0002456 | T cell mediated immunity | 2.21 | 0.00021 | HLA-A,CD8A,B2M |
|  | GO:0002478 | Antigen processing and presentation of exogenous peptide antigen | 2.14 | 0.00032 | HLA-E,HLA-A,B2M |
|  | GO:0002460 | Adaptive immune response based on somatic recombination of immune receptors built from immunoglobulin superfamily domains | 1.63 | 0.00038 | HLA-G,HLA-A,CD8A,B2M |
|  | GO:0006952 | Defense response | 0.95 | 0.00047 | CD4,CD2,HLA-E,HLA-G,HLA-A,LCK,B2M |
|  | GO:0001909 | Leukocyte mediated cytotoxicity | 1.98 | 0.00078 | CD2,HLA-A,B2M |
|  | GO:0002477 | Antigen processing and presentation of exogenous peptide antigen via MHC class Ib | 2.95 | 0.00078 | HLA-E,B2M |
|  | GO:2000566 | Positive regulation of CD8-positive, alpha-beta T cell proliferation | 2.95 | 0.00078 | HLA-E,HLA-A |
|  | GO:0002476 | Antigen processing and presentation of endogenous peptide antigen via MHC class Ib | 2.86 | 0.001 | HLA-E,HLA-G |
|  | GO:0019731 | Antibacterial humoral response | 1.92 | 0.0011 | HLA-E,HLA-A,B2M |
|  | GO:0098542 | Defense response to other organism | 1.04 | 0.0011 | CD4,CD2,HLA-E,HLA-A,LCK,B2M |
|  | GO:0002729 | Positive regulation of natural killer cell cytokine production | 2.65 | 0.0021 | HLA-E,HLA-G |
|  | GO:0042742 | Defense response to bacterium | 1.37 | 0.0028 | CD4,HLA-E,HLA-A,B2M |
|  | GO:0019885 | Antigen processing and presentation of endogenous peptide antigen via MHC class I | 2.47 | 0.0038 | HLA-A,B2M |
|  | GO:0036037 | CD8-positive, alpha-beta T cell activation | 2.47 | 0.0038 | HLA-E,HLA-A |
|  | GO:2000774 | Positive regulation of cellular senescence | 2.47 | 0.0038 | HLA-G,B2M |
|  | GO:0045087 | Innate immune response | 1.07 | 0.0046 | CD2,HLA-E,HLA-A,LCK,B2M |
|  | GO:0001913 | T cell mediated cytotoxicity | 2.41 | 0.0047 | HLA-A,B2M |
|  | GO:0050830 | Defense response to Gram-positive bacterium | 1.62 | 0.0063 | HLA-E,HLA-A,B2M |
|  | GO:1903706 | Regulation of hemopoiesis | 1.24 | 0.0074 | CD4,CD2,HLA-G,B2M |
|  | GO:0002507 | Tolerance induction | 2.25 | 0.0083 | HLA-E,HLA-G |
|  | GO:0007169 | Transmembrane receptor protein tyrosine kinase signaling pathway | 1.23 | 0.0083 | CD4,CD8B,CD8A,LCK |
|  | GO:0051716 | Cellular response to stimulus | 0.45 | 0.0118 | CD4,CD3D,CD8B,CD247,CD2,HLA-G,HLA-A,CD8A,LCK,B2M |
|  | GO:0002726 | Positive regulation of T cell cytokine production | 2.14 | 0.013 | HLA-A,B2M |
|  | GO:0038094 | Fc-gamma receptor signaling pathway | 2.09 | 0.0159 | CD247,LCK |
|  | GO:0042129 | Regulation of T cell proliferation | 1.46 | 0.0163 | HLA-E,HLA-G,HLA-A |
|  | GO:0045058 | T cell selection | 1.92 | 0.0314 | CD4,CD3D |
| Molecular Function | GO:0042287 | MHC protein binding | 2.34 | 1.27E-05 | CD4,CD8B,HLA-E,CD8A |
|  | GO:0042610 | CD8 receptor binding | 3.25 | 1.27E-05 | HLA-G,HLA-A,LCK |
|  | GO:0042608 | T cell receptor binding | 2.73 | 6.06E-05 | HLA-E,HLA-A,LCK |
|  | GO:0005102 | Signaling receptor binding | 0.98 | 0.00019 | CD4,CD8B,CD2,HLA-E,HLA-G,HLA-A,CD8A,LCK |
|  | GO:0042288 | MHC class I protein binding | 2.45 | 0.0002 | CD8B,HLA-E,CD8A |
|  | GO:0042605 | Peptide antigen binding | 2.35 | 0.00031 | HLA-E,HLA-G,HLA-A |
|  | GO:0023023 | MHC protein complex binding | 2.19 | 0.00076 | CD4,CD8A,B2M |
|  | GO:0015026 | Coreceptor activity | 2.05 | 0.0016 | CD4,CD8B,CD8A |
|  | GO:0030881 | beta-2-microglobulin binding | 2.71 | 0.005 | HLA-E,HLA-A |
|  | GO:0005515 | Protein binding | 0.43 | 0.0075 | CD4,CD3D,CD8B,CD247,CD2,HLA-E,HLA-G,HLA-A,CD8A,LCK,B2M |
|  | GO:0044877 | Protein-containing complex binding | 0.93 | 0.01 | CD4,HLA-E,HLA-A,CD8A,LCK,B2M |
|  | GO:0042802 | Identical protein binding | 0.77 | 0.0143 | CD4,CD3D,CD247,CD2,HLA-G,LCK,B2M |
|  | GO:1990782 | Protein tyrosine kinase binding | 1.65 | 0.0143 | CD4,CD247,CD2 |
|  | GO:0038023 | Signaling receptor activity | 0.86 | 0.0205 | CD4,CD3D,CD8B,CD247,CD2,CD8A |
|  | GO:0023026 | MHC class II protein complex binding | 2.14 | 0.0308 | CD4,B2M |
| Cellular Compartment | GO:0042101 | T cell receptor complex | 2.81 | 4.42E-10 | CD4,CD3D,CD8B,CD247,CD8A |
|  | GO:0098552 | Side of membrane | 1.42 | 1.10E-09 | CD4,CD3D,CD2,HLA-E,HLA-G,HLA-A,CD8A,LCK,B2M |
|  | GO:0098797 | Plasma membrane protein complex | 1.44 | 1.10E-09 | CD4,CD3D,CD8B,CD247,HLA-E,HLA-G,HLA-A,CD8A,B2M |
|  | GO:0042612 | MHC class I protein complex | 2.95 | 1.33E-08 | HLA-E,HLA-G,HLA-A,B2M |
|  | GO:0009986 | Cell surface | 1.26 | 1.77E-08 | CD4,CD3D,CD8B,CD2,HLA-E,HLA-G,HLA-A,CD8A,B2M |
|  | GO:0009897 | External side of plasma membrane | 1.51 | 1.29E-07 | CD4,CD3D,CD2,HLA-E,HLA-G,CD8A,B2M |
|  | GO:0030662 | Coated vesicle membrane | 1.75 | 1.29E-07 | CD4,CD3D,HLA-E,HLA-G,HLA-A,B2M |
|  | GO:0030666 | Endocytic vesicle membrane | 1.75 | 1.29E-07 | CD4,CD3D,HLA-E,HLA-G,HLA-A,B2M |
|  | GO:0098802 | Plasma membrane signaling receptor complex | 1.74 | 1.29E-07 | CD4,CD3D,CD8B,CD247,CD8A,B2M |
|  | GO:0012507 | ER to Golgi transport vesicle membrane | 2.09 | 4.21E-06 | HLA-E,HLA-G,HLA-A,B2M |
|  | GO:0031901 | Early endosome membrane | 1.7 | 4.21E-06 | CD8B,HLA-E,HLA-G,HLA-A,B2M |
|  | GO:0005769 | Early endosome | 1.42 | 4.68E-06 | CD4,CD8B,HLA-E,HLA-G,HLA-A,B2M |
|  | GO:0030670 | Phagocytic vesicle membrane | 1.97 | 9.24E-06 | HLA-E,HLA-G,HLA-A,B2M |
|  | GO:0055038 | Recycling endosome membrane | 1.88 | 1.89E-05 | HLA-E,HLA-G,HLA-A,B2M |
|  | GO:0071556 | Integral component of lumenal side of endoplasmic reticulum membrane | 2.32 | 4.57E-05 | HLA-E,HLA-G,HLA-A |
|  | GO:0030659 | Cytoplasmic vesicle membrane | 1.02 | 7.09E-05 | CD4,CD3D,CD8B,HLA-E,HLA-G,HLA-A,B2M |
|  | GO:0005886 | Plasma membrane | 0.55 | 7.15E-05 | CD4,CD3D,CD8B,CD247,CD2,HLA-E,HLA-G,HLA-A,CD8A,LCK,B2M |
|  | GO:0030176 | Integral component of endoplasmic reticulum membrane | 1.63 | 0.00013 | HLA-E,HLA-G,HLA-A,B2M |
|  | GO:0042105 | Alpha-beta T cell receptor complex | 2.86 | 0.00037 | CD3D,CD247 |
|  | GO:0042824 | MHC class I peptide loading complex | 2.6 | 0.00088 | HLA-A,B2M |
|  | GO:0045121 | Membrane raft | 1.35 | 0.0012 | CD4,CD2,CD8A,LCK |
|  | GO:0032991 | Protein-containing complex | 0.51 | 0.0013 | CD4,CD3D,CD8B,CD247,CD2,HLA-E,HLA-G,HLA-A,CD8A,B2M |
|  | GO:0016021 | Integral component of membrane | 0.5 | 0.0016 | CD4,CD3D,CD8B,CD247,CD2,HLA-E,HLA-G,HLA-A,CD8A,B2M |
|  | GO:0098588 | Bounding membrane of organelle | 0.77 | 0.0019 | CD4,CD3D,CD8B,HLA-E,HLA-G,HLA-A,B2M |
|  | GO:0005794 | Golgi apparatus | 0.81 | 0.0047 | CD247,CD2,HLA-E,HLA-G,HLA-A,B2M |
|  | GO:0005789 | Endoplasmic reticulum membrane | 0.89 | 0.0101 | CD4,HLA-E,HLA-G,HLA-A,B2M |
|  | GO:0031982 | Vesicle | 0.56 | 0.0101 | CD4,CD3D,CD8B,HLA-E,HLA-G,HLA-A,LCK,B2M |
|  | GO:0000139 | Golgi membrane | 1.03 | 0.0135 | HLA-E,HLA-G,HLA-A,B2M |
|  | GO:0005576 | Extracellular region | 0.54 | 0.0135 | CD8B,CD2,HLA-E,HLA-G,HLA-A,CD8A,LCK,B2M |
|  | GO:0030669 | Clathrin-coated endocytic vesicle membrane | 1.71 | 0.0259 | CD4,CD3D |
|  | GO:0012505 | Endomembrane system | 0.48 | 0.0311 | CD4,CD8B,CD247,CD2,HLA-E,HLA-G,HLA-A,B2M |
|  | GO:0005887 | Integral component of plasma membrane | 0.72 | 0.0486 | CD4,CD8B,CD2,HLA-A,CD8A |
| Subcellular localization | GOCC:0098797 | Plasma membrane protein complex | 1.42 | 6.55E-11 | CD4,CD3D,CD8B,CD247,HLA-E,HLA-G,HLA-A,CD8A,LCK,B2M |
|  | GOCC:0042101 | T cell receptor complex | 2.78 | 3.31E-10 | CD4,CD3D,CD8B,CD247,CD8A |
|  | GOCC:0042105 | Alpha-beta T cell receptor complex | 3.08 | 8.44E-09 | CD4,CD3D,CD247,CD8A |
|  | GOCC:0042612 | MHC class I protein complex | 2.95 | 1.23E-08 | HLA-E,HLA-G,HLA-A,B2M |
|  | GOCC:0098802 | Plasma membrane signaling receptor complex | 1.69 | 1.23E-08 | CD4,CD3D,CD8B,CD247,CD8A,LCK,B2M |
|  | GOCC:0030666 | Endocytic vesicle membrane | 1.9 | 1.78E-08 | CD4,CD3D,HLA-E,HLA-G,HLA-A,B2M |
|  | GOCC:0030662 | Coated vesicle membrane | 1.85 | 3.10E-08 | CD4,CD3D,HLA-E,HLA-G,HLA-A,B2M |
|  | GOCC:0005769 | Early endosome | 1.63 | 4.07E-07 | CD4,CD8B,HLA-E,HLA-G,HLA-A,B2M |
|  | GOCC:0031901 | Early endosome membrane | 1.93 | 4.07E-07 | CD8B,HLA-E,HLA-G,HLA-A,B2M |
|  | GOCC:0098552 | Side of membrane | 1.61 | 4.18E-07 | CD4,CD2,HLA-E,HLA-G,HLA-A,CD8A |
|  | GOCC:0005886 | Plasma membrane | 0.75 | 9.07E-07 | CD4,CD3D,CD8B,CD247,CD2,HLA-E,HLA-G,HLA-A,CD8A,LCK,B2M |
|  | GOCC:0043384 | pre-T cell receptor complex | 3.03 | 9.79E-07 | CD4,CD8A,LCK |
|  | GOCC:0042106 | Gamma-delta T cell receptor complex | 2.95 | 1.39E-06 | CD4,CD247,CD8A |
|  | GOCC:0012507 | ER to Golgi transport vesicle membrane | 2.18 | 1.57E-06 | HLA-E,HLA-G,HLA-A,B2M |
|  | GOCC:0030670 | Phagocytic vesicle membrane | 2.16 | 1.74E-06 | HLA-E,HLA-G,HLA-A,B2M |
|  | GOCC:0055038 | Recycling endosome membrane | 2.07 | 3.65E-06 | HLA-E,HLA-G,HLA-A,B2M |
|  | GOCC:0016021 | Integral component of membrane | 0.98 | 1.53E-05 | CD4,CD2,HLA-E,HLA-G,HLA-A,CD8A,LCK,B2M |
|  | GOCC:0030176 | Integral component of endoplasmic reticulum membrane | 1.8 | 3.09E-05 | HLA-E,HLA-G,HLA-A,B2M |
|  | GOCC:0071556 | Integral component of lumenal side of endoplasmic reticulum membrane | 2.32 | 3.60E-05 | HLA-E,HLA-G,HLA-A |
|  | GOCC:0009986 | Cell surface | 1.31 | 0.00013 | CD4,CD2,HLA-E,HLA-A,CD8A |
|  | GOCC:0031982 | Vesicle | 0.83 | 0.00013 | CD4,CD3D,CD8B,HLA-E,HLA-G,HLA-A,LCK,B2M |
|  | GOCC:0098588 | Bounding membrane of organelle | 0.94 | 0.00016 | CD4,CD3D,CD8B,HLA-E,HLA-G,HLA-A,B2M |
|  | GOCC:0000943 | Retrotransposon nucleocapsid | 2.95 | 0.00022 | CD4,CD8A |
|  | GOCC:0098827 | Endoplasmic reticulum subcompartment | 1.25 | 0.00023 | CD4,HLA-E,HLA-G,HLA-A,B2M |
|  | GOCC:0005789 | Endoplasmic reticulum membrane | 1.22 | 0.00031 | CD4,HLA-E,HLA-G,HLA-A,B2M |
|  | GOCC:0005615 | Extracellular space | 1.02 | 0.00036 | CD4,HLA-E,HLA-A,CD8A,LCK,B2M |
|  | GOCC:0005893 | interleukin-2 receptor complex | 2.78 | 0.00036 | CD4,CD8A |
|  | GOCC:0031410 | Cytoplasmic vesicle | 0.86 | 0.00045 | CD4,CD3D,CD8B,HLA-E,HLA-G,HLA-A,B2M |
|  | GOCC:0042824 | MHC class I peptide loading complex | 2.6 | 0.00065 | HLA-A,B2M |
|  | GOCC:0009897 | External side of plasma membrane | 1.72 | 0.00095 | CD4,CD2,CD8A |
|  | GOCC:0071745 | IgA immunoglobulin complex | 2.44 | 0.0012 | CD4,CD8A |
|  | GOCC:0071735 | IgG immunoglobulin complex | 2.41 | 0.0013 | CD4,CD8A |
|  | GOCC:0071753 | IgM immunoglobulin complex | 2.38 | 0.0014 | CD4,CD8A |
|  | GOCC:0005887 | Integral component of plasma membrane | 1.05 | 0.0015 | CD4,CD2,HLA-A,CD8A,LCK |
|  | GOCC:0070062 | Extracellular exosome | 1.22 | 0.0023 | HLA-E,HLA-A,LCK,B2M |
|  | GOCC:0099126 | Transforming growth factor beta complex | 2.25 | 0.0023 | CD4,CD8A |
|  | GOCC:0043514 | interleukin-12 complex | 2.17 | 0.0031 | CD4,CD8A |
|  | GOCC:0065010 | Extracellular membrane-bounded organelle | 1.18 | 0.0033 | HLA-E,HLA-A,LCK,B2M |
|  | GOCC:0045121 | Membrane raft | 1.47 | 0.0041 | CD4,CD8A,LCK |
|  | GOCC:0030669 | Clathrin-coated endocytic vesicle membrane | 1.71 | 0.0215 | CD4,CD3D |
|  | GOCC:0005737 | Cytoplasm | 0.34 | 0.0318 | CD4,CD3D,CD8B,CD247,CD2,HLA-E,HLA-G,HLA-A,LCK,B2M |

**Supplementary Table S5: Gene Ontology enriched terms of CD28**

| **Enriched Term** | **Enriched Term ID** | **Term description** | **Strength** | **FDR** | **Matching proteins in the network** |
| --- | --- | --- | --- | --- | --- |
| Biological Process | GO:0031295 | T cell costimulation | 2.53 | 2.45E-15 | CD80,CD28,CD86,ICOSLG,CD40LG,CD274,LCK,LOC102723996 |
|  | GO:0050671 | Positive regulation of lymphocyte proliferation | 2.04 | 2.74E-14 | CD80,CD276,CD28,CD86,ICOSLG,CD40LG,CD40,CD274,LOC102723996 |
|  | GO:0051251 | Positive regulation of lymphocyte activation | 1.73 | 6.91E-14 | CD80,CD276,CD28,CD86,ICOSLG,CD40LG,CD40,CD274,LCK,LOC102723996 |
|  | GO:0042102 | Positive regulation of T cell proliferation | 2.12 | 3.19E-13 | CD80,CD276,CD28,CD86,ICOSLG,CD40LG,CD274,LOC102723996 |
|  | GO:0050870 | Positive regulation of T cell activation | 1.81 | 7.40E-13 | CD80,CD276,CD28,CD86,ICOSLG,CD40LG,CD274,LCK,LOC102723996 |
|  | GO:0050778 | Positive regulation of immune response | 1.51 | 1.69E-10 | CD80,CD276,CD28,CD86,ICOSLG,CD40,CD274,LCK,LOC102723996 |
|  | GO:0001817 | Regulation of cytokine production | 1.34 | 4.83E-09 | CD80,CD276,CD28,CD86,ICOSLG,CD40LG,CD40,CD274,LOC102723996 |
|  | GO:0046649 | Lymphocyte activation | 1.5 | 7.85E-09 | CD276,CD28,CD86,ICOSLG,CD40LG,CD40,LCK,LOC102723996 |
|  | GO:0007166 | Cell surface receptor signaling pathway | 0.98 | 8.18E-09 | CD80,CD276,CD28,CD86,ICOSLG,CD40LG,CD40,CD274,GRB2,LCK,LOC102723996 |
|  | GO:0042130 | Negative regulation of T cell proliferation | 2.08 | 1.97E-07 | CD80,CD86,ICOSLG,CD274,LOC102723996 |
|  | GO:0002768 | Immune response-regulating cell surface receptor signaling pathway | 1.67 | 4.89E-07 | CD276,CD28,ICOSLG,CD40,LCK,LOC102723996 |
|  | GO:0006955 | Immune response | 1.09 | 5.52E-07 | CD80,CD28,CD86,ICOSLG,CD40LG,CD40,CD274,LCK,LOC102723996 |
|  | GO:0001819 | Positive regulation of cytokine production | 1.42 | 6.58E-07 | CD80,CD276,CD28,CD86,CD40LG,CD40,CD274 |
|  | GO:0050852 | T cell receptor signaling pathway | 1.94 | 7.56E-07 | CD276,CD28,ICOSLG,LCK,LOC102723996 |
|  | GO:0002376 | Immune system process | 0.93 | 7.75E-07 | CD80,CD276,CD28,CD86,ICOSLG,CD40LG,CD40,CD274,LCK,LOC102723996 |
|  | GO:0002250 | Adaptive immune response | 1.48 | 5.29E-06 | CD86,ICOSLG,CD40LG,CD40,CD274,LOC102723996 |
|  | GO:0023035 | CD40 signaling pathway | 2.73 | 1.13E-05 | CD86,CD40LG,CD40 |
|  | GO:0042113 | B cell activation | 1.68 | 1.17E-05 | CD86,ICOSLG,CD40LG,CD40,LOC102723996 |
|  | GO:0071222 | Cellular response to lipopolysaccharide | 1.66 | 1.31E-05 | CD80,CD86,ICOSLG,CD274,LOC102723996 |
|  | GO:0002637 | Regulation of immunoglobulin production | 1.97 | 2.12E-05 | CD28,CD86,CD40LG,CD40 |
|  | GO:2000514 | Regulation of CD4-positive, alpha-beta T cell activation | 1.97 | 2.20E-05 | CD80,CD28,CD86,CD274 |
|  | GO:0042110 | T cell activation | 1.48 | 8.21E-05 | CD276,CD28,ICOSLG,LCK,LOC102723996 |
|  | GO:0032753 | Positive regulation of interleukin-4 production | 2.3 | 0.00011 | CD28,CD86,CD40LG |
|  | GO:0048583 | Regulation of response to stimulus | 0.66 | 0.00019 | CD80,CD276,CD28,CD86,ICOSLG,CD40,CD274,GRB2,LCK,LOC102723996 |
|  | GO:0048522 | Positive regulation of cellular process | 0.55 | 0.0002 | CD80,CD276,CD28,CD86,ICOSLG,CD40LG,CD40,CD274,GRB2,LCK,LOC102723996 |
|  | GO:0032743 | Positive regulation of interleukin-2 production | 2.17 | 0.00023 | CD80,CD28,CD86 |
|  | GO:0002697 | Regulation of immune effector process | 1.37 | 0.00024 | CD80,CD28,CD86,CD40LG,CD40 |
|  | GO:0032733 | Positive regulation of interleukin-10 production | 2.13 | 0.00031 | CD28,CD40LG,CD274 |
|  | GO:0051707 | Response to other organism | 0.97 | 0.00032 | CD80,CD86,ICOSLG,CD40,CD274,LCK,LOC102723996 |
|  | GO:2000516 | Positive regulation of CD4-positive, alpha-beta T cell activation | 2.12 | 0.00032 | CD80,CD28,CD86 |
|  | GO:0002822 | Regulation of adaptive immune response based on somatic recombination of immune receptors built from immunoglobulin superfamily domains | 1.6 | 0.0004 | CD80,CD28,CD40,CD274 |
|  | GO:0046006 | Regulation of activated T cell proliferation | 2.08 | 0.0004 | ICOSLG,CD274,LOC102723996 |
|  | GO:0002639 | Positive regulation of immunoglobulin production | 2.01 | 0.0006 | CD28,CD86,CD40 |
|  | GO:0019222 | Regulation of metabolic process | 0.46 | 0.0013 | CD80,CD276,CD28,CD86,ICOSLG,CD40LG,CD40,CD274,GRB2,LCK,LOC102723996 |
|  | GO:1901701 | Cellular response to oxygen-containing compound | 1.01 | 0.0014 | CD80,CD86,ICOSLG,CD274,GRB2,LOC102723996 |
|  | GO:0002699 | Positive regulation of immune effector process | 1.43 | 0.0016 | CD80,CD28,CD86,CD40 |
|  | GO:0009893 | Positive regulation of metabolic process | 0.62 | 0.0024 | CD80,CD276,CD28,CD86,CD40LG,CD40,CD274,GRB2,LCK |
|  | GO:0048304 | Positive regulation of isotype switching to IgG isotypes | 2.55 | 0.0028 | CD28,CD40 |
|  | GO:0030168 | Platelet activation | 1.74 | 0.003 | CD40LG,CD40,LCK |
|  | GO:0071310 | Cellular response to organic substance | 0.79 | 0.004 | CD80,CD86,ICOSLG,CD40,CD274,GRB2,LOC102723996 |
|  | GO:0002824 | Positive regulation of adaptive immune response based on somatic recombination of immune receptors built from immunoglobulin superfamily domains | 1.69 | 0.0042 | CD28,CD40,CD274 |
|  | GO:0046651 | Lymphocyte proliferation | 1.64 | 0.0058 | CD28,CD40LG,CD40 |
|  | GO:2000353 | Positive regulation of endothelial cell apoptotic process | 2.28 | 0.0079 | CD40LG,CD40 |
|  | GO:0051247 | Positive regulation of protein metabolic process | 0.85 | 0.009 | CD80,CD28,CD86,CD40LG,CD40,LCK |
|  | GO:0045624 | Positive regulation of T-helper cell differentiation | 2.21 | 0.01 | CD80,CD86 |
|  | GO:0060255 | Regulation of macromolecule metabolic process | 0.46 | 0.0104 | CD80,CD276,CD28,CD86,ICOSLG,CD40LG,CD40,CD274,LCK,LOC102723996 |
|  | GO:0006972 | Hyperosmotic response | 2.17 | 0.0116 | ICOSLG,LOC102723996 |
|  | GO:2000561 | Regulation of CD4-positive, alpha-beta T cell proliferation | 2.17 | 0.0116 | CD28,CD274 |
|  | GO:0010604 | Positive regulation of macromolecule metabolic process | 0.61 | 0.0132 | CD80,CD276,CD28,CD86,CD40LG,CD40,CD274,LCK |
|  | GO:0042104 | Positive regulation of activated T cell proliferation | 2.11 | 0.0145 | ICOSLG,LOC102723996 |
|  | GO:0045580 | Regulation of T cell differentiation | 1.48 | 0.0145 | CD80,CD28,CD86 |
|  | GO:0043065 | Positive regulation of apoptotic process | 1.15 | 0.0149 | CD40LG,CD40,CD274,LCK |
|  | GO:0042221 | Response to chemical | 0.55 | 0.0292 | CD80,CD86,ICOSLG,CD40,CD274,GRB2,LCK,LOC102723996 |
|  | GO:0032735 | Positive regulation of interleukin-12 production | 1.92 | 0.0295 | CD40LG,CD40 |
|  | GO:0042100 | B cell proliferation | 1.83 | 0.043 | CD40LG,CD40 |
|  | GO:0043491 | Protein kinase B signaling | 1.83 | 0.043 | CD28,CD40 |
|  | GO:0030098 | Lymphocyte differentiation | 1.28 | 0.0447 | CD28,CD40LG,LCK |
| Molecular function | GO:0015026 | Coreceptor activity | 2.05 | 0.0131 | CD80,CD28,CD86 |
| Cellular compartment | GO:0098552 | Side of membrane | 1.47 | 1.97E-11 | CD80,CD276,CD28,CD86,ICOSLG,CD40LG,CD40,CD274,LCK,LOC102723996 |
|  | GO:0009897 | External side of plasma membrane | 1.62 | 2.71E-11 | CD80,CD276,CD28,CD86,ICOSLG,CD40LG,CD40,CD274,LOC102723996 |
|  | GO:0005886 | Plasma membrane | 0.55 | 0.00045 | CD80,CD276,CD28,CD86,ICOSLG,CD40LG,CD40,CD274,GRB2,LCK,LOC102723996 |
|  | GO:0005615 | Extracellular space | 0.64 | 0.0119 | CD86,ICOSLG,CD40LG,CD40,CD274,GRB2,LCK,LOC102723996 |
|  | GO:0070062 | Extracellular exosome | 0.78 | 0.0119 | CD86,ICOSLG,CD40,CD274,GRB2,LCK,LOC102723996 |
| Subcellular localization |  |  |  |  |  |
|  | GOCC:0065010 | Extracellular membrane-bounded organelle | 1.42 | 1.71E-06 | CD86,ICOSLG,CD40,CD274,GRB2,LCK,LOC102723996 |
|  | GOCC:0070062 | Extracellular exosome | 1.47 | 1.71E-06 | CD86,ICOSLG,CD40,CD274,GRB2,LCK,LOC102723996 |
|  | GOCC:0005615 | Extracellular space | 1.14 | 3.71E-06 | CD80,CD86,ICOSLG,CD40,CD274,GRB2,LCK,LOC102723996 |
|  | GOCC:0009986 | Cell surface | 1.39 | 2.03E-05 | CD80,CD28,CD86,CD40LG,CD40,CD274 |
|  | GOCC:0005576 | Extracellular region | 0.89 | 2.45E-05 | CD80,CD86,ICOSLG,CD40LG,CD40,CD274,GRB2,LCK,LOC102723996 |
|  | GOCC:0005886 | Plasma membrane | 0.7 | 9.28E-05 | CD80,CD276,CD28,CD86,ICOSLG,CD40LG,CD40,CD274,GRB2,LCK |
|  | GOCC:0016020 | Membrane | 0.54 | 0.00028 | CD80,CD276,CD28,CD86,ICOSLG,CD40LG,CD40,CD274,GRB2,LCK,LOC102723996 |
|  | GOCC:0016021 | Integral component of membrane | 0.92 | 0.00082 | CD276,CD28,ICOSLG,CD40LG,CD40,LCK,LOC102723996 |
|  | GOCC:0043514 | interleukin-12 complex | 2.17 | 0.015 | CD80,CD86 |
|  | GOCC:0001772 | Immunological synapse | 2 | 0.0302 | CD28,LCK |
|  | GOCC:0098802 | Plasma membrane signaling receptor complex | 1.32 | 0.0487 | CD28,CD40,LCK |

**Supplementary Table S6: Gene Ontology enriched terms of CD137**

| **Enriched Term** | **Enriched Term ID** | **Term description** | **Strength** | **FDR** | **Matching proteins in the network** |
| --- | --- | --- | --- | --- | --- |
| Biological Process | GO:0042129 | Regulation of T cell proliferation | 1.94 | 6.70E-13 | TNFSF9,CD80,TNFSF4,CD28,ICOSLG,LGALS9,CD70,TNFRSF9,LOC102723996 |
|  | GO:0042102 | Positive regulation of T cell proliferation | 2.12 | 1.44E-12 | TNFSF9,CD80,TNFSF4,CD28,ICOSLG,LGALS9,CD70,LOC102723996 |
|  | GO:0002684 | Positive regulation of immune system process | 1.27 | 2.53E-08 | TNFSF9,CD80,TNFSF4,CD28,ICOSLG,CD247,LGALS9,CD70,LOC102723996 |
|  | GO:0002682 | Regulation of immune system process | 1.1 | 3.12E-08 | TNFSF9,CD80,TNFSF4,CD28,ICOSLG,CD247,LGALS9,CD70,TNFRSF9,LOC102723996 |
|  | GO:0006955 | Immune response | 1.09 | 8.62E-07 | TNFSF9,CD80,TNFSF4,CD28,ICOSLG,CD247,LGALS9,CD70,LOC102723996 |
|  | GO:0050778 | Positive regulation of immune response | 1.4 | 1.34E-06 | CD80,TNFSF4,CD28,ICOSLG,CD247,LGALS9,LOC102723996 |
|  | GO:0050868 | Negative regulation of T cell activation | 1.84 | 3.37E-06 | CD80,TNFSF4,ICOSLG,LGALS9,LOC102723996 |
|  | GO:2000516 | Positive regulation of CD4-positive, alpha-beta T cell activation | 2.24 | 4.18E-06 | CD80,TNFSF4,CD28,LGALS9 |
|  | GO:0031295 | T cell costimulation | 2.23 | 4.42E-06 | CD80,CD28,ICOSLG,LOC102723996 |
|  | GO:0046006 | Regulation of activated T cell proliferation | 2.2 | 5.14E-06 | TNFSF9,ICOSLG,LGALS9,LOC102723996 |
|  | GO:0045580 | Regulation of T cell differentiation | 1.7 | 1.26E-05 | TNFSF9,CD80,TNFSF4,CD28,LGALS9 |
|  | GO:0046649 | Lymphocyte activation | 1.37 | 2.60E-05 | TNFSF4,CD28,ICOSLG,CD247,CD70,LOC102723996 |
|  | GO:0042130 | Negative regulation of T cell proliferation | 1.98 | 2.88E-05 | CD80,ICOSLG,LGALS9,LOC102723996 |
|  | GO:0050852 | T cell receptor signaling pathway | 1.84 | 8.09E-05 | CD28,ICOSLG,CD247,LOC102723996 |
|  | GO:0042110 | T cell activation | 1.48 | 0.00011 | TNFSF4,CD28,ICOSLG,CD247,LOC102723996 |
|  | GO:0045582 | Positive regulation of T cell differentiation | 1.79 | 0.00012 | TNFSF9,CD80,TNFSF4,LGALS9 |
|  | GO:0032496 | Response to lipopolysaccharide | 1.46 | 0.00013 | CD80,TNFSF4,ICOSLG,LGALS9,LOC102723996 |
|  | GO:0032753 | Positive regulation of interleukin-4 production | 2.3 | 0.00014 | TNFSF4,CD28,LGALS9 |
|  | GO:0042104 | Positive regulation of activated T cell proliferation | 2.28 | 0.00015 | TNFSF9,ICOSLG,LOC102723996 |
|  | GO:0046641 | Positive regulation of alpha-beta T cell proliferation | 2.27 | 0.00016 | TNFSF4,CD28,LGALS9 |
|  | GO:0043372 | Positive regulation of CD4-positive, alpha-beta T cell differentiation | 2.21 | 0.00022 | CD80,TNFSF4,LGALS9 |
|  | GO:0045589 | Regulation of regulatory T cell differentiation | 2.2 | 0.00024 | TNFSF4,CD28,LGALS9 |
|  | GO:0001817 | Regulation of cytokine production | 1.16 | 0.00026 | CD80,TNFSF4,CD28,ICOSLG,LGALS9,LOC102723996 |
|  | GO:0032733 | Positive regulation of interleukin-10 production | 2.13 | 0.00036 | TNFSF4,CD28,LGALS9 |
|  | GO:0048584 | Positive regulation of response to stimulus | 0.83 | 0.0005 | CD80,TNFSF4,CD28,ICOSLG,CD247,TRAF1,LGALS9,LOC102723996 |
|  | GO:0071222 | Cellular response to lipopolysaccharide | 1.57 | 0.00065 | CD80,TNFSF4,ICOSLG,LOC102723996 |
|  | GO:0007165 | Signal transduction | 0.58 | 0.001 | TNFSF9,CD80,TNFSF4,CD28,ICOSLG,CD247,TRAF1,LGALS9,CD70,LOC102723996 |
|  | GO:0002699 | Positive regulation of immune effector process | 1.43 | 0.0018 | CD80,TNFSF4,CD28,LGALS9 |
|  | GO:0045625 | Regulation of T-helper 1 cell differentiation | 2.55 | 0.0032 | CD80,TNFSF4 |
|  | GO:2000563 | Positive regulation of CD4-positive, alpha-beta T cell proliferation | 2.55 | 0.0032 | CD28,LGALS9 |
|  | GO:0046631 | Alpha-beta T cell activation | 1.74 | 0.0034 | TNFSF4,CD28,CD247 |
|  | GO:0008285 | Negative regulation of cell population proliferation | 1.1 | 0.004 | CD80,ICOSLG,LGALS9,TNFRSF9,LOC102723996 |
|  | GO:0048522 | Positive regulation of cellular process | 0.51 | 0.0044 | TNFSF9,CD80,TNFSF4,CD28,ICOSLG,CD247,TRAF1,LGALS9,CD70,LOC102723996 |
|  | GO:0071310 | Cellular response to organic substance | 0.79 | 0.0044 | CD80,TNFSF4,ICOSLG,TRAF1,LGALS9,CD70,LOC102723996 |
|  | GO:0007166 | Cell surface receptor signaling pathway | 0.79 | 0.0046 | CD80,CD28,ICOSLG,CD247,TRAF1,CD70,LOC102723996 |
|  | GO:0002250 | Adaptive immune response | 1.3 | 0.0053 | ICOSLG,CD247,CD70,LOC102723996 |
|  | GO:0032736 | Positive regulation of interleukin-13 production | 2.41 | 0.0053 | TNFSF4,LGALS9 |
|  | GO:0045624 | Positive regulation of T-helper cell differentiation | 2.21 | 0.0115 | CD80,TNFSF4 |
|  | GO:0006972 | Hyperosmotic response | 2.17 | 0.0134 | ICOSLG,LOC102723996 |
|  | GO:0001819 | Positive regulation of cytokine production | 1.17 | 0.015 | CD80,TNFSF4,CD28,LGALS9 |
|  | GO:0002706 | Regulation of lymphocyte mediated immunity | 1.48 | 0.0163 | TNFSF4,CD28,LGALS9 |
|  | GO:0002822 | Regulation of adaptive immune response based on somatic recombination of immune receptors built from immunoglobulin superfamily domains | 1.48 | 0.0169 | CD80,TNFSF4,CD28 |
|  | GO:0045830 | Positive regulation of isotype switching | 2.11 | 0.017 | TNFSF4,CD28 |
|  | GO:0042113 | B cell activation | 1.45 | 0.0193 | ICOSLG,CD70,LOC102723996 |
|  | GO:0032743 | Positive regulation of interleukin-2 production | 2 | 0.0257 | CD80,CD28 |
|  | GO:2000515 | Negative regulation of CD4-positive, alpha-beta T cell activation | 2 | 0.0257 | TNFSF4,LGALS9 |
|  | GO:0051239 | Regulation of multicellular organismal process | 0.66 | 0.0262 | TNFSF9,CD80,TNFSF4,CD28,ICOSLG,LGALS9,LOC102723996 |
|  | GO:0032735 | Positive regulation of interleukin-12 production | 1.92 | 0.0339 | TNFSF4,LGALS9 |
|  | GO:0032689 | Negative regulation of interferon-gamma production | 1.91 | 0.0349 | TNFSF4,LGALS9 |
| Molecular Function | GO:0005164 | Tumor necrosis factor receptor binding | 2.35 | 1.51E-05 | TNFSF9,TNFSF4,TRAF1,CD70 |
| Cellular Compartment | GO:0098552 | Side of membrane | 1.25 | 0.00076 | CD80,CD28,ICOSLG,TRAF1,TNFRSF9,LOC102723996 |
|  | GO:0009897 | External side of plasma membrane | 1.36 | 0.0013 | CD80,CD28,ICOSLG,TNFRSF9,LOC102723996 |
|  | GO:0071944 | Cell periphery | 0.52 | 0.0015 | TNFSF9,CD80,TNFSF4,CD28,ICOSLG,CD247,TRAF1,LGALS9,CD70,TNFRSF9,LOC102723996 |
|  | GO:0009986 | Cell surface | 1.08 | 0.0017 | CD80,TNFSF4,CD28,ICOSLG,TNFRSF9,LOC102723996 |
|  | GO:0005886 | Plasma membrane | 0.51 | 0.0105 | TNFSF9,CD80,TNFSF4,CD28,ICOSLG,CD247,TRAF1,CD70,TNFRSF9,LOC102723996 |
| Subcellular localization | GOCC:0005615 | Extracellular space | 1.02 | 0.0173 | CD80,TNFSF4,ICOSLG,LGALS9,CD70,LOC102723996 |
|  | GOCC:0098636 | Protein complex involved in cell adhesion | 1.8 | 0.0173 | CD80,CD28,LGALS9 |
|  | GOCC:0042101 | T cell receptor complex | 2.38 | 0.0294 | CD28,CD247 |
|  | GOCC:0016021 | Integral component of membrane | 0.85 | 0.0384 | TNFSF4,CD28,ICOSLG,CD70,TNFRSF9,LOC102723996 |

**Supplementary Table S7: Gene Ontology enriched terms of CD3D (CD3ζ)**

| **Enriched Term** | **Enriched Term ID** | **Term description** | **Strength** | **FDR** | **Matching proteins in the network** |
| --- | --- | --- | --- | --- | --- |
| Biological Process | GO:0042110 | T cell activation | 1.78 | 1.28E-13 | CD4,ZAP70,CD3D,CD8B,CD3E,CD247,CD2,CD8A,CD3G,LCK |
|  | GO:0050852 | T cell receptor signaling pathway | 2.14 | 9.99E-13 | ZAP70,CD3D,CD8B,CD3E,CD247,CD8A,CD3G,LCK |
|  | GO:0002684 | Positive regulation of immune system process | 1.35 | 5.41E-12 | CD4,ZAP70,TRAT1,CD3D,CD8B,CD3E,CD247,CD2,CD8A,CD3G,LCK |
|  | GO:0050778 | Positive regulation of immune response | 1.55 | 5.41E-12 | CD4,ZAP70,TRAT1,CD3D,CD8B,CD3E,CD247,CD8A,CD3G,LCK |
|  | GO:0002250 | Adaptive immune response | 1.65 | 2.61E-11 | CD4,ZAP70,TRAT1,CD3D,CD8B,CD3E,CD247,CD8A,CD3G |
|  | GO:0006955 | Immune response | 1.17 | 1.56E-10 | CD4,ZAP70,TRAT1,CD3D,CD8B,CD3E,CD247,CD2,CD8A,CD3G,LCK |
|  | GO:0030217 | T cell differentiation | 1.87 | 1.21E-09 | CD4,ZAP70,CD3D,CD3E,CD8A,CD3G,LCK |
|  | GO:0007166 | Cell surface receptor signaling pathway | 0.98 | 1.25E-08 | CD4,ZAP70,TRAT1,CD3D,CD8B,CD3E,CD247,CD2,CD8A,CD3G,LCK |
|  | GO:0045058 | T cell selection | 2.32 | 2.36E-08 | CD4,ZAP70,CD3D,CD3E,CD3G |
|  | GO:0045059 | Positive thymic T cell selection | 2.81 | 4.87E-08 | ZAP70,CD3D,CD3E,CD3G |
|  | GO:0007169 | Transmembrane receptor protein tyrosine kinase signaling pathway | 1.47 | 4.11E-07 | CD4,ZAP70,TRAT1,CD8B,CD3E,CD8A,LCK |
|  | GO:0046631 | Alpha-beta T cell activation | 1.97 | 8.49E-07 | ZAP70,CD3D,CD3E,CD247,CD3G |
|  | GO:0050850 | Positive regulation of calcium-mediated signaling | 2.23 | 4.28E-06 | CD4,ZAP70,TRAT1,CD3E |
|  | GO:0046629 | Gamma-delta T cell activation | 2.5 | 6.05E-05 | CD3E,CD247,CD3G |
|  | GO:0050863 | Regulation of T cell activation | 1.38 | 0.0004 | CD4,ZAP70,CD3E,CD2,LCK |
|  | GO:0002696 | Positive regulation of leukocyte activation | 1.37 | 0.00044 | CD4,ZAP70,CD3E,CD2,LCK |
|  | GO:0018108 | Peptidyl-tyrosine phosphorylation | 1.61 | 0.00072 | CD4,ZAP70,CD3E,LCK |
|  | GO:0050870 | Positive regulation of T cell activation | 1.46 | 0.0026 | CD4,ZAP70,CD3E,LCK |
|  | GO:0045060 | Negative thymic T cell selection | 2.51 | 0.0063 | ZAP70,CD3E |
|  | GO:0050862 | Positive regulation of T cell receptor signaling pathway | 2.35 | 0.0111 | TRAT1,LCK |
|  | GO:0019722 | Calcium-mediated signaling | 1.5 | 0.0255 | CD4,ZAP70,CD3E |
|  | GO:1902533 | Positive regulation of intracellular signal transduction | 0.95 | 0.0285 | CD4,ZAP70,TRAT1,CD3E,LCK |
|  | GO:0038094 | Fc-gamma receptor signaling pathway | 2.09 | 0.0306 | CD247,LCK |
|  | GO:0046641 | Positive regulation of alpha-beta T cell proliferation | 2.09 | 0.0306 | ZAP70,CD3E |
|  | GO:0032743 | Positive regulation of interleukin-2 production | 2 | 0.0448 | CD4,CD3E |
| Molecular Function | GO:0005102 | Signaling receptor binding | 1.03 | 2.07E-05 | CD4,ZAP70,TRAT1,CD8B,CD3E,CD2,CD8A,CD3G,LCK |
|  | GO:0042608 | T cell receptor binding | 2.73 | 9.09E-05 | CD3E,CD3G,LCK |
|  | GO:0038023 | Signaling receptor activity | 0.98 | 0.00024 | CD4,CD3D,CD8B,CD3E,CD247,CD2,CD8A,CD3G |
|  | GO:1990782 | Protein tyrosine kinase binding | 1.78 | 0.00047 | CD4,TRAT1,CD247,CD2 |
|  | GO:0019901 | Protein kinase binding | 1.18 | 0.00068 | CD4,TRAT1,CD3E,CD247,CD2,LCK |
|  | GO:0042287 | MHC protein binding | 2.21 | 0.00068 | CD4,CD8B,CD8A |
|  | GO:0015026 | Coreceptor activity | 2.05 | 0.0015 | CD4,CD8B,CD8A |
|  | GO:0030159 | Signaling receptor complex adaptor activity | 2.01 | 0.0016 | TRAT1,CD3E,CD3G |
|  | GO:0005515 | Protein binding | 0.43 | 0.0069 | CD4,ZAP70,TRAT1,CD3D,CD8B,CD3E,CD247,CD2,CD8A,CD3G,LCK |
|  | GO:0042802 | Identical protein binding | 0.77 | 0.0154 | CD4,CD3D,CD3E,CD247,CD2,CD3G,LCK |
|  | GO:0042288 | MHC class I protein binding | 2.28 | 0.0208 | CD8B,CD8A |
| Cellular Compartment | GO:0042101 | T cell receptor complex | 3.06 | 7.44E-23 | CD4,ZAP70,TRAT1,CD3D,CD8B,CD3E,CD247,CD8A,CD3G |
|  | GO:0042105 | Alpha-beta T cell receptor complex | 3.16 | 2.70E-09 | CD3D,CD3E,CD247,CD3G |
|  | GO:0098552 | Side of membrane | 1.37 | 4.69E-08 | CD4,ZAP70,CD3D,CD3E,CD2,CD8A,CD3G,LCK |
|  | GO:0042106 | Gamma-delta T cell receptor complex | 3.25 | 6.61E-07 | CD3E,CD247,CD3G |
|  | GO:0009897 | External side of plasma membrane | 1.44 | 5.93E-06 | CD4,CD3D,CD3E,CD2,CD8A,CD3G |
|  | GO:0009986 | Cell surface | 1.15 | 2.34E-05 | CD4,CD3D,CD8B,CD3E,CD2,CD8A,CD3G |
|  | GO:0005886 | Plasma membrane | 0.55 | 0.00016 | CD4,ZAP70,TRAT1,CD3D,CD8B,CD3E,CD247,CD2,CD8A,CD3G,LCK |
|  | GO:0001772 | Immunological synapse | 2.1 | 0.00033 | ZAP70,CD3E,LCK |
|  | GO:0005887 | Integral component of plasma membrane | 0.87 | 0.0012 | CD4,TRAT1,CD8B,CD3E,CD2,CD8A,CD3G |
|  | GO:0030669 | Clathrin-coated endocytic vesicle membrane | 1.89 | 0.0012 | CD4,CD3D,CD3G |
|  | GO:0032991 | Protein-containing complex | 0.51 | 0.0025 | CD4,ZAP70,TRAT1,CD3D,CD8B,CD3E,CD247,CD2,CD8A,CD3G |
|  | GO:0045121 | Membrane raft | 1.35 | 0.0025 | CD4,CD2,CD8A,LCK |
|  | GO:0009898 | Cytoplasmic side of plasma membrane | 1.49 | 0.0104 | ZAP70,CD2,LCK |
|  | GO:0016021 | Integral component of membrane | 0.45 | 0.0301 | CD4,TRAT1,CD3D,CD8B,CD3E,CD247,CD2,CD8A,CD3G |
| Subcellular localization | GOCC:0042101 | T cell receptor complex | 3.03 | 1.34E-22 | CD4,ZAP70,TRAT1,CD3D,CD8B,CD3E,CD247,CD8A,CD3G |
|  | GOCC:0098802 | Plasma membrane signaling receptor complex | 1.85 | 2.04E-15 | CD4,ZAP70,TRAT1,CD3D,CD8B,CD3E,CD247,CD8A,CD3G,LCK |
|  | GOCC:0042105 | Alpha-beta T cell receptor complex | 3.25 | 4.02E-15 | CD4,CD3D,CD3E,CD247,CD8A,CD3G |
|  | GOCC:0042106 | Gamma-delta T cell receptor complex | 3.17 | 3.96E-12 | CD4,CD3E,CD247,CD8A,CD3G |
|  | GOCC:0005886 | Plasma membrane | 0.75 | 1.81E-06 | CD4,ZAP70,TRAT1,CD3D,CD8B,CD3E,CD247,CD2,CD8A,CD3G,LCK |
|  | GOCC:0043384 | pre-T cell receptor complex | 3.03 | 1.85E-06 | CD4,CD8A,LCK |
|  | GOCC:0009897 | External side of plasma membrane | 1.85 | 5.30E-05 | CD4,CD3E,CD2,CD8A |
|  | GOCC:0005887 | Integral component of plasma membrane | 1.13 | 0.0003 | CD4,CD3E,CD2,CD8A,CD3G,LCK |
|  | GOCC:0000943 | Retrotransposon nucleocapsid | 2.95 | 0.00065 | CD4,CD8A |
|  | GOCC:0005893 | interleukin-2 receptor complex | 2.78 | 0.0011 | CD4,CD8A |
|  | GOCC:0030669 | Clathrin-coated endocytic vesicle membrane | 1.89 | 0.0011 | CD4,CD3D,CD3G |
|  | GOCC:0071745 | IgA immunoglobulin complex | 2.44 | 0.0031 | CD4,CD8A |
|  | GOCC:0071735 | IgG immunoglobulin complex | 2.41 | 0.0034 | CD4,CD8A |
|  | GOCC:0071753 | IgM immunoglobulin complex | 2.38 | 0.0037 | CD4,CD8A |
|  | GOCC:0099126 | Transforming growth factor beta complex | 2.25 | 0.0058 | CD4,CD8A |
|  | GOCC:0043514 | interleukin-12 complex | 2.17 | 0.0068 | CD4,CD8A |
|  | GOCC:0045121 | Membrane raft | 1.47 | 0.0087 | CD4,CD8A,LCK |
|  | GOCC:0001772 | Immunological synapse | 2 | 0.0129 | ZAP70,LCK |
